# Action of tyrosinase on alpha and beta-arbutin: A kinetic study

**DOI:** 10.1371/journal.pone.0177330

**Published:** 2017-05-11

**Authors:** Antonio Garcia-Jimenez, Jose Antonio Teruel-Puche, Jose Berna, José Neptuno Rodriguez-Lopez, Jose Tudela, Francisco Garcia-Canovas

**Affiliations:** 1 GENZ-Group of research on Enzymology, Department of Biochemistry and Molecular Biology-A, Regional Campus of International Excellence "Campus Mare Nostrum", University of Murcia, Espinardo, Murcia, Spain; 2 Group of Molecular Interactions in Membranes, Department of Biochemistry and Molecular Biology-A, University of Murcia, Espinardo, Murcia, Spain; 3 Group of Synthetic Organic Chemistry, Department of Organic Chemistry, Faculty of Chemistry, University of Murcia, Espinardo, Murcia, Spain; Wageningen Universiteit, NETHERLANDS

## Abstract

The known derivatives from hydroquinone, α and β-arbutin, are used as depigmenting agents. In this work, we demonstrate that the *oxy* form of tyrosinase (oxytyrosinase) hydroxylates α and β-arbutin in *ortho* position of the phenolic hydroxyl group, giving rise to a complex formed by *met*-tyrosinase with the hydroxylated α or β-arbutin. This complex could evolve in two ways: by oxidizing the originated *o*-diphenol to *o*-quinone and *deoxy*-tyrosinase, or by delivering the *o*-diphenol and *met*-tyrosinase to the medium, which would produce the self-activation of the system. Note that the quinones generated in both cases are unstable, so the catalysis cannot be studied quantitatively. However, if 3-methyl-2-benzothiazolinone hydrazone hydrochloride hydrate is used, the *o*-quinone is attacked, so that it becomes an adduct, which can be oxidized by another molecule of *o*-quinone, generating *o*-diphenol in the medium. In this way, the system reaches the steady state and originates a chromophore, which, in turn, has a high absorptivity in the visible spectrum. This reaction allowed us to characterize α and β-arbutin kinetically as substrates of tyrosinase for the first time, obtaining a Michaelis constant values of 6.5 ± 0.58 mM and 3 ± 0.19 mM, respectively. The data agree with those from docking studies that showed that the enzyme has a higher affinity for β-arbutin. Moreover, the catalytic constants obtained by the kinetic studies (catalytic constant = 4.43 ± 0.33 s^-1^ and 3.7 ± 0.29 s^-1^ for α and β-arbutin respectively) agree with our forecast based on 13 C NMR considerations. This kinetic characterization of α and β-arbutin as substrates of tyrosinase should be taken into account to explain possible adverse effects of these compounds.

## Introduction

Tyrosinase (EC 1.14.18.1) is a copper enzyme widely distributed in nature. It is involved in the production of melanin, which causes pigmentation of the skin, protecting it from UV-induced damage. The enzyme catalyzes two types of reaction: (a) the *ortho*-hydroxylation of monophenols, rendering into *o*-diphenols (monophenolase activity), and (b) the oxidation of *o*-diphenols to *o*-quinones (diphenolase activity). Both of them use molecular oxygen as cosubstrate [1].

However, melanin causes the browning of fruits, vegetables, fungi and crustaceans, which harms their quality and organoleptic properties, reducing their commercial value [2]. It can also produce hyperpigmentation disorders such as ephelide, freckles, solar lentigines and melasma [2], which are treated with inhibitors of tyrosinase [2–4].

Some compounds with resorcinol structure, such as 4-hexylresorcinol [5], 4-*n*-butylresorcinol [6] or oxyresveratrol [7], and others such as ellagic acid [8], are used as inhibitors of the enzyme in cosmetics and pharmaceutical industries [2]. However, they have recently been described as substrates, since they react with tyrosinase, producing reactive quinones [2,9–12]. These compounds can be attacked by *E*_ox_ (*oxy*-tyrosinase), but not by *E*_m_ (metatyrosinase), as can monophenols, so the *oxy* form of the enzyme must be produced first. For that purpose, it is necessary to add some certain compounds to the medium, for example: a) a reductant, such as ascorbic acid (AH_2_), to convert *E*_m_ to *E*_d_ (*deoxy*-tyrosinase), which evolves to *E*_ox_ in the presence of oxygen; b) hydrogen peroxide (H_2_O_2_), to transform *E*_m_ into *E*_ox_ directly; c) *o*-diphenol (only necessary in catalytic quantities if there is ascorbic acid to keep the quantity of *o*-diphenol constant in the reaction medium) to produce the conversion of *E*_m_ to *E*_d_, which, with oxygen, becomes *E*_ox_ [13].

Hydroquinone (HQ) inhibits the melanogenesis process very effectively, so it is used as a depigmenting agent [14,15]. The action of tyrosinase on this compound has given rise to much discussion and it has been described as inhibitor, substrate or neither one nor the other [16]. However, it has been proposed that the adverse effects of HQ may be due to its oxidation by tyrosinase [17], and at present its use in cosmetics has been forbidden in the European Union and the US Food and Drug Administration (FDA) has proposed a ban on all over-the-counter preparations of this compound [18]. In a similar way, in treatments with rhododendrol it has been described that the *o*-quinones produced from this compound by the action of tyrosinase, affect the redox balance of the cell [17,19]. The action of tyrosinase on hydroquinone has been characterized kinetically recently [13,20].

**Fig 1:**
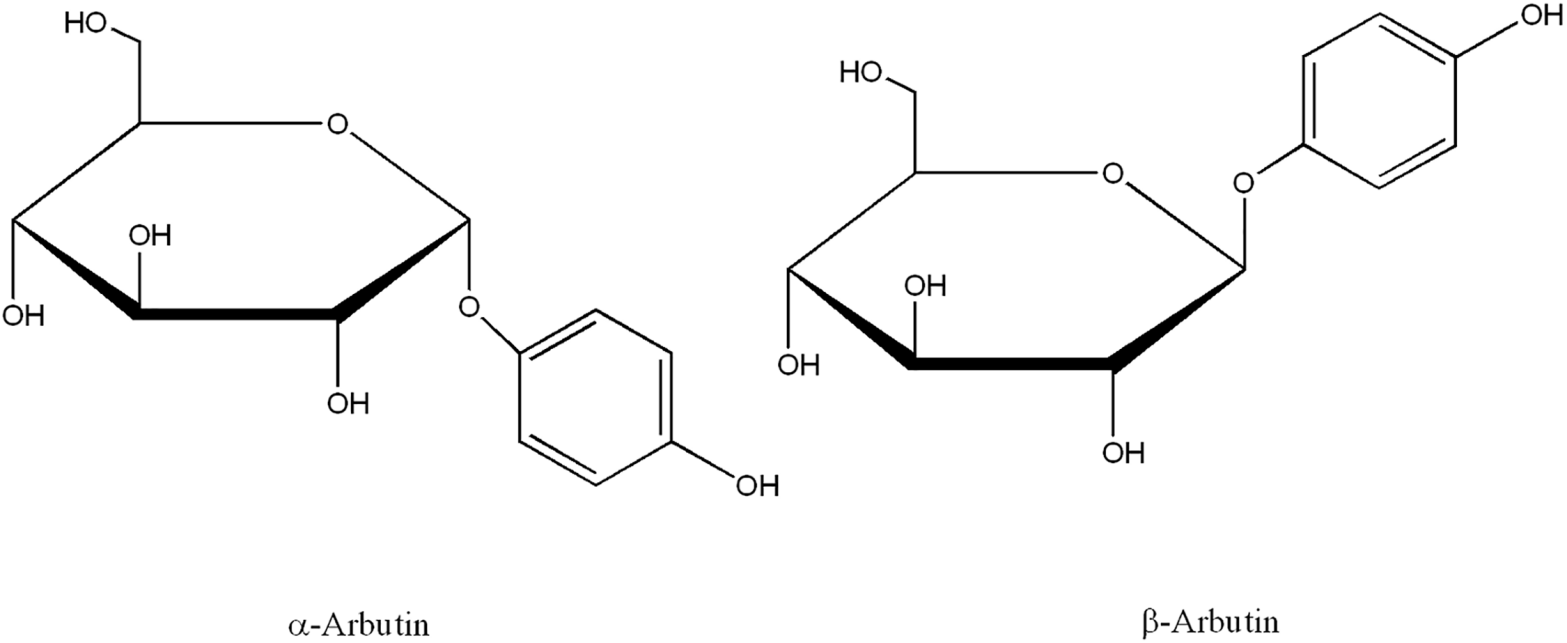
Chemical structures of α-arbutin and β-arbutin.

In order to maintain the depigmenting power of the hydroquinone, while decreasing its cytotoxicity, some derivatives of this compound such as α-arbutin (2R,3S,4S,5R,6R)-2-(hydroxymethyl)-6-(4-hydroxyphenoxy)oxane-3,4,5-triol) and β-arbutin (2R,3S,4S,5R,6S)-2-(hydroxymethyl)-6-(4-hydroxyphenoxy)oxane-3,4,5-triol), a glycosylated hydroquinones, have been used (Fig 1) [18]. β-arbutin is found in high levels in plants from the families Ericaceae and Saxifragaceae. Indeed, *Arctostaphylos uva-ursi* (Ericaceae) has been used traditionally to obtain this compound. For its part, α-arbutin is obtained principally by enzymatic synthesis from hydroquinone or β-arbutin [21,22].

The numerous studies on α and β-arbutin as inhibitors of tyrosinase are sometimes contradictory. In fact, it has been said that α-arbutin does not inhibit mushroom tyrosinase, but it is more potent than β-arbutin as an inhibitor of tyrosinase from B16 mouse melanoma [23]. On the other hand, it has been described that β-arbutin inhibits the activity of tyrosinase but not its biosynthesis in human melanocyte cultures [24]. A study on the action of arbutin on tyrosinase and the rest of the enzymes involved in the melanogenesis process showed that this compound does not change the molecular size or the content of tyrosinase, DHICA oxidase (TRP1) or dopachrome tautomerase 2 (TRP2), but that inhibition might affect the process at the post-translational level [25]. However, it has also been described that arbutin produces an increase in pigmentation in human melanocyte cultures without increasing the activity of tyrosinase [26].

Kubo et al. showed that tyrosinase can hydroxylate arbutin, generating 3,4-dihydroxphenyl-o-β-D-glucopyranoside in the presence of catalytic amounts of L-dopa [27,28]. Some studies on the activity of tyrosinase from human malignant melanoma cells demonstrate that α-arbutin is more potent than β-arbutin as an inhibitor of the enzyme [29]. Subsequently, the same authors show that α-arbutin does not inhibit the growth of cultured human melanoma cells, HMV-II, but it does inhibit melanin synthesis, meaning that the use α-arbutin in cosmetics is effective and safe for treating hyperpigmentation disorders [30]. Moreover, it was described that α and β-arbutin inhibit the formation of melanin in B16 cells induced by α-MSH and decrease the tyrosinase activity in a cell free system [31]. On the other hand, arbutin derivatives such as deoxyarbutin [32,33] or arbutin undecylenic acid ester [34] were demonstrated to be more potent than α and β-arbutin.

In addition to the applications of arbutins in cosmetics, they also have therapeutic applications such as in the treatment of infections of the urinary tract, and for their antioxidant properties, anti-inflammatory properties and antitumor activity [18].

Regarding the safety of α and β-arbutin in cosmetics, the Scientific Committee on Consumer Safety (SCCS) has stated that the limit in cosmetics should be 2% for face creams and 0.5% in body lotions in the case of α-arbutin, and 7% in face creams for β-arbutin [35,36]. Therefore, although α and β-arbutin are used in cosmetics, their action mechanism needs to be fully understood.

Recently, a study of the effect of α-arbutin on the monophenolase and diphenolase activities of tyrosinase concluded that this compound inhibits monophenolase activity and activates diphenolase activity [37]. In light of the kinetic mechanism for the monophenolase and diphenolase activities of tyrosinase proposed in the bibliography [1], this double effect led us to carry out a deeper study of α and β-arbutin.

## Materials and methods

### Materials

Mushroom tyrosinase (3130 U/mg) was obtained from Sigma (Madrid, Spain) and purified as previously described [38]. Bradford’s method was used to determine the protein content using bovine serum albumin as standard [39].

L-dopa, *tert*-butylcatechol (TBC), 3-methyl-2-benzothiazolinone hydrazone hydrochloride hydrate (MBTH), hydrogen peroxide (H_2_O_2_), α and β-arbutin were obtained from Sigma (Madrid, Spain). Stock solutions of L-dopa, TBC, and were prepared in 0.15 mM phosphoric acid to prevent auto-oxidation. Milli-Q system (Millipore Corp, Billerica, MA.) ultrapure water was used throughout.

### Determination of monophenolase and diphenolase activities

Spectrophotometric assays were carried out with a PerkinElmer Lambda-35 spectrophotometer, online interfaced with a compatible PC 486DX microcomputer controlled by UV-Winlab software, where the kinetic data were recorded, stored, and analyzed.

The diphenolase activity of tyrosinase on L-dopa [40–42] and the monophenolase activity on L-tyrosine [43] were measured at 475 nm, the maximum absorption wavelength of dopachrome. To measure the activity of tyrosinase on L-tyrosine, the quantity of *o*-diphenol necessary to reach the steady state at time t = 0 was added. In this way, the characteristic lag period of the monophenolase activity, which complicates the measurement of the *V*_0_, was eliminated. This quantity is given by the equation R = [D]_ss_ / [M]_ss_ [41,43], where [D]_ss_ and [M]_ss_ are the concentrations of *o*-diphenol and monophenol respectively in the steady state with [M]_ss_ ≈ [M]_0_. All the assays were carried out with R = 0.042.

We determined spectrophotometrically the monophenolase and diphenolase activities of tyrosinase acting on substrates that originate *o-*quinones and do not evolve with a defined stoichiometry. This was done by using MBTH [40,44], which is a potent nucleophile through its amino group which attacks enzyme-generated *o*-quinones, giving rise to an adduct. This adduct is oxidized by another molecule of *o*-quinone, leading to the accumulation of *o*-diphenol in the medium, so, the system reach the steady state. This assay method is highly sensitive, reliable, and precise [45]. MBTH traps the enzyme-generated o-quinones to render a stable MBTH-quinone adduct with a high molar absorptivity. The stability of the MBTH-quinone adducts and the rapidity of the kinetic assays makes this a suitable method for determining the monophenolase and diphenolase activities of tyrosinase [40,41,44,45] (S1A Fig). The sequence of reactions is described in S1B Fig.

All of the assays were carried out at 25°C, using 30 mM phosphate buffer at pH 7.0. Three repetitions of each experiment were made.

### Action of tyrosinase on α and β-arbutin in the presence of hydrogen peroxide

Low concentrations of tyrosinase do not show catalytic activity on α and β-arbutin, so, these compounds are described as inhibitors. However, taking into account the action mechanism of the enzyme on monophenols, the action of tyrosinase can be facilitated in the presence of an *o*-diphenol or H_2_O_2_, which transform *E*_m_ into *E*_ox_. Therefore, the possible reaction of tyrosinase on α and β-arbutin in the presence of H_2_O_2_ must be taken into account to confirm the nature of these substrates [46].

### Determination of kinetic parameters

Initial rate values (*V*_0_) were calculated at different substrate concentrations. The assays were carried out in saturating conditions of O_2_ [47–49]. The data for *V*_0_ *vs*. [arbutin]_0_ were represented and fitted to the Michaelis−Menten equation using the Sigma Plot 9.0 program for Windows [50], providing the maximum rate (*V*_max_) and the Michaelis constant (*K*_M_).

The degrees of inhibition (*i*) were calculated using tyrosinase, L-dopa, L-tyrosine and the following formula: *i* (%) = [(*V*_0_-*V*_i_)/*V*_0_] x 100, where *V*_0_ is the initial rate of the control and *V*_i_ the initial rate in the presence of the target molecule. The initial rates were obtained by linear regression fitting of the initial portions of each experimental recording.

### HPLC analysis

The high performance liquid chromatography assays were made using an Agilent 1200 Rapid Resolution coupled with a photodiode detector (UHPLC-DAD).

The samples were filtered to remove possible particles, and injected (20 μl) in a Kinetex Core Shell C-18 column (Phenomenex, Torrance) for reversed phase of 100 x 4.60 mm, 2.6 μm particle size and 100 Å pore size with a flow rate of 1 mL/min. The mobile phase was composed of water (A) and acetonitrile (B), both with formic acid 0.1%, and a multistep linear gradient: 0–30 min, 5–8% B; 30–23 min, 8–95% B; 32–35 min, 95% B. The temperature of the column was maintained at 25°C.

The chromatogram was analysed using the program Agilent ChemStation (Agilent Technologies, Madrid). The specific wavelengths used to detect the compounds were 195, 210, 280, 340, 400, 450, 475 and 500 nm [51].

### Computational docking

Molecular docking was carried out around the active site of mushroom tyrosinase with α and β-arbutin as ligands. The chemical structures for α and β-arbutin are available in the PubChem Substance and Compound database [52] through the unique chemical structure identifier CID: 158637 for α-arbutin [53], and CID: 346 for β-arbutin [54]. The molecular structure of tyrosinase was taken from the Protein Databank (PDB ID:2Y9W, Chain A) [55], corresponding to the *deoxy* form of tyrosinase from *Agaricus bisporus*. The input protein structure was prepared by adding hydrogen atoms and removing non-functional water molecules. The *met* and *oxy* forms of tyrosinase were built by a slight modification of the binuclear copper-binding site as previously described [56]. Rotatable bonds in the ligands and Gasteiger’s partial charges were assigned by AutoDockTools4 program [57,58].

The AutoDock 4.2.6 [58] package was used for docking. Lamarkian Genetic Algorithm was chosen to explore the space of active binding to search for the best conformers. The maximum number of energy evaluations was set to 2,500,000, the number of independent dockings to 200 and the population size to 150. Grid parameter files were built using AutoGrid 4.2.6 [59]. The grid box was centred close to the copper ions with a grid size set to 35x35x35 grid points (x, y and z), with grid points spacing kept at 0.375 Å. Other AutoDock parameters were used with default values. PyMOL 1.8.2.1 [60] and AutoDockTools4 [57,58] were used to edit and inspect the molecule structures and docked conformations.

## Results

### Apparent inhibitory effect of α and β-arbutin on the monophenolase and diphenolase activities of tyrosinase

**Fig 2:**
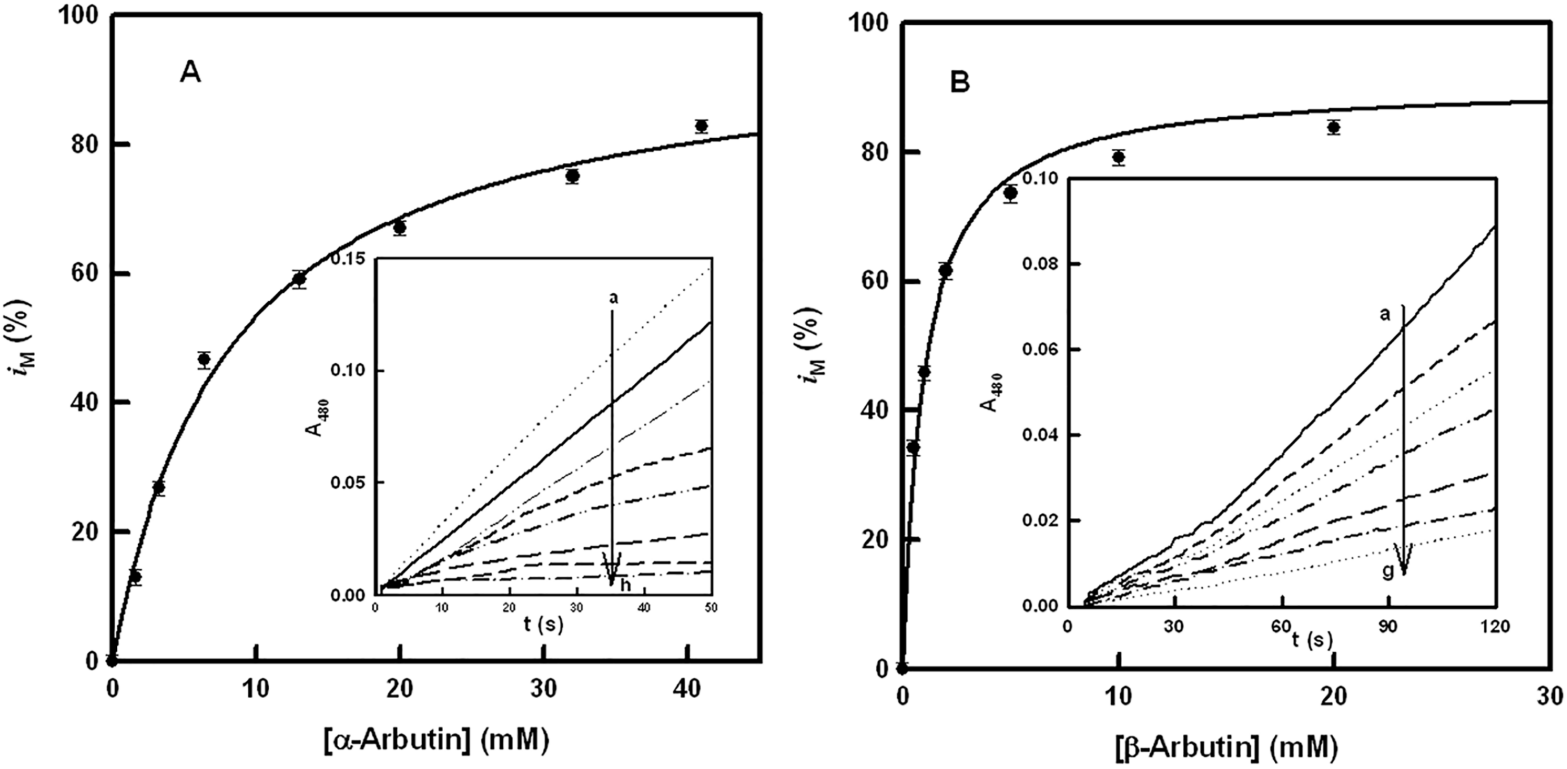
Monophenolase activity. **A.** Representation of *i*_M_ (degree of inhibition of the monophenolase activity) *vs*. the concentration of α-arbutin. The experimental conditions were [*E*]_0_ = 80 nM, [L-tyrosine]_0_ = 0.25 mM and [L-dopa]_0_ = 0.01 mM. **Inset.** Spectrophotometric recordings of the effect of different concentrations of α-arbutin on the monophenolase activity of tyrosinase, using L-tyrosine as substrate. The experimental conditions were [*E*]_0_ = 80 nM, [L-tyrosine]_0_ = 0.25 mM, [L-dopa]_0_ = 0.01 mM and α-arbutin (mM): a) 0, b) 1.5, c) 3, d) 6.5, e) 13, f) 20, g) 32 and h) 41. **B.** Representation of *i*_M_ (degree of inhibition of the monophenolase activity) *vs*. the concentration of β-arbutin. The experimental conditions were [*E*]_0_ = 80 nM, [L-tyrosine]_0_ = 0.25 mM and [L-dopa]_0_ = 0.01 mM. **Inset.** Spectrophotometric recordings of the effect of different concentrations of β-arbutin on the monophenolase activity of tyrosinase, using L-tyrosine as substrate. The experimental conditions were [*E*]_0_ = 80 nM, [L-tyrosine]_0_ = 0.25 mM, [L-dopa]_0_ = 0.01 mM and β-arbutin (mM): a) 0, b) 0.5, c) 1, d) 2, e) 5, f) 10 and g) 20.

Fig 2A and 2B show the action of α and β-arbutin on the monophenolase activity of tyrosinase using L-tyrosine as substrate. Taking into account that the assay without arbutin does not have a lag period due to the addition of L-dopa (in catalytic amounts ([D] / [M] = 0.042 [1,61]), when α-arbutin is added, the activity rate of the enzyme on L-tyrosine varies (Fig 2A Inset). When the degree of inhibition (*i*) was calculated, a hyperbole was obtained (Fig 2A). Analogous experiments with β-arbutin were made (Fig 2B Inset), obtaining similar results and a different degree of inhibition (Fig 2B). Note that big difference between the apparent inhibition values, as is shown in Table 1.

**Table 1:** Kinetic constants for the apparent inhibition of α-arbutin and β-arbutin on tyrosinase.

Compound	IC50 (mM)		$K_{I}^{\mathrm{app}}$ (mM)	
	Monophenolase	Diphenolase	Monophenolase	Diphenolase
**α-Arbutin**	8 ± 0.58	8.87 ± 0.71	2.29 ± 0.21	4 ± 0.29
**β-Arbutin**	0.9 ± 0.76	0.7 ± 0.55	1.42 ± 0.08	0.9 ± 0.05

**Fig 3:**
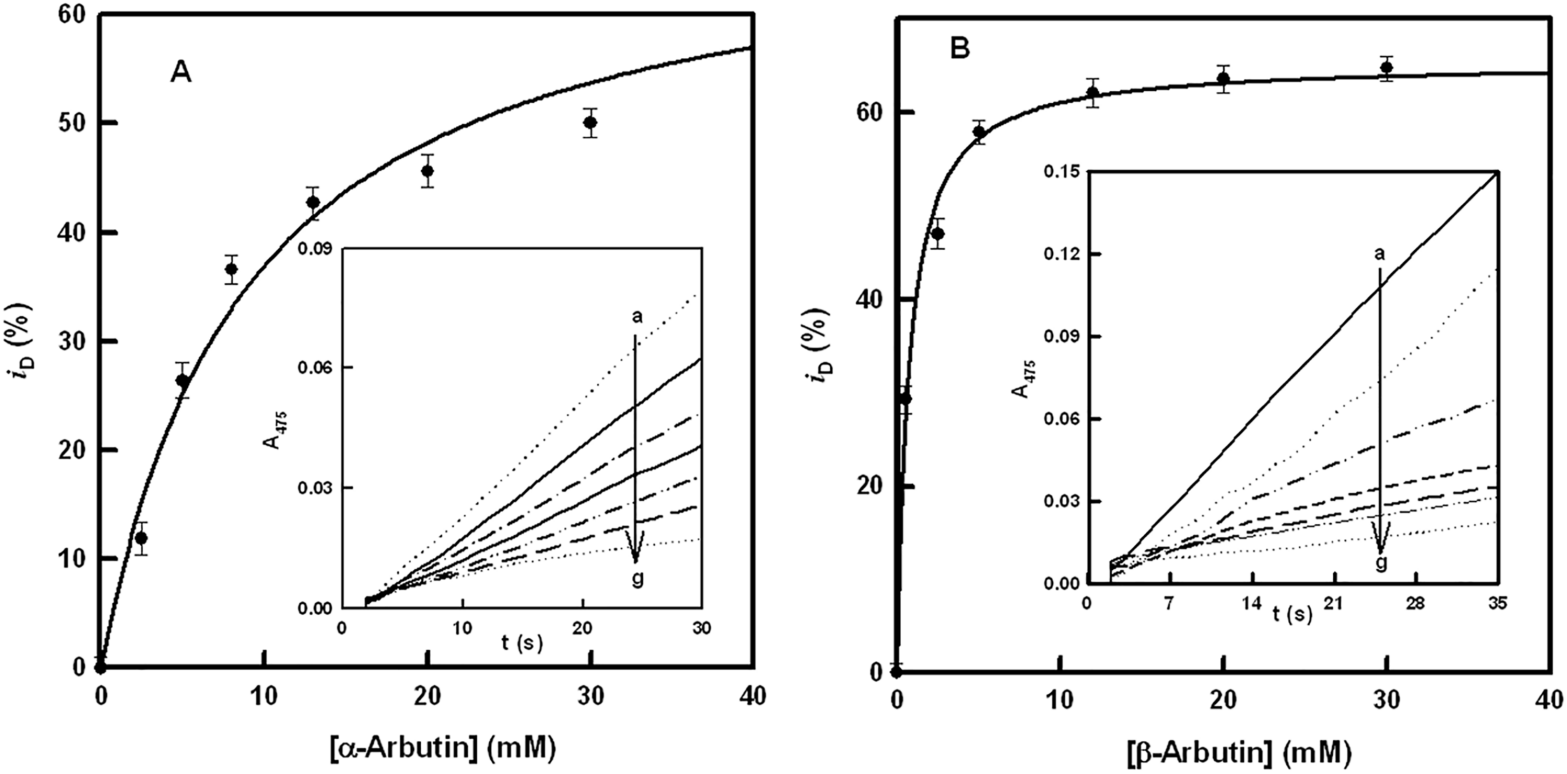
Diphenolase activity. **A.** Representation of *i*_D_ (degree of inhibition of the diphenolase activity) *vs*. the concentration of α-arbutin. The experimental conditions were [*E*]_0_ = 30 nM and [L-dopa]_0_ = 0.5 mM. **Inset.** Spectrophotometric recordings of the effect of different concentrations of α-arbutin on the diphenolase activity of tyrosinase, using L-dopa as substrate. The experimental conditions were [*E*]_0_ = 30 nM, [L-dopa]_0_ = 0.5 mM and α-arbutin (mM): a) 0, b) 2.5, c) 5, d) 8, e) 13, f) 20 and g) 30. **B.** Representation of *i*_D_ (degree of inhibition of the diphenolase activity) *vs*. the concentration of β-arbutin. The experimental conditions were [*E*]_0_ = 30 nM and [L-dopa]_0_ = 0.5 mM. **Inset.** Spectrophotometric recordings of the effect of different concentrations of β-arbutin on the diphenolase activity of tyrosinase, using L-dopa as substrate. The experimental conditions were [*E*]_0_ = 30 nM, [L-dopa]_0_ = 0.5 mM and β-arbutin (mM): a) 0, b) 0.5, c) 2.5, d) 5, e) 12, f) 20 and g) 30.

Experiments with the diphenolase activity show similar inhibition (Fig 3A Inset and 3B Inset for α and β-arbutin, respectively). The degrees of inhibition for the diphenolase activity of tyrosinase are depicted in Fig 3A and 3B, and their values are shown in Table 1, the difference between α and β-arbutin again being of note. Moreover, the degree of inhibition did not reach 100% in either case.

The fact that the degrees of inhibition for the monophenolase and diphenolase activities were not the same and that inhibition was not total in either case (α and β-arbutin) leads us propose that arbutins are probably not inhibitors, but alternative substrates [62]. Moreover, experiments with HPLC, as described in Materials and Methods, were made to confirm that there are no secondary reactions, peaks being obtained for α and β-arbutin without mixing with hydroquinone (S2 Fig).

**Fig 4:**
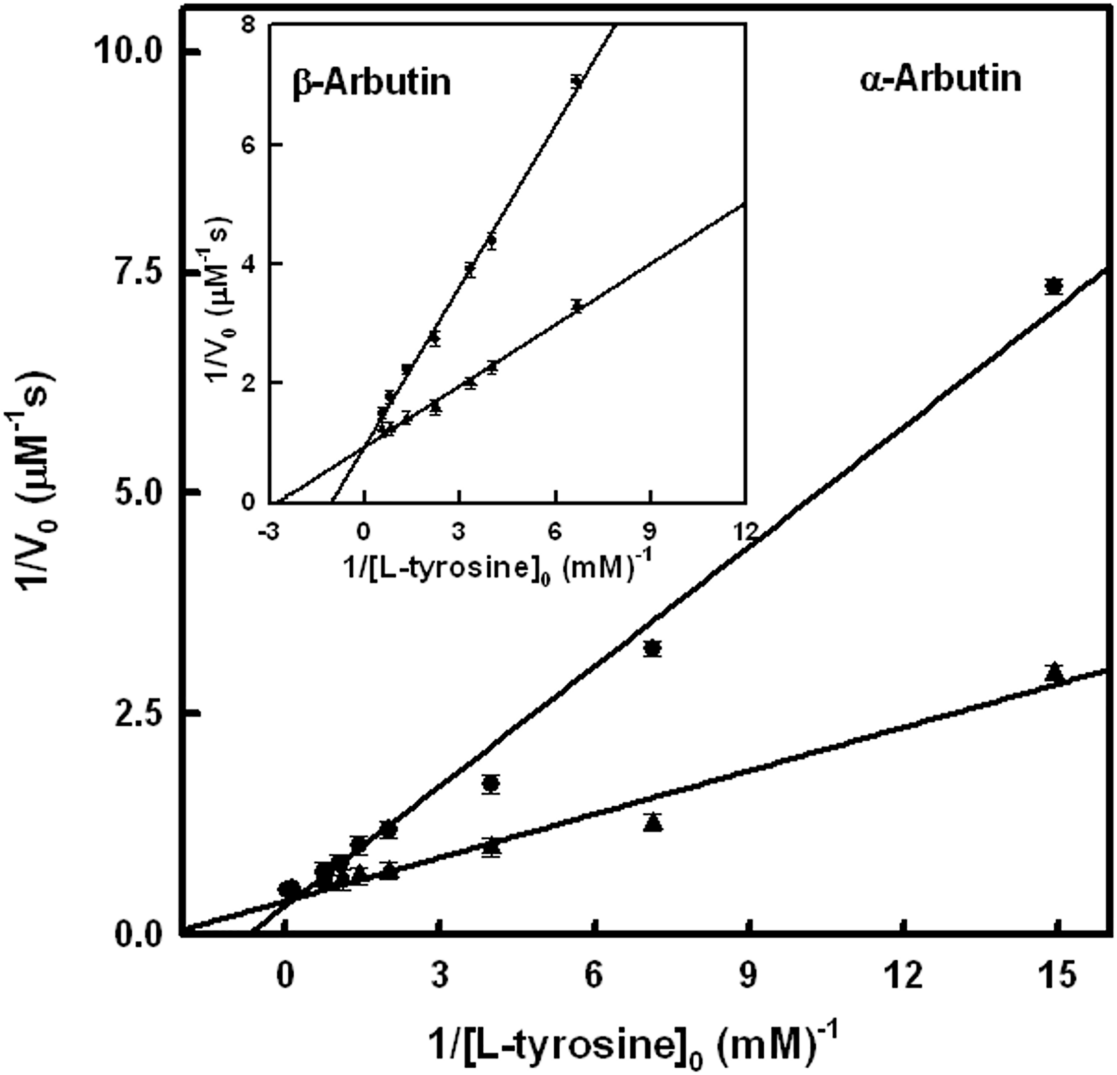
Inhibition of monophenolase activity by arbutins. Graphical representation of the Lineweaver–Burk equation to show the inhibition of the monophenolase activity of tyrosinase in the presence of 3 mM α-arbutin. The experimental conditions were [*E*]_0_ = 50 nM and R = [L-dopa]_0_ / [L-tyrosine]_0_ = 0.042. **Inset.** Graphical representation of the Lineweaver–Burk equation showing the inhibition of the monophenolase activity of tyrosinase in the presence of β-arbutin 3 mM. The experimental conditions were [*E*]_0_ = 50 nM and R = [L-dopa]_0_ / [L-tyrosine]_0_ = 0.042.

Graphical representations of the Lineweaver–Burk equation for the inhibition of the monophenolase activity by α-arbutin (Fig 4) and β-arbutin (Fig 4 Inset) are presented, showing the apparent competitive inhibition with $K_{I}^{\mathrm{app}}$ values of 2.29 ± 0.21 mM and 1.42 ± 0.08 mM, respectively. Regarding the diphenolase activity, the effect of these compounds is depicted in S3 (α-arbutin) and S3 Inset (β-arbutin) Fig, which point to an apparent competitive inhibition and $K_{I}^{\mathrm{app}}$ values of 4 ± 0.29 mM and 0.9 ± 0.05 mM, respectively. These data agree with the behaviour of an alternative substrate of tyrosinase [61,62].

### Total oxygen consumption test

**Fig 5:**
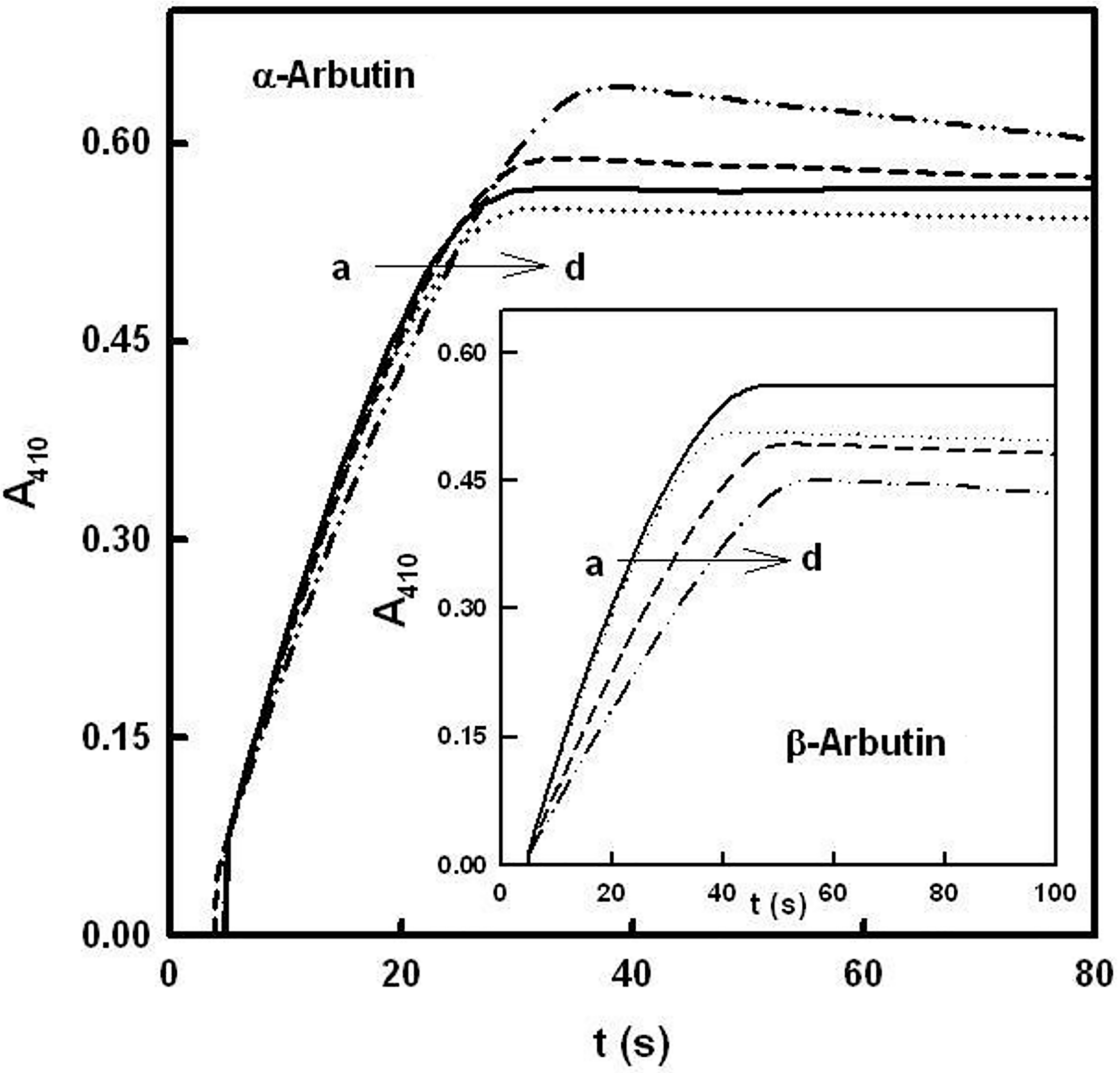
Total oxygen consumption test (TBC). A total oxygen consumption test was carried out in the presence of *tert*-butylcatechol and different concentrations of α-arbutin (mM): a) 0, b) 5, c) 10 and d) 20. The rest of the experimental conditions were [*E*]_0_ = 50 nM and [TBC]_0_ = 1 mM. **Inset.** Total oxygen consumption test in the presence of *tert*-butylcatechol and different concentrations of β- arbutin (mM): a) 0, b) 5, c) 10 and d) 20. The rest of the experimental conditions were [*E*]_0_ = 50 nM and [TBC]_0_ = 1 mM.

A total oxygen consumption test was made in order to confirm that α and β-arbutin are alternative substrates of tyrosinase. Fig 5 shows the accumulation of *o*-*tert*-butylquinone, using TBC as substrate, in the absence (recording “a”) and the presence of increasing concentrations of α-arbutin (“b-d”). The changes in absorbance and the increase in reaction time indicate that a product is originating from α-arbutin. Similar results were obtained with β-arbutin (Fig 5 Inset) as well as when the two tests were carried out using L-dopa or L-tyrosine as substrates (S4, S4 Inset, S5 and 5 Inset Figs).

### Action of tyrosinase on α and β-arbutin in the presence of hydrogen peroxide

The action of the enzyme on α and β-arbutin, respectively, in the presence of H_2_O_2_ [46] is shown in S6 and S6 Inset Fig. It can be observed that there is catalytic activity on these compounds, in the same way as happens with other alternative substrates [2,9,10].

It must be taken into account that although the activity of the enzyme is almost zero at these concentrations at short times, the addition of hydrogen peroxide transforms *E*_m_ to *E*_ox_ (S7 Fig), which is able to hydroxylate arbutin, although the *o*-quinone that is originated is unstable.

### Action of tyrosinase on α and β-arbutin at long measurement times

Spectra of the action of tyrosinase on α and β-arbutin are shown in S8 and S9 Figs, respectively, and the formation of an unstable o-quinone can be seen in each case [28]. At short times, there is barely no activity, but a self-activation of the system due to the release of *o*-diphenol occurs at long times [27,28]. S8 Inset and S9 Inset Figs demonstrate the instability of the *o*-quinones generated. Note the difference between the absorbance of both experiments from S6 Fig.

### Kinetic characterization of α and β-arbutin as substrates of tyrosinase

#### Formation and properties of the MBTH-quinone adducts

**Fig 6:**
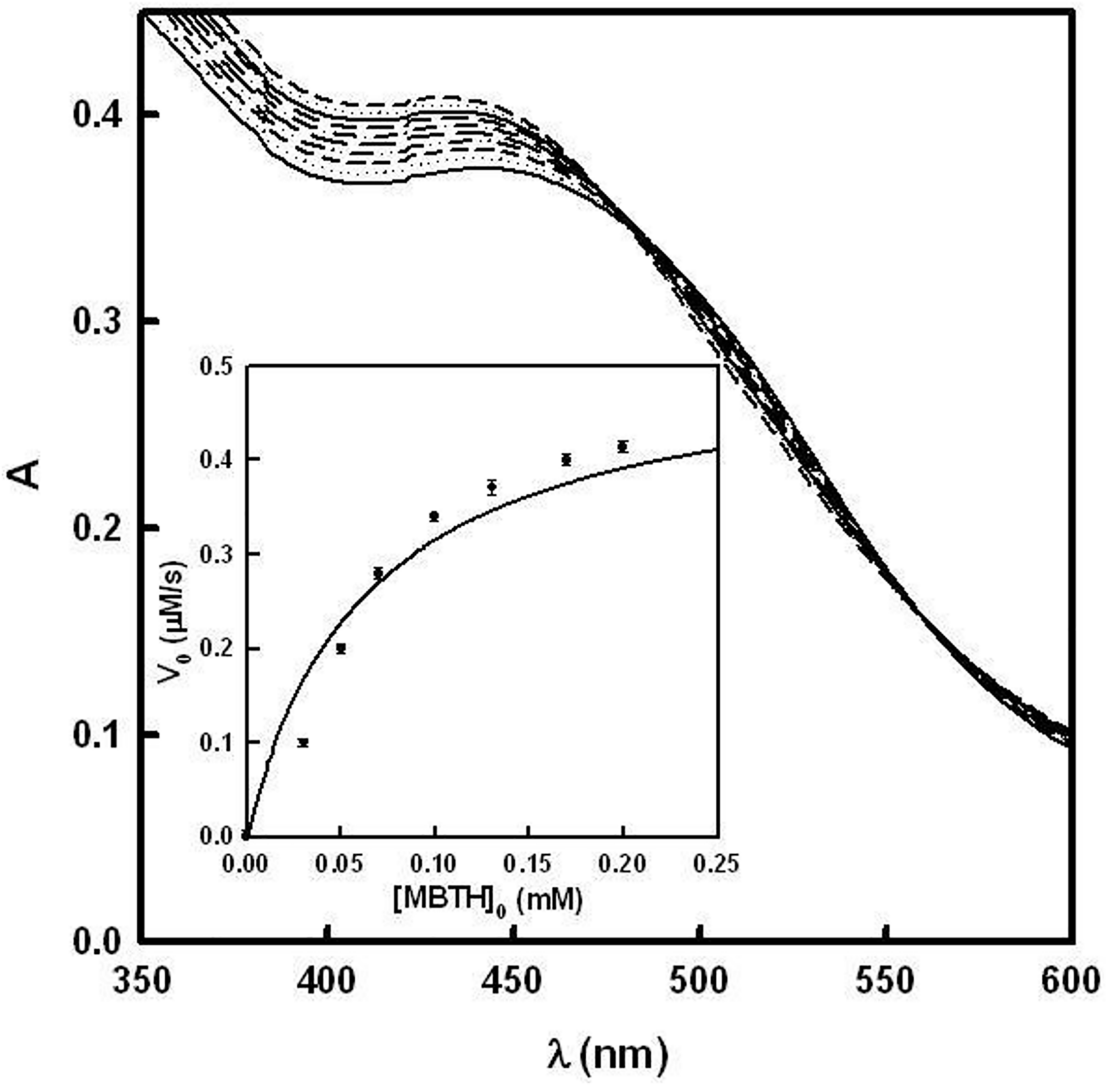
Action of tyrosinase on α-arbutin in the presence of MBTH. The experimental conditions were [*E*]_0_ = 300 nM, [MBTH]_0_ = 0.2 mM, [α-arbutin]_0_ = 10 μM and DMF 2%. The spectrophotometric recordings were made every 60 seconds. **Inset. Determination of the MBTH saturation concentration.** The experimental conditions were [*E*]_0_ = 100 nM, [α-arbutin]_0_ = 20 mM and DMF 2%.

The *o*-quinones generated by the action of tyrosinase on α and β-arbutin are unstable, as mentioned above. However, they can be attacked by a hydrazone such as MBTH, giving rise to adducts, which are oxidized to become into stable chromophores with a high molar absorptivity (S1A Fig), absorbing between 350 nm and 600 nm. This formation of adducts with MBTH has been used as method to characterize many monophenols and *o*-diphenols [40,41,44,45]. In the case of α and β-arbutin, unstable adducts are originated at pH = 7, which, as they evolve, give rise to an isosbestic point (Fig 6 and S10 Fig). These compounds were solubilised by adding 2% (v/v) DMF to the reaction medium.

MBTH is a very potent nucleophile, which, in its deprotonated form (pKa = 5.8 ± 0.4), attacks the *o*-quinone generated by the action of tyrosinase on α and β-arbutin. The saturating MBTH concentration ([MBTH]_sat_), using α and β-arbutin, is shown in Fig 6 Inset and S10 Inset Fig. This was calculated by measuring the initial rate of change in absorbance at the λ_max_ of the corresponding adduct, using different amounts of MBTH. The stoichiometry of the reaction is established from a monophenol as described in S1B Fig.

According to the stoichiometry described in S1B Fig, the rate equation for the accumulation of the cromophore with time is [43]:

$$V_{0}^{\text{A}}=\frac{V_{\max}^{\text{A}}{\left[\text{A}\right]}_{0}}{K_{\text{M}}^{\text{A}}+{\left[\text{A}\right]}_{0}}$$ (1)

where $V_{0}^{\text{A}}$ is the initial rate for the accumulation of the cromophore originated by the action of tyrosinase on arbutin, and the kinetic parameters are: $K_{\text{M}}^{\text{A}}$ = Michaelis constant for α and β-arbutin and $V_{\max}^{\text{A}}$ is the maximum rate, which is equivalent to:

$$V_{\max}^{\text{A}}=2\;k_{\text{cat}}^{\text{A}}\;{\left[E\right]}_{0}$$ (2)

#### Kinetic characterization

**Fig 7:**
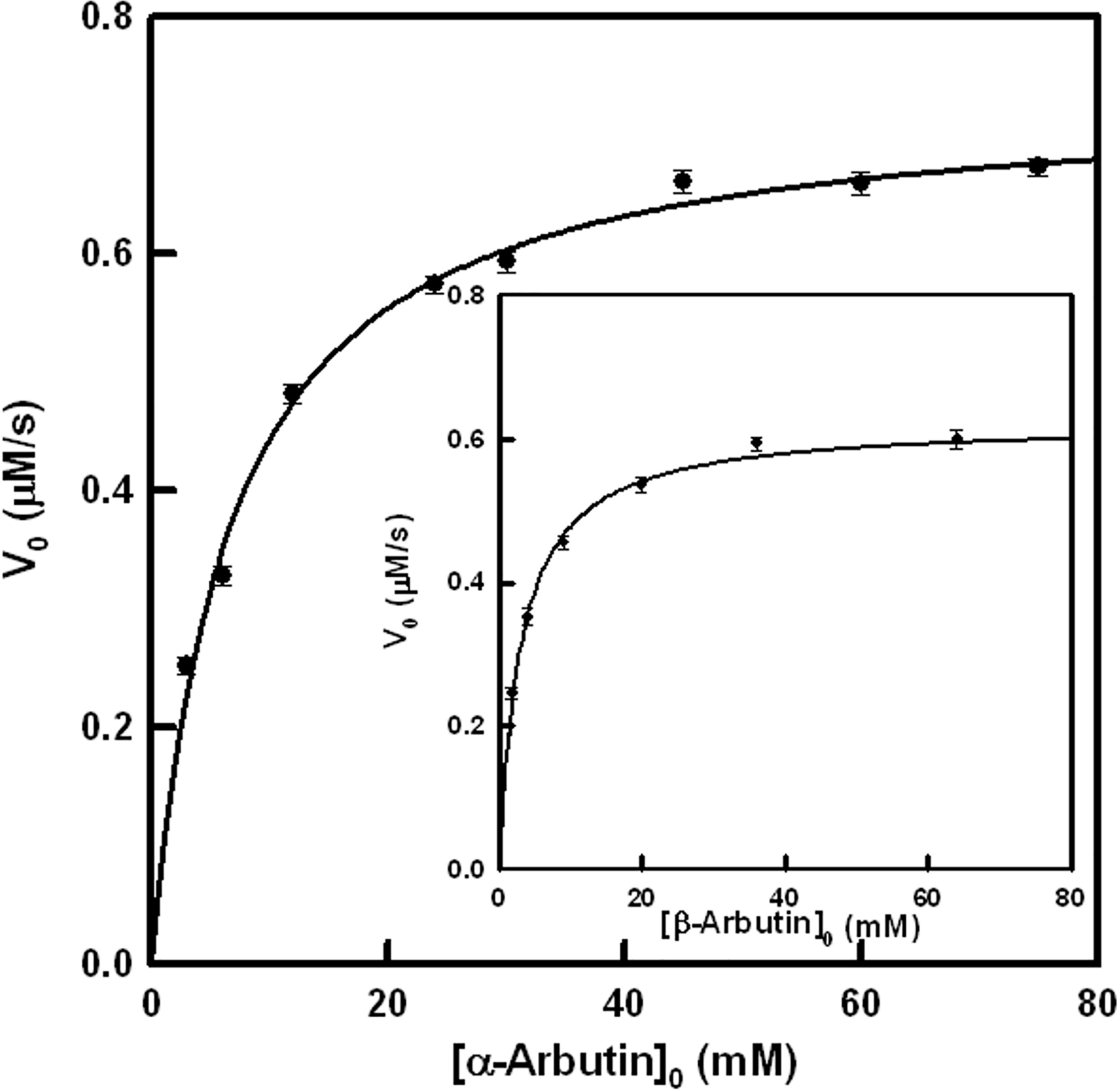
Kinetic characterization of the action of tyrosinase on arbutins. Representation of the initial rate values obtained for the action of tyrosinase on α-arbutin. The experimental conditions were [*E*]_0_ = 100 nM, [MBTH]_0_ = 0.2 mM and DMF 2%. **Inset.** Representation of the initial rate values obtained for the action of tyrosinase on β-arbutin. The experimental conditions were the same as Fig 7.

*V*_0_ values were calculated taking into account the increase of absorbance with time at λ = 480 nm for α-arbutin and λ = 490 nm for β-arbutin (isosbestic points of the respective adducts) and the molar absorptivity values, which were 22400 M^-1^ cm^-1^ and 21200 M^-1^ cm^-1^ respectively. *K*_M_ and *k*_cat_ values were obtained fitting by non-linear regression (Table 2) the initial rate values *vs*. the concentration of substrate to the Eq 1 (Fig 7 and 7 Inset).

**Table 2:** Kinetic constants for the characterization of the activity of tyrosinase on α-arbutin and β-arbutin and chemical shift values of the carbon with the phenolic hydroxyl group.

Compound	*k*_cat_ (s^-1^)	*K*_M_ (mM)	*K*_d_ (mM)	δ_4_ (ppm) [51]
**α-Arbutin**	4.43 ± 0.33	6.5 ± 0.58	4.4	151.71
**β-Arbutin**	3.77 ± 0.29	3 ± 0.19	2.4	153.14

### Molecular docking

**Fig 8:**
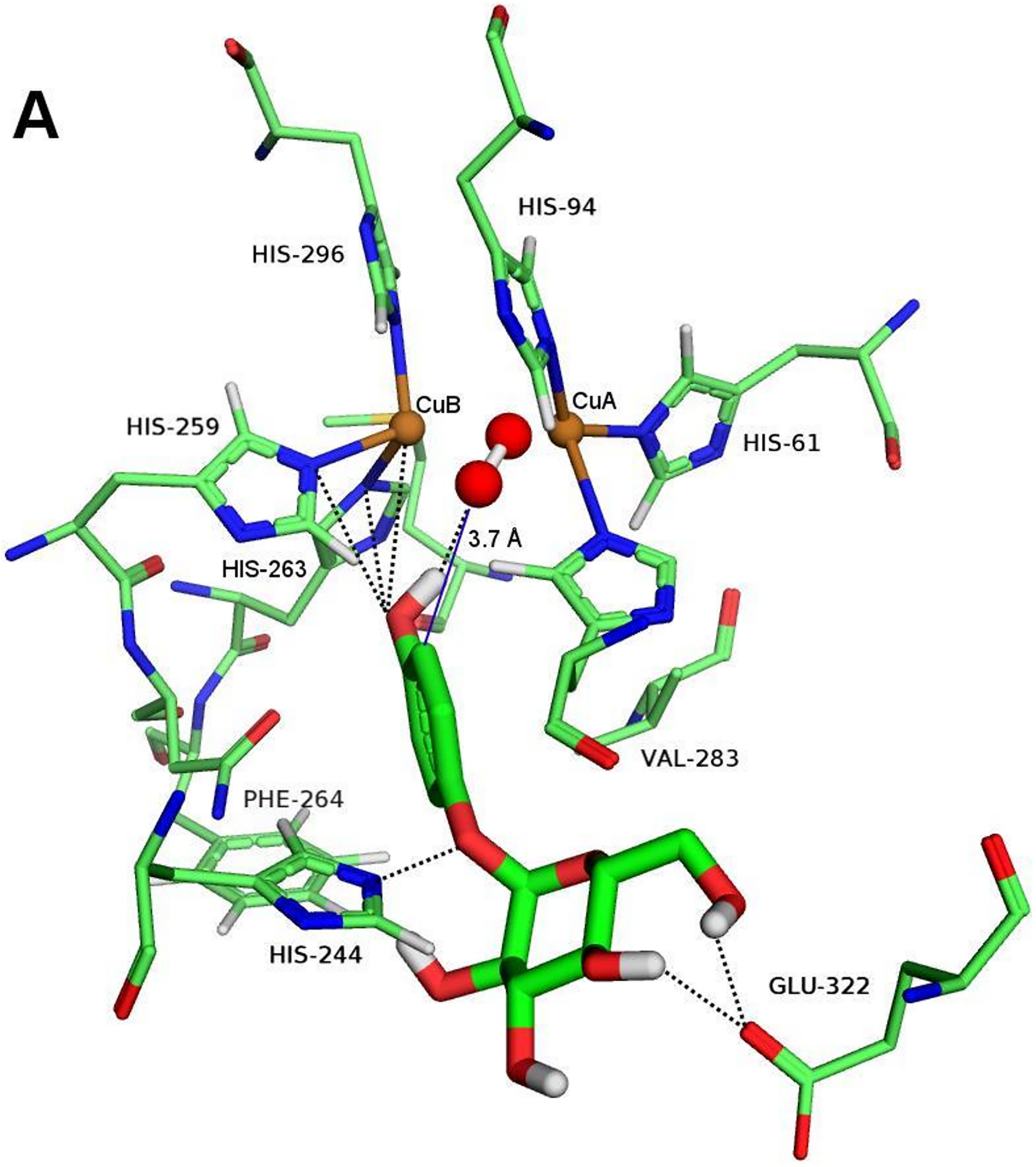
Computational docking of α-arbutin. Docking poses obtained with AutoDock of α-arbutin in the active site of the oxy form of mushroom tyrosinase are shown as sticks. The atom colors are as follows: red = oxygen, blue = nitrogen, brown = copper, green = carbon, and white = hydrogen. Polar interactions and hydrogen bonds are shown as black dotted lines. The distance from the *ortho* carbon of the phenolic ring to the oxygen atom of the peroxide ion is shown in blue lines.

Docking complexes between the oxy form of mushroom tyrosinase and α and β-arbutin at the binuclear copper active site of tyrosinase were analyzed. Fig 8 and S11 Fig show the docking poses corresponding to the lowest binding energies at the active site of tyrosinase where catalysis can take place, as mentioned above.

It is interesting that the phenolic groups of α-arbutin (Fig 8) and β-arbutin (S11 Fig) show similar interactions at the catalytic site of tyrosinase. The hydroxyl group could establish hydrogen bonds with the peroxide ion and polar contacts with a copper ion as well as with H259 and H263. However, the aromatic ring position cannot be stabilized by π-π-interactions with H263 as occurs in many other aromatic ligands [10,11,63]. The *ortho* carbon is found 3.7 Å from an oxygen atom of the peroxide ion, close enough to allow substrate hydroxylation by the monophenolase activity.

Conversely, a clear difference can be seen in the glycosyl moiety orientation of both ligands (Fig 8 and S11 Fig), which form hydrogen bonds with E322 from hydroxyl groups of C4 and C6 of the glucopyranose ring. Moreover, in both of them, there is a polar interaction of the glycosidic oxygen atom of the glucopyranose ring with H244. Nevertheless, only β-arbutin produces an additional polar interaction between the ether oxygen atom of the glucopyranose ring and the same histidine residue.

Docking of β-arbutin to mushroom tyrosinase has previously been reported to occur at the active site, where it interacts with E256 and N260. The discrepancy with our results is due to the different tyrosinase form used by the authors [6]. They used the *met* form of tyrosinase in contrast with this work where the *oxy* form was selected for docking purposes since the monophenolase activity resides in this form. The presence of the peroxide ion in the binuclear copper centre requires a different arrangement of the substrate [10].

The dissociation constants, *K*_d_, calculated for the docking conformations shown in Fig 8 and S11 Fig are 4.4 mM and 2.4 mM for α and β-arbutin, respectively. The higher affinity of β-arbutin could be explained by the additional conformational stability of the glucopyranose ring provided by three anchor points to the protein compared with two in α-arbutin. These *K*_d_ values are in good agreement with the reported IC50^app^ and $K_{i}^{\text{app}}$ values for arbutin ranging from 0.37 mM to 8.4 mM [6,23,64–66], acting as inhibitor of different substrates. Moreover, when these compounds are studied as substrates of the enzyme, the *K*_d_ values obtained by docking studies are in the same range as the *K*_M_ values (Table 2).

## Discussion

The glycosylated derivatives of hydroquinone α and β-arbutin are used as depigmenting agents and their concentrations are regulated by law. In this work, the possible behaviour of these compounds as substrate of tyrosinase is studied.

The experiments described in Figs 2 and 3 demonstrate that α and β-arbutin always act as apparent inhibitors of tyrosinase on the monophenolase and diphenolase activities. The results of these experiments do not agree with those described in the literature, which propose that α-arbutin only inhibits the monophenolase activity and activates the diphenolase activity [37]. Such results could be explained if the α-arbutin is partially hydrolyzed and the hydroquinone acts as activator of the diphenolase activity of the enzyme, as, indeed, has been described recently [67]. However, experiments with HPLC show that the samples of α and β-arbutin were not hydrolyzed, so, there is no possibility that this kind of activation occurred in our case (S2 Fig). Furthermore, the IC50 values for monophenolase (Fig 2) and diphenolase (Fig 3) activities were not the same, and neither were the apparent inhibition constants (Fig 4 and S3 Fig), as can be seen in Table 1. These observations suggest that the compounds are alternative substrates of the enzyme. This was lent weight by the oxygen consumption test with TBC (Fig 5 and 5 Inset), L-dopa (S4 and S4 Inset Fig) or L-tyrosine (S5 and S5 Inset Fig). Similar results were obtained with H_2_O_2_ (S6 and S6 Inset Fig) since to this compound converts the *met* form of tyrosinase into the *oxy* form, which is able to act on α and β-arbutin.

The following mechanisms are proposed to explain the action of α and β-arbutin on the monophenolase and diphenolase activities of tyrosinase (S12 and S13 Figs, respectively), based on the above results. The schemes show how α and β-arbutin act as competitive substrates and alternatives to L-tyrosine and L-dopa.

The kinetic analysis of the mechanism is shown in Supporting Information. Despite the complexity of the mechanisms, an equation for the formation rate of dopachrome can be obtained. This equation agrees with the characteristics of the action of a competitive inhibitor: *V*_max_ does not vary when the concentration of substrate, L-dopa or L-tyrosine, saturates the enzyme. S7 and S12 equations of Supporting Information demonstrate that the same maximum rate is obtained when the concentration of L-tyrosine or L-dopa increases and the concentration of the apparent inhibitor (α and β-arbutin) remains stable (Fig 4 and S3 Fig).

The experiments shown in Figs 5, 5 Inset, S4, S4 Inset, S5 and S5 Inset demonstrate that α and β-arbutin are alternative substrates of tyrosinase and, so, enzymatic activity is originated in the presence of hydrogen peroxide, since this compound gives rise to the formation of oxytyrosinase (S6 and S7 Figs).

Tyrosinase hydroxylates monophenols to *o*-diphenols through the action of *E*_ox_ on A, originating the *E*_ox_A complex, which becomes *E*_m_AOH, which, in turn, can be oxidized giving rise to *E*_d_ and *o*-quinone (P) or *E*_m_ and *o*-diphenol (AOH). When the initial concentration of enzyme is high, there is sufficient *E*_ox_ to generate the *o*-diphenol of the arbutins, which is consumed with time and, so, the decay rate of these *o*-quinones is greater than the rate of formation and the process stops. In this way, catalytic amounts of released *o*-diphenol are able to activate the system for long period of time, as can be seen in S8 and S9 Figs, where α and β-arbutin, respectively, are consumed by tyrosinase. Note that the respective insets show the instability of the *o*-quinones generated, again demonstrating that they behave as substrates of the enzyme, as demonstrated previously in the presence of catalytic amounts of L-dopa [28].

The measurements of the initial rate in the isosbestic point (corresponding to the adduct originated by the reaction of α and β-arbutin with MBTH (Fig 6 Inset and S10 Inset Fig)) were fitted by non-linear regression to Eq 1, thus obtaining the $K_{\text{M}}^{\text{A}}$ and $k_{\text{cat}}^{\text{A}}$ values for these substrates (Table 2).

The distance between the oxygen of the peroxide group and the carbon with the hydroxyl group in *ortho* position facilitates hydroxylation. Both values are almost the same, although α-arbutin has a slightly higher *δ*_4_ value (Table 2) [68]. β-arbutin has a lower Michaelis constant than α-arbutin (Table 2). The docking results agree with these values.

The docking results agree quite well with our experimental values for the *K*_M_ values (Table 2): β-arbutin exhibits higher binding affinity than α-arbutin. However, both ligands are hydroxylated at essentially the same velocity, since the catalytic rate constants, *k*_cat_, were found to be similar (Table 2). This result also agrees with a similar arrangement of the phenolic group at the binuclear copper centre and with the distance from the *ortho* carbon to the peroxide ion.

In conclusion, this work demonstrate that α and β-arbutin act as substrates of tyrosinase, since the enzyme is able to hydroxylate them and, subsequently, to oxidize the originated *o*-diphenol, as well as the hydroquinone, whose quinones are cytotoxic especially when they act on thiol compounds in the melanosome. Such possible adverse effects of α and β-arbutin should be studied in the future.

## Supporting information

S1 Fig **A. Schematic representation of the mechanism proposed to explain the oxidation of α and β-arbutin by tyrosinase in the presence of MBTH.** A = α or β-arbutin, D = *o*-diphenol, Q = *o*-quinone, N = MBTH, ND = MBTH-adduct, NQ = MBTH-A-*o*-quinone adduct. **B. Stoichiometry of the sequence of reactions that lead to the formation of MBTH-*o*-quinone adduct.**
(TIF)

S2 Fig **Chromatogram of the A) α-arbutin 1 mM, B) β-arbutin 1 mM and C) hydroquinone 1 mM.** The retention times were 2.58, 2.61 and 3.64 min respectively. Conditions are described in Materials and Methods.
(TIF)

S3 Fig Inhibition of diphenolase activity by arbutins. Graphical representation of the Lineweaver–Burk equation showing the inhibition of the diphenolase activity of tyrosinase in the presence of β-arbutin 3 mM. The experimental conditions were [*E*]_0_ = 30 nM. **Inset.** Graphical representation of the Lineweaver–Burk equation showing the inhibition of the diphenolase activity of tyrosinase in the presence of β-arbutin 3 mM. The experimental conditions were [*E*]_0_ = 30 nM.
(TIF)

S4 Fig Total oxygen consumption test (L-dopa). Total oxygen consumption test in the presence of L-dopa and different concentrations of α-arbutin (mM): a) 0, b) 2, c) 5 and d) 20. The rest of the experimental conditions were [*E*]_0_ = 80 nM and [L-dopa]_0_ = 0.5 mM. **Inset.** Total oxygen consumption test in the presence of L-dopa and different concentrations of β-arbutin (mM): a) 0, b) 2, c) 5 and d) 20. The rest of the experimental conditions were [*E*]_0_ = 80 nM and [L-dopa]_0_ = 0.5 mM.
(TIF)

S5 Fig Total oxygen consumption test (L-tyrosine). Total oxygen consumption test in the presence of L-tyrosine and different concentrations of α-arbutin (mM): a) 0, b) 1, c) 2 and d) 4. The rest of the experimental conditions were [*E*]_0_ = 100 nM, [L-tyrosine]_0_ = 1 mM and [L-dopa]_0_ = 0.042 mM. **Inset.** Total oxygen consumption test in the presence of L-tyrosine and different concentrations of β-arbutin (mM): a) 0, b) 1, c) 2 and d) 4. The rest of the experimental conditions were [*E*]_0_ = 100 nM, [L-tyrosine]_0_ = 1 mM and [L-dopa]_0_ = 0.042 mM.
(TIF)

S6 Fig Action of tyrosinase on arbutins in the presence of hydrogen peroxide. Action on α-arbutin. The experimental conditions were [*E*]_0_ = 300 nM, [H_2_O_2_]_0_ = 10 mM and [α-arbutin]_0_ = 0.5 mM. The spectrophotometric recordings were also made every 60 seconds. **Inset.** Action on β-arbutin. The experimental conditions were [*E*]_0_ = 300 nM, [H_2_O_2_]_0_ = 10 mM and [β-arbutin]_0_ = 0.5 mM. The spectrophotometric recordings were made every 60 seconds.
(TIF)

S7 Fig Schematic representation of the action mechanism of tyrosinase on monophenols in the absence and the presence of hydrogen peroxide [46]. D = *o*-diphenol (L-dopa), Q = *o*-dopaquinone, A = arbutin, Cr = dopachrome, *E*_m_ = metatyrosinase, *E*_d_ = deoxytyrosinase and *E*_ox_ = oxytyrosinase.
(TIF)

S8 Fig Action of tyrosinase on α-arbutin at long times. The experimental conditions were [*E*]_0_ = 50 nM and [α-arbutin]_0_ = 1 mM. The spectrophotometric recordings were made every 2 minutes. **Inset. Instability of the *o*-quinone produced by the action of tyrosinase on α-arbutin.** Recording of the formation time of the *o*-quinone and its fast decay. The experimental conditions were the same as S8 Fig.
(TIF)

S9 Fig Action of tyrosinase on β-arbutin at long times. The experimental conditions were [*E*]_0_ = 50 nM and [β-arbutin]_0_ = 1 mM. The spectrophotometric recordings were made every 2 minutes. **Inset. Instability of the *o*-quinone produced by the action of tyrosinase on β-arbutin.** Recording of the formation time of the *o*-quinone and its fast decay. The experimental conditions were the same as S9 Fig.
(TIF)

S10 Fig Action of tyrosinase on β-arbutin in the presence of MBTH. The experimental conditions were [*E*]_0_ = 300 nM, [MBTH]_0_ = 0.2 mM, [β-arbutin]_0_ = 10 μM and DMF 2%. The spectrophotometric recordings were made every 60 seconds. **Inset. Determination of the MBTH saturation concentration.** The experimental conditions were [*E*]_0_ = 100 nM, [β-arbutin]_0_ = 20 mM and DMF 2%.
(TIF)

S11 Fig Computational docking of β-arbutin. Docking poses obtained with AutoDock of β-arbutin in the active site of the oxy form of mushroom tyrosinase are shown as sticks. The color scheme is as described in Fig 8.
(TIF)

S12 Fig Schematic representation of the kinetic mechanism for the action of tyrosinase on L-tyrosine in the presence of α or β-arbutin. M = monophenol (L-tyrosine), D = *o*-diphenol (L-dopa), Q = *o*-dopaquinone, P = *o*-quinone from α or β-arbutin, AOH = *ortho*-hydroxylated arbutin, Cr = dopachrome, *E*_m_ = metatyrosinase, *E*_d_ = deoxytyrosinase and *E*_ox_ = oxytyrosinase.
(TIF)

S13 Fig Schematic representation of the kinetic mechanism for the action of tyrosinase on L-dopa in the presence of α or β-arbutin. D = *o*-diphenol (L-dopa), A = Arbutin, Q = *o*-dopaquinone, P = *o*-quinone from α or β-arbutin, AOH = *ortho*-hydroxylated arbutin, Cr = dopachrome, *E*_m_ = metatyrosinase, *E*_d_ = deoxytyrosinase and *E*_ox_ = oxytyrosinase.
(TIF)

S1 File Kinetic analysis. (DOCX)
