# Supplementary figures and images for: Action of tyrosinase on alpha and beta-arbutin: A kinetic study
Source: PLoS One. 2017 May 11;12(5):e0177330. doi: 10.1371/journal.pone.0177330 (PMC5426667; doi:10.1371/journal.pone.0177330)

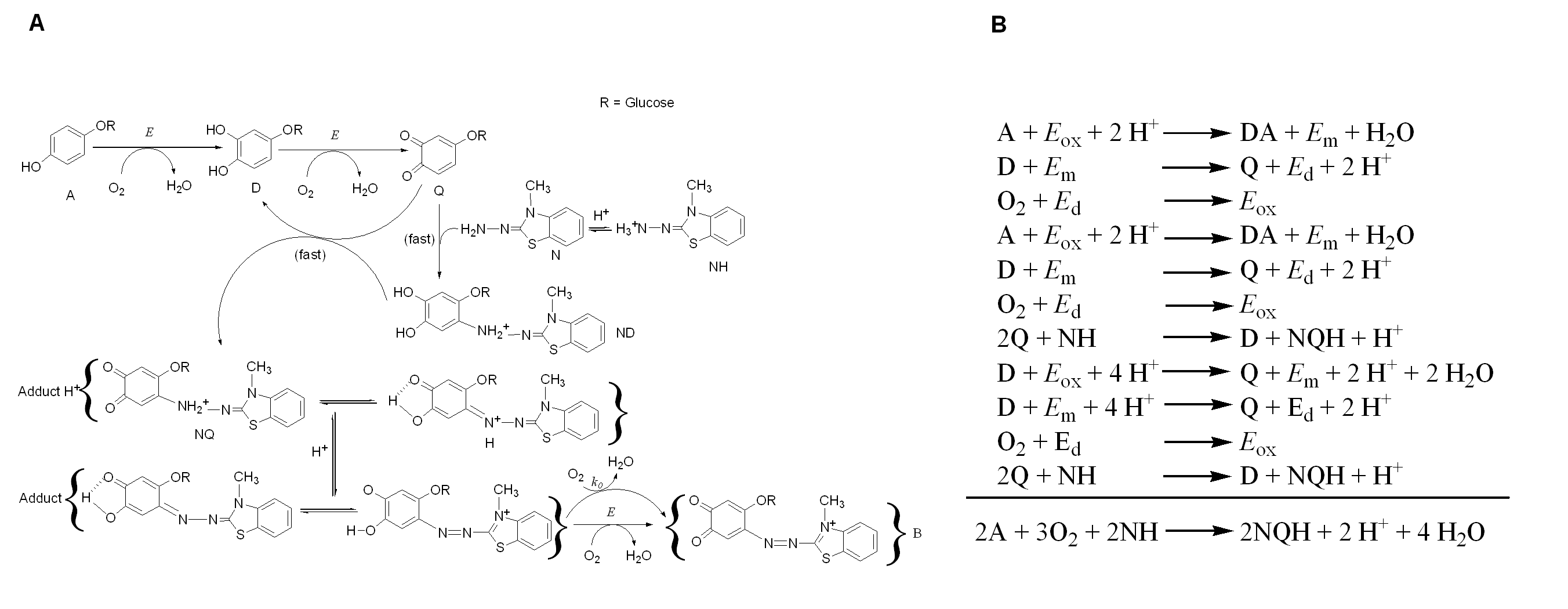

Supplement: S1 Fig — A. Schematic representation of the mechanism proposed to explain the oxidation of α and β-arbutin by tyrosinase in the presence of MBTH. A = α or β-arbutin, D = o-diphenol, Q = o-quinone, N = MBTH, ND = MBTH-adduct, NQ = MBTH-A-o-quinone adduct. B. Stoichiometry of the sequence of reactions that lead to the formation of MBTH-o-quinone adduct. (TIF) [file pone.0177330.s001.TIF]

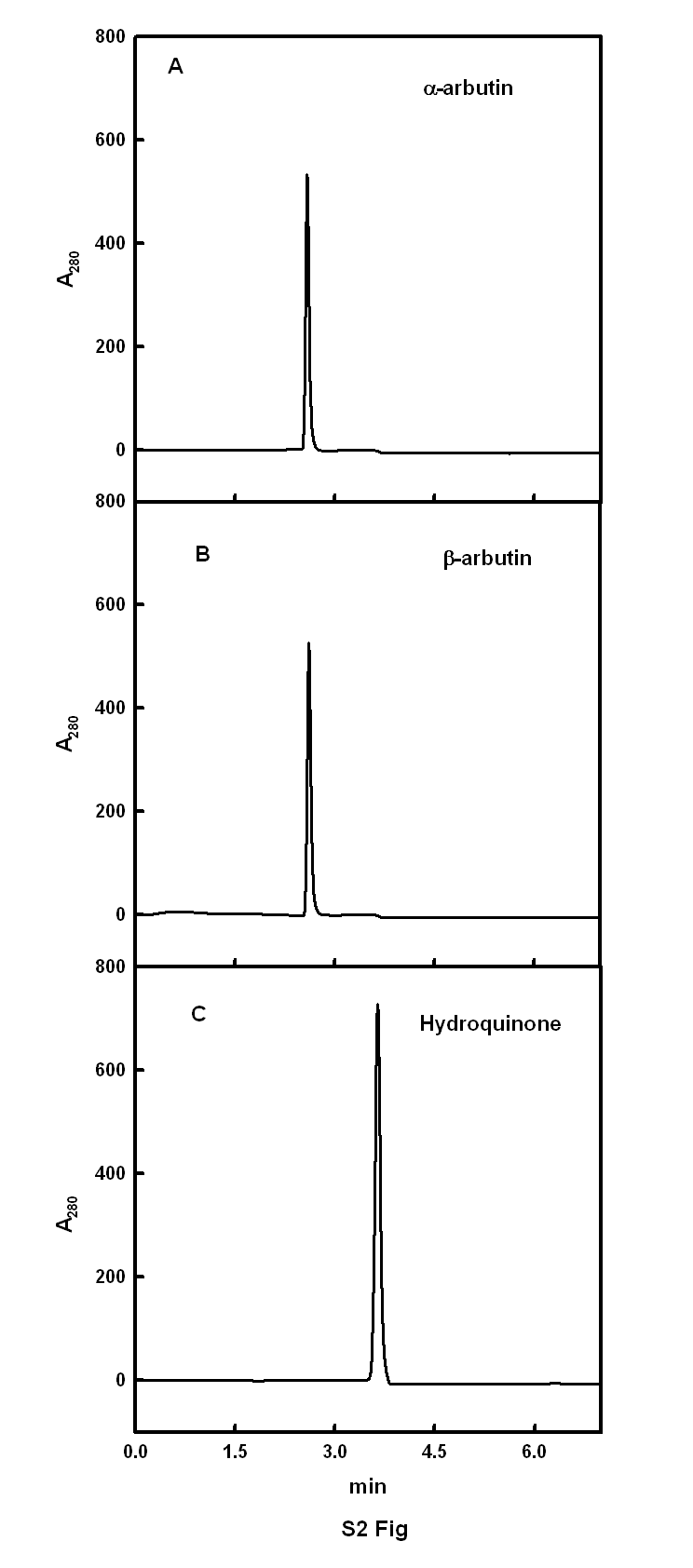

Supplement: S2 Fig — Chromatogram of the A) α-arbutin 1 mM, B) β-arbutin 1 mM and C) hydroquinone 1 mM. The retention times were 2.58, 2.61 and 3.64 min respectively. Conditions are described in Materials and Methods. (TIF) [file pone.0177330.s002.TIF]

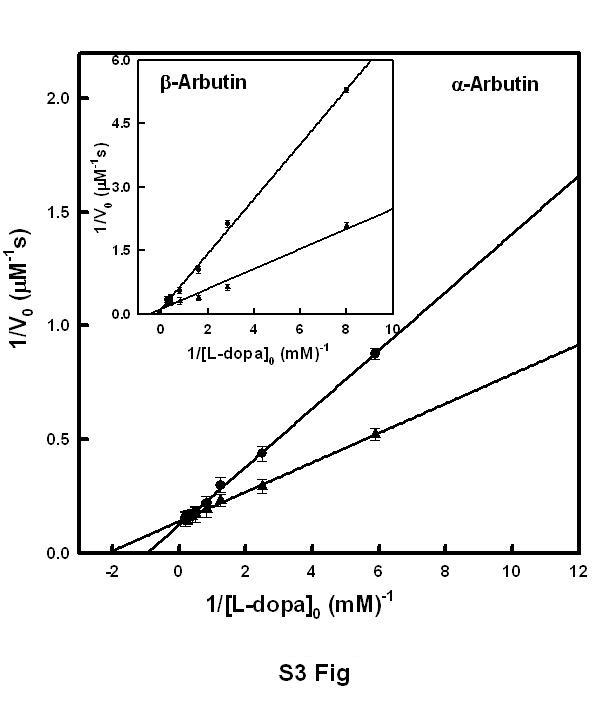

Supplement: S3 Fig — Graphical representation of the Lineweaver–Burk equation showing the inhibition of the diphenolase activity of tyrosinase in the presence of β-arbutin 3 mM. The experimental conditions were [E]0 = 30 nM. Inset. Graphical representation of the Lineweaver–Burk equation showing the inhibition of the diphenolase activity of tyrosinase in the presence of β-arbutin 3 mM. The experimental conditions were [E]0 = 30 nM. (TIF) [file pone.0177330.s003.TIF]

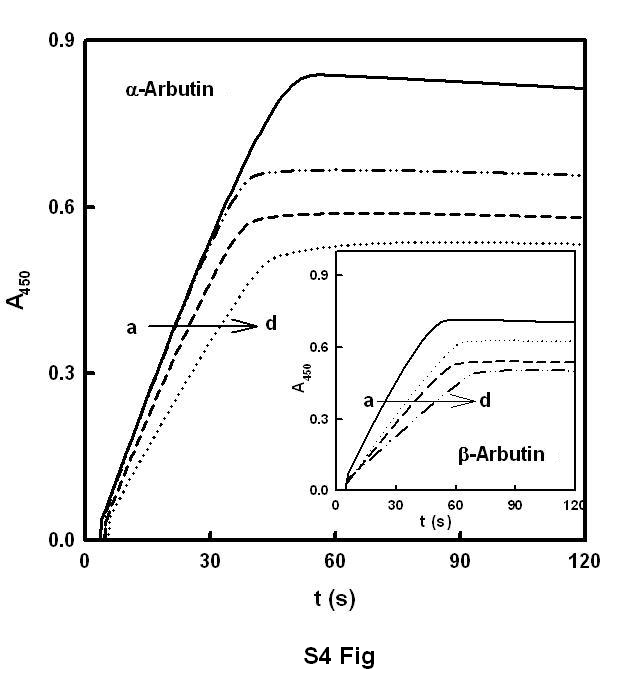

Supplement: S4 Fig — Total oxygen consumption test in the presence of L-dopa and different concentrations of α-arbutin (mM): a) 0, b) 2, c) 5 and d) 20. The rest of the experimental conditions were [E]0 = 80 nM and [L-dopa]0 = 0.5 mM. Inset. Total oxygen consumption test in the presence of L-dopa and different concentrations of β-arbutin (mM): a) 0, b) 2, c) 5 and d) 20. The rest of the experimental conditions were [E]0 = 80 nM and [L-dopa]0 = 0.5 mM. (TIF) [file pone.0177330.s004.TIF]

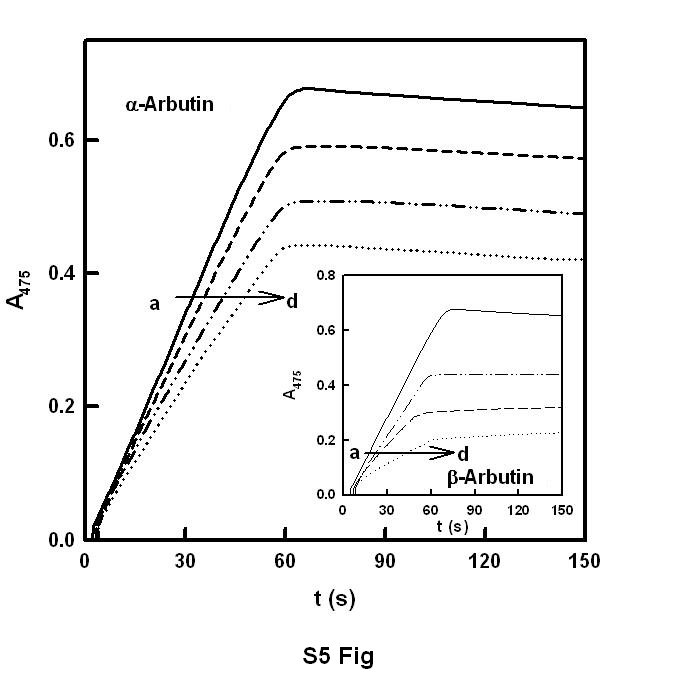

Supplement: S5 Fig — Total oxygen consumption test in the presence of L-tyrosine and different concentrations of α-arbutin (mM): a) 0, b) 1, c) 2 and d) 4. The rest of the experimental conditions were [E]0 = 100 nM, [L-tyrosine]0 = 1 mM and [L-dopa]0 = 0.042 mM. Inset. Total oxygen consumption test in the presence of L-tyrosine and different concentrations of β-arbutin (mM): a) 0, b) 1, c) 2 and d) 4. The rest of the experimental conditions were [E]0 = 100 nM, [L-tyrosine]0 = 1 mM and [L-dopa]0 = 0.042 mM. (TIF) [file pone.0177330.s005.TIF]

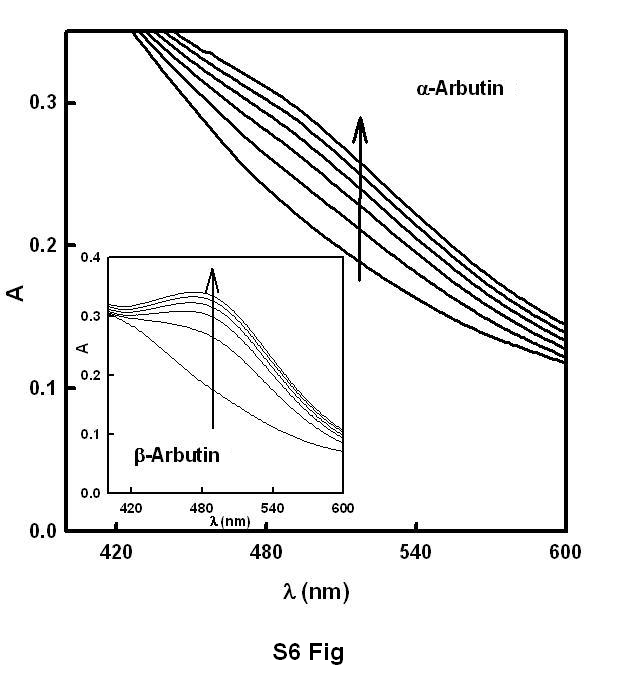

Supplement: S6 Fig — Action on α-arbutin. The experimental conditions were [E]0 = 300 nM, [H2O2]0 = 10 mM and [α-arbutin]0 = 0.5 mM. The spectrophotometric recordings were also made every 60 seconds. Inset. Action on β-arbutin. The experimental conditions were [E]0 = 300 nM, [H2O2]0 = 10 mM and [β-arbutin]0 = 0.5 mM. The spectrophotometric recordings were made every 60 seconds. (TIF) [file pone.0177330.s006.TIF]

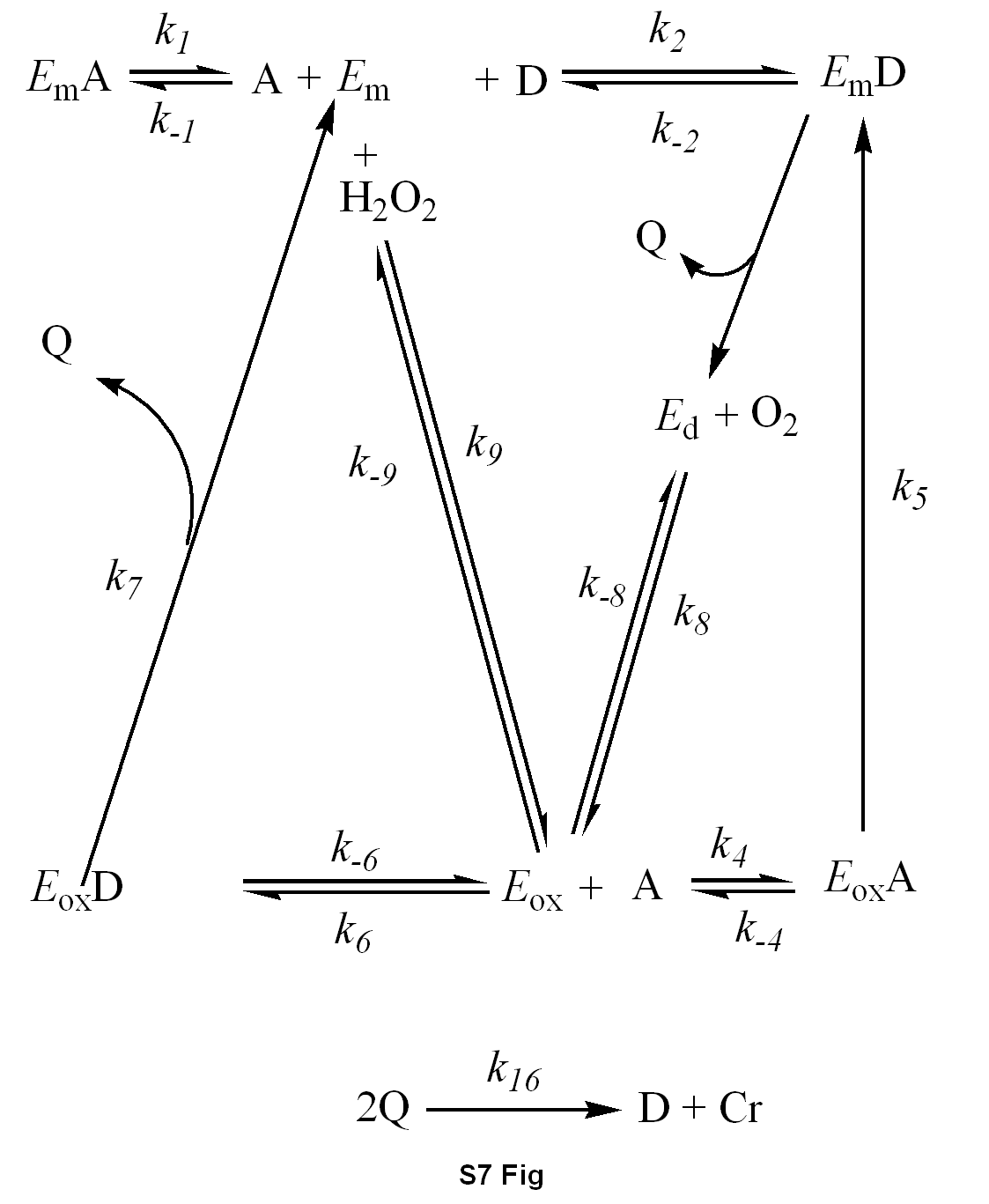

Supplement: S7 Fig — D = o-diphenol (L-dopa), Q = o-dopaquinone, A = arbutin, Cr = dopachrome, Em = metatyrosinase, Ed = deoxytyrosinase and Eox = oxytyrosinase. (TIF) [file pone.0177330.s007.tif]

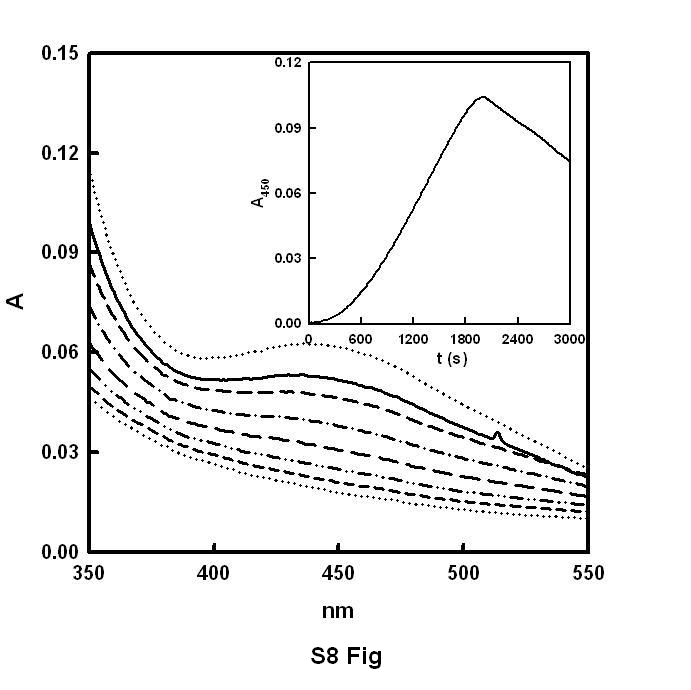

Supplement: S8 Fig — The experimental conditions were [E]0 = 50 nM and [α-arbutin]0 = 1 mM. The spectrophotometric recordings were made every 2 minutes. Inset. Instability of the o-quinone produced by the action of tyrosinase on α-arbutin. Recording of the formation time of the o-quinone and its fast decay. The experimental conditions were the same as S8 Fig. (TIF) [file pone.0177330.s008.TIF]

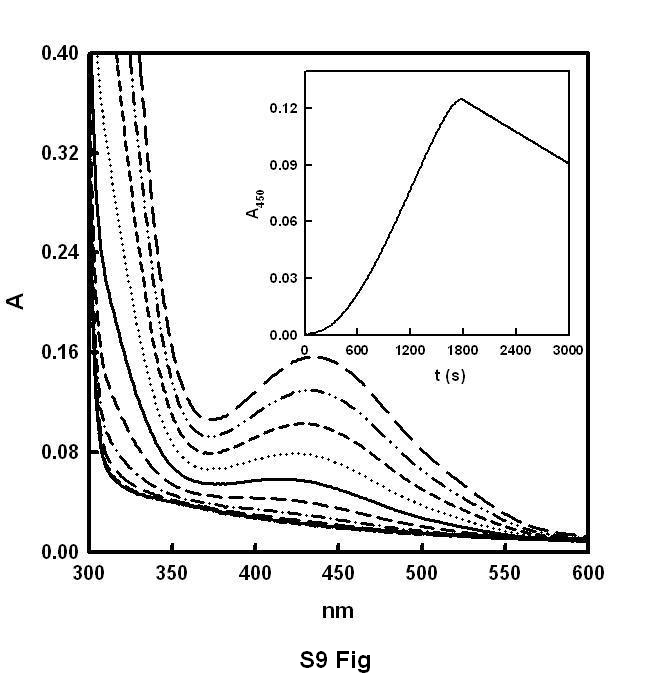

Supplement: S9 Fig — The experimental conditions were [E]0 = 50 nM and [β-arbutin]0 = 1 mM. The spectrophotometric recordings were made every 2 minutes. Inset. Instability of the o-quinone produced by the action of tyrosinase on β-arbutin. Recording of the formation time of the o-quinone and its fast decay. The experimental conditions were the same as S9 Fig. (TIF) [file pone.0177330.s009.TIF]

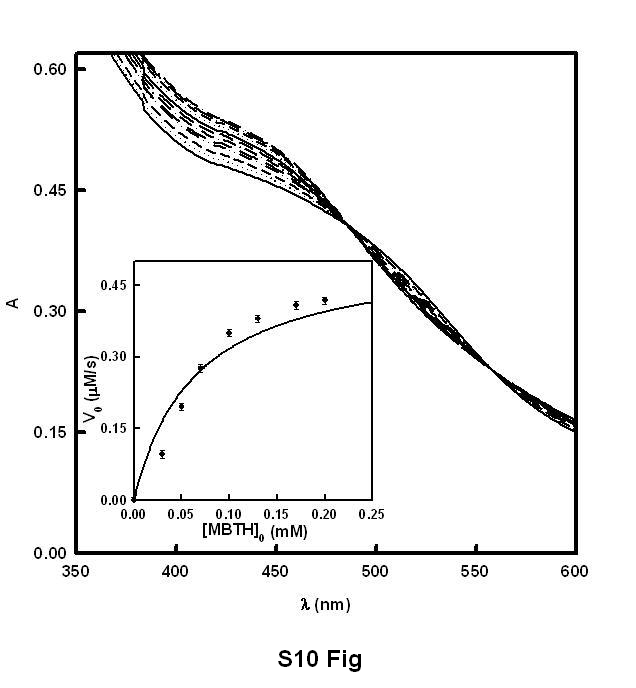

Supplement: S10 Fig — The experimental conditions were [E]0 = 300 nM, [MBTH]0 = 0.2 mM, [β-arbutin]0 = 10 μM and DMF 2%. The spectrophotometric recordings were made every 60 seconds. Inset. Determination of the MBTH saturation concentration. The experimental conditions were [E]0 = 100 nM, [β-arbutin]0 = 20 mM and DMF 2%. (TIF) [file pone.0177330.s010.TIF]

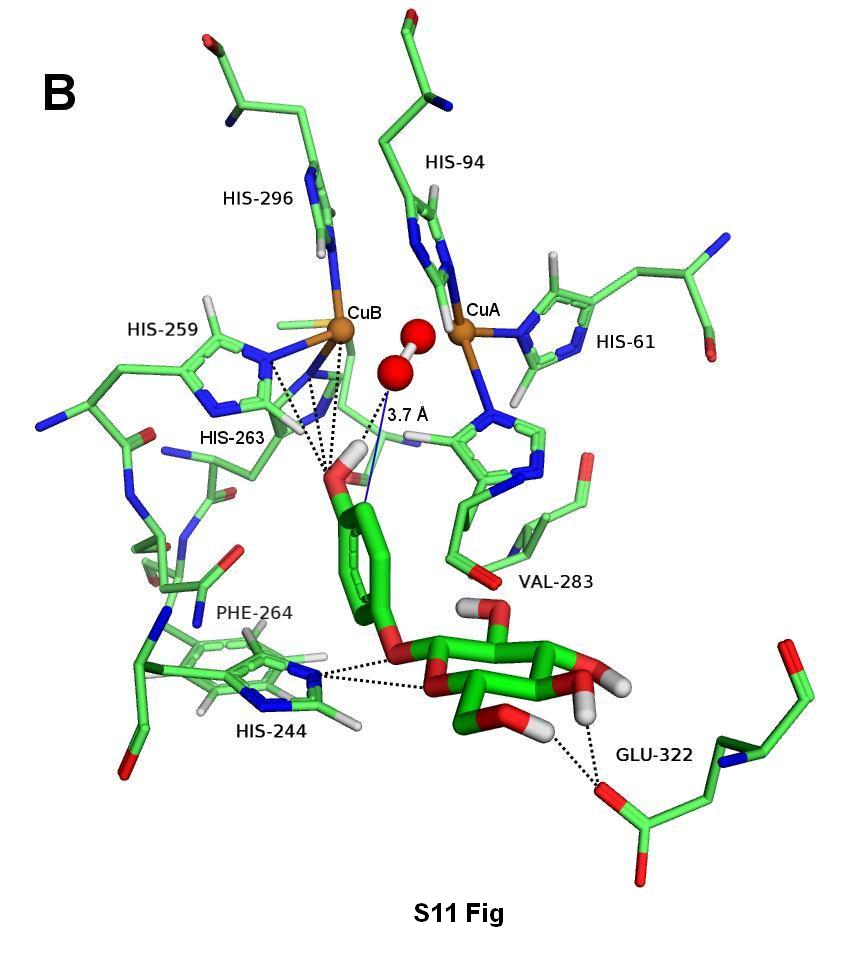

Supplement: S11 Fig — Docking poses obtained with AutoDock of β-arbutin in the active site of the oxy form of mushroom tyrosinase are shown as sticks. The color scheme is as described in Fig 8. (TIF) [file pone.0177330.s011.TIF]

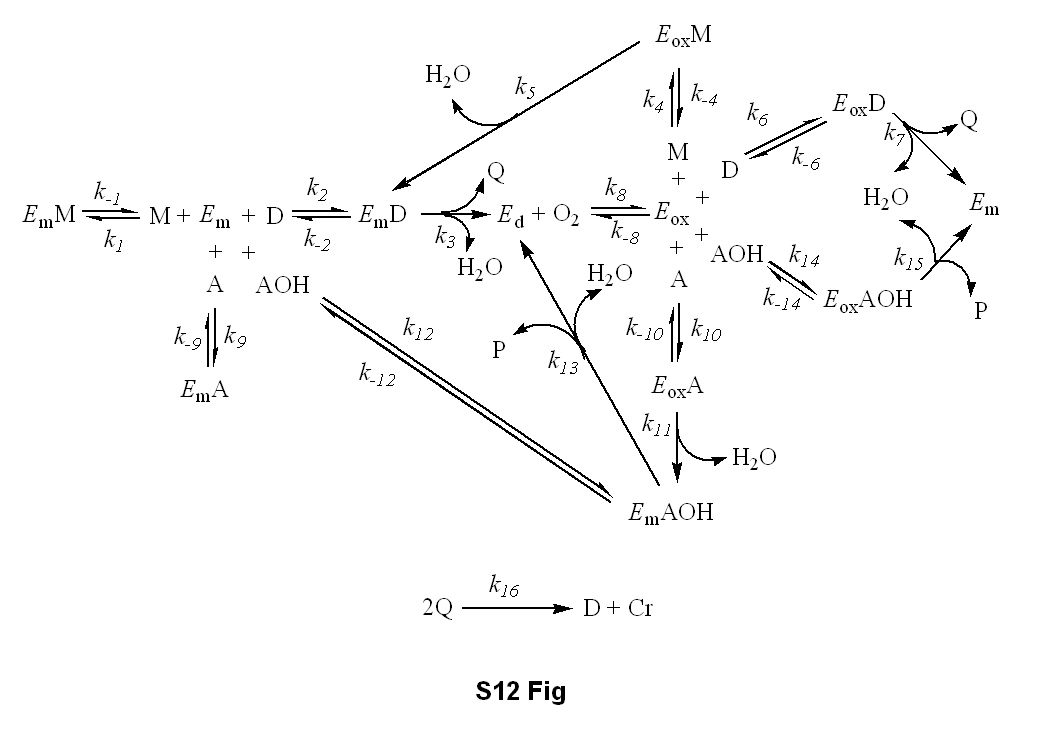

Supplement: S12 Fig — M = monophenol (L-tyrosine), D = o-diphenol (L-dopa), Q = o-dopaquinone, P = o-quinone from α or β-arbutin, AOH = ortho-hydroxylated arbutin, Cr = dopachrome, Em = metatyrosinase, Ed = deoxytyrosinase and Eox = oxytyrosinase. (TIF) [file pone.0177330.s012.tif]

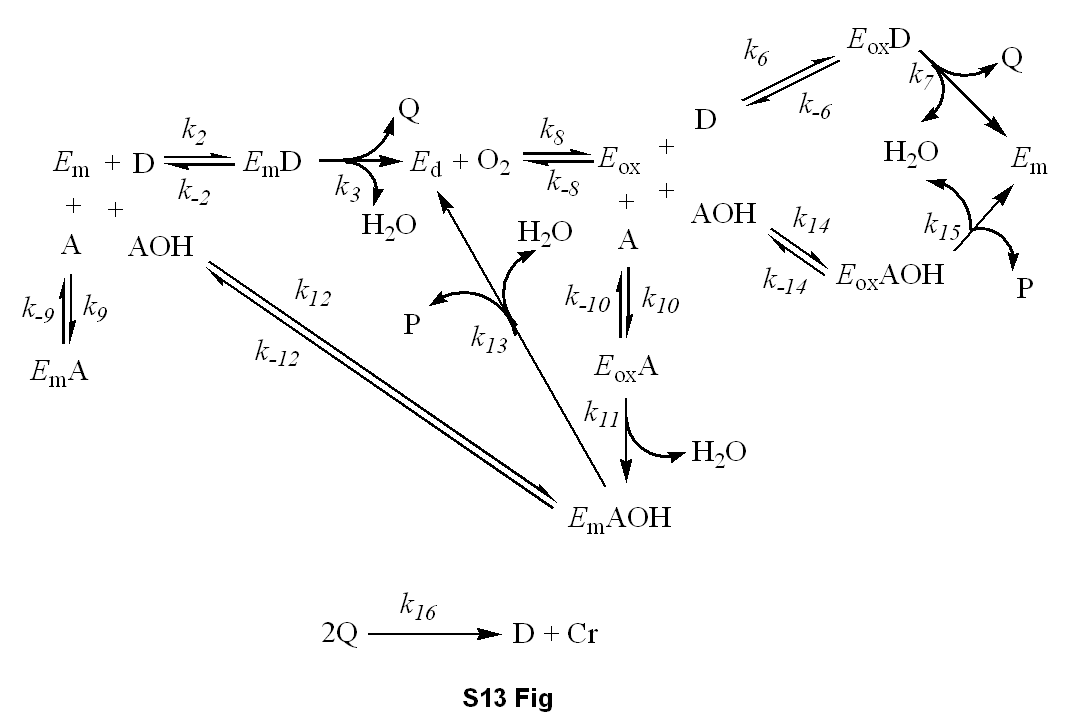

Supplement: S13 Fig — D = o-diphenol (L-dopa), A = Arbutin, Q = o-dopaquinone, P = o-quinone from α or β-arbutin, AOH = ortho-hydroxylated arbutin, Cr = dopachrome, Em = metatyrosinase, Ed = deoxytyrosinase and Eox = oxytyrosinase. (TIF) [file pone.0177330.s013.tif]
