# Supplementary material for: Action of tyrosinase on alpha and beta-arbutin: A kinetic study
Source: PLoS One. 2017 May 11;12(5):e0177330. doi: 10.1371/journal.pone.0177330 (PMC5426667; doi:10.1371/journal.pone.0177330)
Supplement: S1 File — (DOCX) [file pone.0177330.s014.docx]

**Kinetic analysis**

The mechanisms described in Figs 16 and 17 reflect the action of tyrosinase on its physiological substrates L-tyrosine (M) and L-dopa (D), in the presence of arbutin (A), which behaves as a substrate. The rate equations were obtained by means of the ALBASS program, designed specifically to obtain the rate equations for enzymatic reactions in steady-state [[1](#_ENREF_1)].

In the mechanisms described in Figs 16 and 17, the following notation was used:

*E*_m_ *met*-tyrosinase

*E*_d_ *deoxy*-tyrosinase

*E*_ox_ *oxy*-tyrosinase

*E*_m_D *met*-tyrosinase/L-dopa complex

*E*_ox_D *oxy*-tyrosinase/L-dopa complex

*E*_m_AOH *met*-tyrosinase/o-diphenol complex derived from A

*E*_ox_AOH *oxy*-tyrosinase/o-diphenol complex derived from A

*E*_m_M *met*-tyrosinase/L-tyrosine complex

*E*_ox_M *oxy*-tyrosinase/L-tyrosine complex

[*E*]_0_ initial concentration of tyrosinase

[D] L-dopa

[D]_0_ initial concentration in steady-state

A arbutin (α or β)

[A]_0_ initial concentration of arbutin

M L-tyrosine

[M]_0_ initial concentration of L-tyrosine

AOH *o*-diphenol corresponding to A

Q *o*-diphenol corresponding to L-dopa

P *o*-quinone corresponding to AOH

[P] instantaneous concentration of P

Cr dopachrome

[Cr] instantaneous concentration of dopachrome

$V_{0}^{\text{Cr}}$ initial rate of tyrosinase acting on L-dopa (D) or L-tyrosine (M) calculated by measuring Cr

$V_{0}^{\text{Cr}}$ initial rate of tyrosinase acting on L-dopa (D) or L-tyrosine (M) calculated by measuring Q

$V_{0}^{\text{M(D)(Q)}}$ initial rate of tyrosinase acting on L-tyrosine (M) in the presence of L-dopa (D) and calculated by measuring Q

$V_{0}^{\text{M(D)(Q)}}$ initial rate of tyrosinase acting on L-tyrosine (M) in the presence of L-dopa (D) and calculated by measuring Cr

$V_{0}^{\text{M(D)(A)(AOH)(Cr)}}$ initial rate of tyrosinase acting on L-tyrosine (M) in the presence of L-dopa (D), A and its *o*-diphenol AOH calculated by measuring Cr

$V_{0}^{\text{M(D)(A)(AOH)(Q)}}$ initial rate of tyrosinase acting on L-tyrosine (M) in the presence of L-dopa (D), A and its *o*-diphenol AOH calculated by measuring Q

$V_{0}^{\text{D(A)(Q)}}$ initial rate of tyrosinase acting on L-dopa (D) in the presence of A calculated by measuring Q

$V_{0}^{\text{D(A)(Cr)}}$ initial rate of tyrosinase acting on L-dopa (D) in the presence of A calculated by measuring Cr

R ratio between [D]_ss_ and [M]_ss_, R = [D]_ss_/[M]_ss_ [D]_ss_/[M]_0_

*k_i_* rate constants in the catalytic route (*i*<*k*_15_)

Applying the steady-state approximation to the mechanism described in Fig 16 for the activity of tyrosinase on monophenols and *o*-diphenols provides the following expression for the *o*-dopaquinone (Q) formation rate [[1](#_ENREF_1)]:

$\text{V}_{0}^{Q}=\frac{\left[\begin{matrix} \text{α}_{1}\left[O_{2}\right]_{0}\left[M\right]_{0}\left[AOH\right]_{0}+\text{α}_{2}\left[O_{2}\right]_{0}\left[D\right]_{0}\left[AOH\right]_{0}+ \\ \text{+α}_{3}\left[O_{2}\right]_{0}\left[D\right]_{0}\left[M\right]_{0}+\text{α}_{4}\left[O_{2}\right]_{0}\left[D\right]_{0}^{2}+\text{α}_{5}\left[O_{2}\right]_{0}\left[D\right]_{0}\left[A\right]_{0} \end{matrix}\right]\left[\text{E}\right]_{0}}{\begin{matrix} (\text{β}_{1}\left[D\right]_{0} +\text{ β}_{2}\left[D\right]_{0}+\text{ β}_{3}\left[AOH\right]_{0}^{2}+\text{ β}_{4}\left[M\right]_{0}\left[AOH\right]_{0} +\text{ } \\ \text{+β}_{5}\left[D\right]_{0}\left[AOH\right]_{0}+\text{β}_{6}\left[D\right]_{0}\left[M\right]_{0}+\text{ β}_{7}\left[D\right]_{0}^{2} +\text{β}_{8}\left[O_{2}\right]_{0}\left[AOH\right]_{0}+ \\ \text{+β}_{9}\left[M\right]_{0}\left[O_{2}\right]_{0}+\text{β}_{10}\left[O_{2}\right]_{0}\left[D\right]_{0}+\text{β}_{11}\left[A\right]_{0}\left[AOH\right]_{0}+ \\ \text{+β}_{12}\left[A\right]_{0}\left[D\right]_{0}+\text{β}_{13}\left[A\right]_{0}\left[O_{2}\right]_{0}+\text{β}_{14}\left[O_{2}\right]_{0}\left[AOH\right]_{0}^{2}+ \\ \text{+β}_{15}\left[M\right]_{0}\left[O_{2}\right]_{0}\left[AOH\right]_{0}+\text{β}_{16}\left[O_{2}\right]_{0}\left[M\right]_{0}^{2}+\text{β}_{17}\left[AOH\right]_{0}\left[O_{2}\right]_{0}\left[D\right]_{0}+ \\ \text{+β}_{18}\left[O_{2}\right]_{0}\left[D\right]_{0}\left[M\right]_{0}\text{+β}_{19}\left[O_{2}\right]_{0}\left[D\right]_{0}^{2}+\text{β}_{20}\left[O_{2}\right]_{0}\left[AOH\right]_{0}\left[A\right]_{0}+ \\ +\text{β}_{21}\left[A\right]_{0}\left[O_{2}\right]_{0}\left[M\right]_{0}+\text{β}_{22}\left[O_{2}\right]_{0}\left[AOH\right]_{0}\left[D\right]_{0}\text{+β}_{23}\left[O_{2}\right]_{0}\left[A\right]_{0}^{2}) \end{matrix}}$ (S1)

The oxygen concentration is saturating [[2](#_ENREF_2),[3](#_ENREF_3)], so [O_2_]_0_ 🡪 ∞ and the following expression is obtained:

$\text{V}_{0}^{Q}=\frac{\left[\begin{matrix} \text{α}_{1}\left[M\right]_{0}\left[AOH\right]_{0}+\text{α}_{2}\left[D\right]_{0}\left[AOH\right]_{0}+ \\ +\text{α}_{3}\left[D\right]_{0}\left[M\right]_{0}+\text{α}_{4}\left[D\right]_{0}^{2}+\text{α}_{5}\left[D\right]_{0}\left[A\right]_{0} \end{matrix}\right]\left[\text{E}\right]_{0}}{\begin{matrix} (\text{ β}_{8}\left[AOH\right]_{0}+\text{ β}_{9}\left[M\right]_{0} +\text{β}_{10}\left[D\right]_{0}\text{+β}_{13}\left[A\right]_{0}\text{+β}_{14}\left[AOH\right]_{0}^{2}\text{+} \\ \text{+β}_{15}\left[M\right]_{0}\left[AOH\right]_{0}\text{+β}_{16}\left[M\right]_{0}^{2}\text{+β}_{17}\left[D\right]_{0}\left[AOH\right]_{0}\text{+β}_{18}\left[D\right]_{0}\left[M\right]_{0}\text{+} \\ \text{+β}_{19}\left[D\right]_{0}^{2}\text{+β}_{20}\left[A\right]_{0}\left[AOH\right]_{0}+\text{β}_{21}\left[A\right]_{0}\left[M\right]_{0}\text{+β}_{22}\left[D\right]_{0}\left[AOH\right]_{0}\text{+β}_{23}\left[A\right]_{0}^{2}) \end{matrix}}$ (S2)

**Diphenolase activity**

Taking into account the diphenolase activity, the Fig 16 is simplified to Fig 17 due to [M]_0_ = 0. The *o*-dopaquinone (Q) formation rate is:

$\text{V}_{0}^{D(Q)}=\frac{\left[\left(\text{α}_{2}\left[AOH\right]_{0}+\text{α}_{5}\left[A\right]_{0}\right)\left[D\right]_{0}+\text{α}_{4}\left[D\right]_{0}^{2}\right]\left[\text{E}\right]_{0}}{\begin{matrix} (\text{ β}_{8}\left[AOH\right]_{0}+\text{β}_{10}\left[D\right]_{0}+\text{ β}_{13}\left[A\right]_{0}+\text{β}_{14}\left[AOH\right]_{0}^{2} +\text{β}_{17}\left[AOH\right]_{0}\left[D\right]_{0}+ \\ +\text{β}_{19}\left[D\right]_{0}^{2}\text{+β}_{20}\left[AOH\right]_{0}\left[A\right]_{0} +\text{β}_{22}\left[AOH\right]_{0}\left[D\right]_{0}) \end{matrix}}$ (S3)

and

$\text{V}_{0}^{D(Cr)}=\frac{\text{V}_{0}^{\mathrm{D}(\mathrm{Q})}}{2}$ (S4)

In the absence of arbutin [A] 🡪 0 and [AOH] 🡪 0, S3 equation becomes:

$\text{V}_{0}^{D(Q)}=\frac{\text{α}_{4}\left[D\right]_{0}^{2}\left[\text{E}\right]_{0}}{(\text{ β}_{10}\left[D\right]_{0}+\text{β}_{19}\left[D\right]_{0}^{2})}=\frac{\text{α}_{4}\left[D\right]_{0}\left[\text{E}\right]_{0}}{(\text{ β}_{10}+\text{β}_{19}\left[D\right]_{0})}$ (S5)

$\text{V}_{\max}^{Q}=\frac{\text{α}_{4}\left[\text{E}\right]_{0}}{\text{β}_{19}}$ (S6)

$\text{V}_{0}^{Cr}=\frac{\text{V}_{0}^{Q}}{2}=\frac{\text{α}_{3}\left[\text{E}\right]_{0}}{\text{β}_{14}2}=\frac{\text{k}_{3}\text{k}_{7}}{\text{k}_{3}\text{+k}_{7}}=\text{V}_{\max}^{D(Cr)}$ (S7)

However, if the L-dopa concentration is saturating, [D] 🡪 ∞, S3 equation becomes S5 equation, where $\text{V}_{\max}^{Q}$ and $\text{V}_{\max}^{D(Cr)}$ agree with S6 and S7 equations.

**Monophenolase activity**

Taking into account that [D] = R [M], S2 equation becomes:

$\text{V}_{0}^{M(Q)}=\frac{\left[\text{α}_{1}\left[AOH\right]_{0}+\text{α}_{1}\left[AOH\right]_{0}R+\text{α}_{5}R\left[A\right]_{0}\right]\left[M\right]_{0}\text{+}\left[\text{α}_{3}R+\text{α}_{4}R^{2}\right]\left[M\right]_{0}^{2}}{\begin{matrix} (\text{β}_{8}\left[AOH\right]_{0}+\text{β}_{13}\left[A\right]_{0}\text{+β}_{14}\left[AOH\right]_{0}^{2}+\text{β}_{20}\left[AOH\right]_{0}\left[A\right]_{0}+\text{β}_{23}\left[A\right]_{0}^{2}+ \\ +\left[\begin{matrix} \text{β}_{9}+\left[A\right]_{0}\text{+β}_{10}\mathrm{R}+\text{β}_{15}\left[AOH\right]_{0}\text{+β}_{17}R\left[AOH\right]_{0}+ \\ +\text{β}_{21}\left[A\right]_{0}+\text{β}_{22}\left[AOH\right]_{0}R \end{matrix}\right]\left[M\right]_{0}+ \\ +\left[\text{β}_{16}\text{+β}_{18}\mathrm{R}+\text{β}_{19}R^{2}\right]\left[M\right]_{0}^{2} \end{matrix}}$ (S8)

and

$\text{V}_{0}^{M(D)(A)(Cr)}=\frac{\text{V}_{0}^{M(D)(A)(\mathrm{Q})}}{2}$ (S9)

In the absence of arbutin [A] 🡪 0 and [AOH] 🡪 0, S8 becomes:

$\text{V}_{0}^{M(Q)}=\frac{\text{α}_{3}R+\text{α}_{4}R^{2}\left[M\right]_{0}}{\text{β}_{9} +\text{β}_{10}R+\left[\text{β}_{16}\text{+β}_{18}R+\text{β}_{19}R^{2}\right]\left[M\right]_{0}}\left[\text{E}\right]_{0}$ (S10)

and

$\text{V}_{\max}^{M(Q)}=\text{V}_{\max}^{M(D)(Q)}=\frac{\text{α}_{3}R+\text{α}_{4}R^{2}}{\text{β}_{16}\text{+β}_{18}R+\text{β}_{19}R^{2}}\left[\text{E}\right]_{0}=4\text{k}_{5}\left[\text{E}\right]_{0}$ (S11)

$\text{V}_{\max}^{M(D)(Cr)}=2\text{k}_{5}\left[\text{E}\right]_{0}$ (S12)

In the presence of arbutin, and if [M] → ∞, S11 and S12 equations are obtained from 8S equation. Therefore, the maximum rate of tyrosinase acting on monophenols in the presence of arbutin does not vary, as would be the case with a competitive inhibitor or an alternative substrate.

The constants are:

α_1_ = {*K*_1_(*k*_+10_)(*k*_+3_)(*k*_+8_)(*k*_+4_)(*k*_+7_)(*k*_+5_)*K*_9_(*k*_+11_)(*k*_-13_)(*k*_+15_) 
         _+_ *K*_1_(*k*_+10_)(*k*_+3_)(*k*_+8_)(*k*_+4_)(*k*_+7_)(*k*_+5_)*K*_9_(*k*_+11_)(*k*_-13_)(*k*_-14_) 
         _+_ *K*_1_(*k*_+10_)(*k*_+3_)(*k*_+8_)(*k*_+4_)(*k*_+7_)(*k*_+5_)*K*_9_(*k*_+11_)(*k*_+12_)(*k*_+15_) 
         _+_ *K*_1_(*k*_+10_)(*k*_+3_)(*k*_+8_)(*k*_+4_)(*k*_+7_)(*k*_+5_)*K*_9_(*k*_+11_)(*k*_+12_)(*k*_-14_) 
         _+_ *K*_1_(*k*_+10_)(*k*_+3_)(*k*_+8_)(*k*_+4_)(*k*_-6_)(*k*_+5_)*K*_9_(*k*_+11_)(*k*_-13_)(*k*_+15_) 
         _+_ *K*_1_(*k*_+10_)(*k*_+3_)(*k*_+8_)(*k*_+4_)(*k*_-6_)(*k*_+5_)*K*_9_(*k*_+11_)(*k*_-13_)(*k*_-14_) 
         _+_ *K*_1_(*k*_+10_)(*k*_+3_)(*k*_+8_)(*k*_+4_)(*k*_-6_)(*k*_+5_)*K*_9_(*k*_+11_)(*k*_+12_)(*k*_+15_) 
         _+_ *K*_1_(*k*_+10_)(*k*_+3_)(*k*_+8_)(*k*_+4_)(*k*_-6_)(*k*_+5_)*K*_9_(*k*_+11_)(*k*_+12_)(*k*_-14_)}

α_2_ = {*K*_1_(*k*_+2_)(*k*_+3_)(*k*_+8_)(*k*_+14_)(*k*_+7_)(*k*_+5_)*K*_9_(*k*_-10_)(*k*_-13_)(*k*_+15_) 
         _+_ *K*_1_(*k*_+2_)(*k*_+3_)(*k*_+8_)(*k*_+14_)(*k*_+7_)(*k*_+5_)*K*_9_(*k*_-10_)(*k*_+12_)(*k*_+15_) 
         _+_ *K*_1_(*k*_+2_)(*k*_+3_)(*k*_+8_)(*k*_+14_)(*k*_+7_)(*k*_+5_)*K*_9_(*k*_+11_)(*k*_-13_)(*k*_+15_) 
         _+_ *K*_1_(*k*_+2_)(*k*_+3_)(*k*_+8_)(*k*_+14_)(*k*_+7_)(*k*_+5_)*K*_9_(*k*_+11_)(*k*_+12_)(*k*_+15_) 
         _+_ *K*_1_(*k*_+2_)(*k*_+3_)(*k*_+8_)(*k*_+14_)(*k*_+7_)(*k*_-4_)*K*_9_(*k*_-10_)(*k*_-13_)(*k*_+15_) 
         _+_ *K*_1_(*k*_+2_)(*k*_+3_)(*k*_+8_)(*k*_+14_)(*k*_+7_)(*k*_-4_)*K*_9_(*k*_-10_)(*k*_+12_)(*k*_+15_) 
         _+_ *K*_1_(*k*_+2_)(*k*_+3_)(*k*_+8_)(*k*_+14_)(*k*_+7_)(*k*_-4_)*K*_9_(*k*_+11_)(*k*_-13_)(*k*_+15_) 
         _+_ *K*_1_(*k*_+2_)(*k*_+3_)(*k*_+8_)(*k*_+14_)(*k*_+7_)(*k*_-4_)*K*_9_(*k*_+11_)(*k*_+12_)(*k*_+15_) 
         _+_ *K*_1_(*k*_+2_)(*k*_+3_)(*k*_+8_)(*k*_+14_)(*k*_-6_)(*k*_+5_)*K*_9_(*k*_-10_)(*k*_-13_)(*k*_+15_) 
         _+_ *K*_1_(*k*_+2_)(*k*_+3_)(*k*_+8_)(*k*_+14_)(*k*_-6_)(*k*_+5_)*K*_9_(*k*_-10_)(*k*_+12_)(*k*_+15_) 
         _+_ *K*_1_(*k*_+2_)(*k*_+3_)(*k*_+8_)(*k*_+14_)(*k*_-6_)(*k*_+5_)*K*_9_(*k*_+11_)(*k*_-13_)(*k*_+15_) 
         _+_ *K*_1_(*k*_+2_)(*k*_+3_)(*k*_+8_)(*k*_+14_)(*k*_-6_)(*k*_+5_)*K*_9_(*k*_+11_)(*k*_+12_)(*k*_+15_) 
         _+_ *K*_1_(*k*_+2_)(*k*_+3_)(*k*_+8_)(*k*_+14_)(*k*_-6_)(*k*_-4_)*K*_9_(*k*_-10_)(*k*_-13_)(*k*_+15_) 
         _+_ *K*_1_(*k*_+2_)(*k*_+3_)(*k*_+8_)(*k*_+14_)(*k*_-6_)(*k*_-4_)*K*_9_(*k*_-10_)(*k*_+12_)(*k*_+15_) 
         _+_ *K*_1_(*k*_+2_)(*k*_+3_)(*k*_+8_)(*k*_+14_)(*k*_-6_)(*k*_-4_)*K*_9_(*k*_+11_)(*k*_-13_)(*k*_+15_) 
         _+_ *K*_1_(*k*_+2_)(*k*_+3_)(*k*_+8_)(*k*_+14_)(*k*_-6_)(*k*_-4_)*K*_9_(*k*_+11_)(*k*_+12_)(*k*_+15_) 
         _+_ *K*_1_(*k*_+10_)(*k*_-2_)(*k*_+8_)(*k*_+6_)(*k*_+7_)(*k*_+5_)*K*_9_(*k*_+11_)(*k*_-13_)(*k*_+15_) 
         _+_ *K*_1_(*k*_+10_)(*k*_-2_)(*k*_+8_)(*k*_+6_)(*k*_+7_)(*k*_+5_)*K*_9_(*k*_+11_)(*k*_-13_)(*k*_-14_) 
         _+_ *K*_1_(*k*_+10_)(*k*_-2_)(*k*_+8_)(*k*_+6_)(*k*_+7_)(*k*_+5_)*K*_9_(*k*_+11_)(*k*_+12_)(*k*_+15_) 
         _+_ *K*_1_(*k*_+10_)(*k*_-2_)(*k*_+8_)(*k*_+6_)(*k*_+7_)(*k*_+5_)*K*_9_(*k*_+11_)(*k*_+12_)(*k*_-14_) 
         _+_ *K*_1_(*k*_+10_)(*k*_-2_)(*k*_+8_)(*k*_+6_)(*k*_+7_)(*k*_-4_)*K*_9_(*k*_+11_)(*k*_-13_)(*k*_+15_) 
         _+_ *K*_1_(*k*_+10_)(*k*_-2_)(*k*_+8_)(*k*_+6_)(*k*_+7_)(*k*_-4_)*K*_9_(*k*_+11_)(*k*_-13_)(*k*_-14_) 
         _+_ *K*_1_(*k*_+10_)(*k*_-2_)(*k*_+8_)(*k*_+6_)(*k*_+7_)(*k*_-4_)*K*_9_(*k*_+11_)(*k*_+12_)(*k*_+15_) 
         _+_ *K*_1_(*k*_+10_)(*k*_-2_)(*k*_+8_)(*k*_+6_)(*k*_+7_)(*k*_-4_)*K*_9_(*k*_+11_)(*k*_+12_)(*k*_-14_) 
         _+_ *K*_1_(*k*_+10_)(*k*_+3_)(*k*_+8_)(*k*_+6_)(*k*_+7_)(*k*_+5_)*K*_9_(*k*_+11_)(*k*_-13_)(*k*_+15_) 
         _+_ *K*_1_(*k*_+10_)(*k*_+3_)(*k*_+8_)(*k*_+6_)(*k*_+7_)(*k*_+5_)*K*_9_(*k*_+11_)(*k*_-13_)(*k*_-14_) 
         _+_ *K*_1_(*k*_+10_)(*k*_+3_)(*k*_+8_)(*k*_+6_)(*k*_+7_)(*k*_+5_)*K*_9_(*k*_+11_)(*k*_+12_)(*k*_+15_) 
         _+_ *K*_1_(*k*_+10_)(*k*_+3_)(*k*_+8_)(*k*_+6_)(*k*_+7_)(*k*_+5_)*K*_9_(*k*_+11_)(*k*_+12_)(*k*_-14_) 
         _+_ *K*_1_(*k*_+10_)(*k*_+3_)(*k*_+8_)(*k*_+6_)(*k*_+7_)(*k*_-4_)*K*_9_(*k*_+11_)(*k*_-13_)(*k*_+15_) 
         _+_ *K*_1_(*k*_+10_)(*k*_+3_)(*k*_+8_)(*k*_+6_)(*k*_+7_)(*k*_-4_)*K*_9_(*k*_+11_)(*k*_-13_)(*k*_-14_) 
         _+_ *K*_1_(*k*_+10_)(*k*_+3_)(*k*_+8_)(*k*_+6_)(*k*_+7_)(*k*_-4_)*K*_9_(*k*_+11_)(*k*_+12_)(*k*_+15_) 
         _+_ *K*_1_(*k*_+10_)(*k*_+3_)(*k*_+8_)(*k*_+6_)(*k*_+7_)(*k*_-4_)*K*_9_(*k*_+11_)(*k*_+12_)(*k*_-14_)}
α_3_ = {*K*_1_(*k*_+2_)(*k*_+3_)(*k*_+8_)(*k*_+4_)(*k*_+7_)(*k*_+5_)*K*_9_(*k*_-10_)(*k*_-13_)(*k*_+15_) 
         _+_ *K*_1_(*k*_+2_)(*k*_+3_)(*k*_+8_)(*k*_+4_)(*k*_+7_)(*k*_+5_)*K*_9_(*k*_-10_)(*k*_-13_)(*k*_-14_) 
         _+_ *K*_1_(*k*_+2_)(*k*_+3_)(*k*_+8_)(*k*_+4_)(*k*_+7_)(*k*_+5_)*K*_9_(*k*_-10_)(*k*_+12_)(*k*_+15_) 
         _+_ *K*_1_(*k*_+2_)(*k*_+3_)(*k*_+8_)(*k*_+4_)(*k*_+7_)(*k*_+5_)*K*_9_(*k*_-10_)(*k*_+12_)(*k*_-14_) 
         _+_ *K*_1_(*k*_+2_)(*k*_+3_)(*k*_+8_)(*k*_+4_)(*k*_+7_)(*k*_+5_)*K*_9_(*k*_+11_)(*k*_-13_)(*k*_+15_) 
         _+_ *K*_1_(*k*_+2_)(*k*_+3_)(*k*_+8_)(*k*_+4_)(*k*_+7_)(*k*_+5_)*K*_9_(*k*_+11_)(*k*_-13_)(*k*_-14_) 
         _+_ *K*_1_(*k*_+2_)(*k*_+3_)(*k*_+8_)(*k*_+4_)(*k*_+7_)(*k*_+5_)*K*_9_(*k*_+11_)(*k*_+12_)(*k*_+15_) 
         _+_ *K*_1_(*k*_+2_)(*k*_+3_)(*k*_+8_)(*k*_+4_)(*k*_+7_)(*k*_+5_)*K*_9_(*k*_+11_)(*k*_+12_)(*k*_-14_) 
         _+_ *K*_1_(*k*_+2_)(*k*_+3_)(*k*_+8_)(*k*_+4_)(*k*_-6_)(*k*_+5_)*K*_9_(*k*_-10_)(*k*_-13_)(*k*_+15_) 
         _+_ *K*_1_(*k*_+2_)(*k*_+3_)(*k*_+8_)(*k*_+4_)(*k*_-6_)(*k*_+5_)*K*_9_(*k*_-10_)(*k*_-13_)(*k*_-14_) 
         _+_ *K*_1_(*k*_+2_)(*k*_+3_)(*k*_+8_)(*k*_+4_)(*k*_-6_)(*k*_+5_)*K*_9_(*k*_-10_)(*k*_+12_)(*k*_+15_) 
         _+_ *K*_1_(*k*_+2_)(*k*_+3_)(*k*_+8_)(*k*_+4_)(*k*_-6_)(*k*_+5_)*K*_9_(*k*_-10_)(*k*_+12_)(*k*_-14_) 
         _+_ *K*_1_(*k*_+2_)(*k*_+3_)(*k*_+8_)(*k*_+4_)(*k*_-6_)(*k*_+5_)*K*_9_(*k*_+11_)(*k*_-13_)(*k*_+15_) 
         _+_ *K*_1_(*k*_+2_)(*k*_+3_)(*k*_+8_)(*k*_+4_)(*k*_-6_)(*k*_+5_)*K*_9_(*k*_+11_)(*k*_-13_)(*k*_-14_) 
         _+_ *K*_1_(*k*_+2_)(*k*_+3_)(*k*_+8_)(*k*_+4_)(*k*_-6_)(*k*_+5_)*K*_9_(*k*_+11_)(*k*_+12_)(*k*_+15_) 
         _+_ *K*_1_(*k*_+2_)(*k*_+3_)(*k*_+8_)(*k*_+4_)(*k*_-6_)(*k*_+5_)*K*_9_(*k*_+11_)(*k*_+12_)(*k*_-14_)}
α_4_ = {*K*_1_(*k*_+2_)(*k*_+3_)(*k*_+8_)(*k*_+6_)(*k*_+7_)(*k*_+5_)*K*_9_(*k*_-10_)(*k*_-13_)(*k*_+15_) 
         _+_ *K*_1_(*k*_+2_)(*k*_+3_)(*k*_+8_)(*k*_+6_)(*k*_+7_)(*k*_+5_)*K*_9_(*k*_-10_)(*k*_-13_)(*k*_-14_) 
         _+_ *K*_1_(*k*_+2_)(*k*_+3_)(*k*_+8_)(*k*_+6_)(*k*_+7_)(*k*_+5_)*K*_9_(*k*_-10_)(*k*_+12_)(*k*_+15_) 
         _+_ *K*_1_(*k*_+2_)(*k*_+3_)(*k*_+8_)(*k*_+6_)(*k*_+7_)(*k*_+5_)*K*_9_(*k*_-10_)(*k*_+12_)(*k*_-14_) 
         _+_ *K*_1_(*k*_+2_)(*k*_+3_)(*k*_+8_)(*k*_+6_)(*k*_+7_)(*k*_+5_)*K*_9_(*k*_+11_)(*k*_-13_)(*k*_+15_) 
         _+_ *K*_1_(*k*_+2_)(*k*_+3_)(*k*_+8_)(*k*_+6_)(*k*_+7_)(*k*_+5_)*K*_9_(*k*_+11_)(*k*_-13_)(*k*_-14_) 
         _+_ *K*_1_(*k*_+2_)(*k*_+3_)(*k*_+8_)(*k*_+6_)(*k*_+7_)(*k*_+5_)*K*_9_(*k*_+11_)(*k*_+12_)(*k*_+15_) 
         _+_ *K*_1_(*k*_+2_)(*k*_+3_)(*k*_+8_)(*k*_+6_)(*k*_+7_)(*k*_+5_)*K*_9_(*k*_+11_)(*k*_+12_)(*k*_-14_) 
         _+_ *K*_1_(*k*_+2_)(*k*_+3_)(*k*_+8_)(*k*_+6_)(*k*_+7_)(*k*_-4_)*K*_9_(*k*_-10_)(*k*_-13_)(*k*_+15_) 
         _+_ *K*_1_(*k*_+2_)(*k*_+3_)(*k*_+8_)(*k*_+6_)(*k*_+7_)(*k*_-4_)*K*_9_(*k*_-10_)(*k*_-13_)(*k*_-14_) 
         _+_ *K*_1_(*k*_+2_)(*k*_+3_)(*k*_+8_)(*k*_+6_)(*k*_+7_)(*k*_-4_)*K*_9_(*k*_-10_)(*k*_+12_)(*k*_+15_) 
         _+_ *K*_1_(*k*_+2_)(*k*_+3_)(*k*_+8_)(*k*_+6_)(*k*_+7_)(*k*_-4_)*K*_9_(*k*_-10_)(*k*_+12_)(*k*_-14_) 
         _+_ *K*_1_(*k*_+2_)(*k*_+3_)(*k*_+8_)(*k*_+6_)(*k*_+7_)(*k*_-4_)*K*_9_(*k*_+11_)(*k*_-13_)(*k*_+15_) 
         _+_ *K*_1_(*k*_+2_)(*k*_+3_)(*k*_+8_)(*k*_+6_)(*k*_+7_)(*k*_-4_)*K*_9_(*k*_+11_)(*k*_-13_)(*k*_-14_) 
         _+_ *K*_1_(*k*_+2_)(*k*_+3_)(*k*_+8_)(*k*_+6_)(*k*_+7_)(*k*_-4_)*K*_9_(*k*_+11_)(*k*_+12_)(*k*_+15_) 
         _+_ *K*_1_(*k*_+2_)(*k*_+3_)(*k*_+8_)(*k*_+6_)(*k*_+7_)(*k*_-4_)*K*_9_(*k*_+11_)(*k*_+12_)(*k*_-14_) 
         _+_ *K*_1_(*k*_+2_)(*k*_+3_)(*k*_+8_)(*k*_+6_)(*k*_+7_)(*k*_+5_)*K*_9_(*k*_-10_)(*k*_-13_)(*k*_+15_) 
         _+_ *K*_1_(*k*_+2_)(*k*_+3_)(*k*_+8_)(*k*_+6_)(*k*_+7_)(*k*_+5_)*K*_9_(*k*_-10_)(*k*_-13_)(*k*_-14_) 
         _+_ *K*_1_(*k*_+2_)(*k*_+3_)(*k*_+8_)(*k*_+6_)(*k*_+7_)(*k*_+5_)*K*_9_(*k*_-10_)(*k*_+12_)(*k*_+15_) 
         _+_ *K*_1_(*k*_+2_)(*k*_+3_)(*k*_+8_)(*k*_+6_)(*k*_+7_)(*k*_+5_)*K*_9_(*k*_-10_)(*k*_+12_)(*k*_-14_) 
         _+_ *K*_1_(*k*_+2_)(*k*_+3_)(*k*_+8_)(*k*_+6_)(*k*_+7_)(*k*_+5_)*K*_9_(*k*_+11_)(*k*_-13_)(*k*_+15_) 
         _+_ *K*_1_(*k*_+2_)(*k*_+3_)(*k*_+8_)(*k*_+6_)(*k*_+7_)(*k*_+5_)*K*_9_(*k*_+11_)(*k*_-13_)(*k*_-14_) 
         _+_ *K*_1_(*k*_+2_)(*k*_+3_)(*k*_+8_)(*k*_+6_)(*k*_+7_)(*k*_+5_)*K*_9_(*k*_+11_)(*k*_+12_)(*k*_+15_) 
         _+_ *K*_1_(*k*_+2_)(*k*_+3_)(*k*_+8_)(*k*_+6_)(*k*_+7_)(*k*_+5_)*K*_9_(*k*_+11_)(*k*_+12_)(*k*_-14_) 
         _+_ *K*_1_(*k*_+2_)(*k*_+3_)(*k*_+8_)(*k*_+6_)(*k*_+7_)(*k*_-4_)*K*_9_(*k*_-10_)(*k*_-13_)(*k*_+15_) 
         _+_ *K*_1_(*k*_+2_)(*k*_+3_)(*k*_+8_)(*k*_+6_)(*k*_+7_)(*k*_-4_)*K*_9_(*k*_-10_)(*k*_-13_)(*k*_-14_) 
         _+_ *K*_1_(*k*_+2_)(*k*_+3_)(*k*_+8_)(*k*_+6_)(*k*_+7_)(*k*_-4_)*K*_9_(*k*_-10_)(*k*_+12_)(*k*_+15_) 
         _+_ *K*_1_(*k*_+2_)(*k*_+3_)(*k*_+8_)(*k*_+6_)(*k*_+7_)(*k*_-4_)*K*_9_(*k*_-10_)(*k*_+12_)(*k*_-14_) 
         _+_ *K*_1_(*k*_+2_)(*k*_+3_)(*k*_+8_)(*k*_+6_)(*k*_+7_)(*k*_-4_)*K*_9_(*k*_+11_)(*k*_-13_)(*k*_+15_) 
         _+_ *K*_1_(*k*_+2_)(*k*_+3_)(*k*_+8_)(*k*_+6_)(*k*_+7_)(*k*_-4_)*K*_9_(*k*_+11_)(*k*_-13_)(*k*_-14_) 
         _+_ *K*_1_(*k*_+2_)(*k*_+3_)(*k*_+8_)(*k*_+6_)(*k*_+7_)(*k*_-4_)*K*_9_(*k*_+11_)(*k*_+12_)(*k*_+15_) 
         _+_ *K*_1_(*k*_+2_)(*k*_+3_)(*k*_+8_)(*k*_+6_)(*k*_+7_)(*k*_-4_)*K*_9_(*k*_+11_)(*k*_+12_)(*k*_-14_)}
α_5_ = {*K*_1_(*k*_+2_)(*k*_+3_)(*k*_+8_)(*k*_+13_)(*k*_+7_)(*k*_+5_)*K*_9_(*k*_-10_)(*k*_+12_)(*k*_+15_) 
         _+_ *K*_1_(*k*_+2_)(*k*_+3_)(*k*_+8_)(*k*_+13_)(*k*_+7_)(*k*_+5_)*K*_9_(*k*_-10_)(*k*_+12_)(*k*_-14_) 
         _+_ *K*_1_(*k*_+2_)(*k*_+3_)(*k*_+8_)(*k*_+13_)(*k*_+7_)(*k*_-4_)*K*_9_(*k*_-10_)(*k*_+12_)(*k*_+15_) 
         _+_ *K*_1_(*k*_+2_)(*k*_+3_)(*k*_+8_)(*k*_+13_)(*k*_+7_)(*k*_-4_)*K*_9_(*k*_-10_)(*k*_+12_)(*k*_-14_) 
         _+_ *K*_1_(*k*_+2_)(*k*_+3_)(*k*_+8_)(*k*_+13_)(*k*_-6_)(*k*_+5_)*K*_9_(*k*_-10_)(*k*_+12_)(*k*_+15_) 
         _+_ *K*_1_(*k*_+2_)(*k*_+3_)(*k*_+8_)(*k*_+13_)(*k*_-6_)(*k*_+5_)*K*_9_(*k*_-10_)(*k*_+12_)(*k*_-14_) 
         _+_ *K*_1_(*k*_+2_)(*k*_+3_)(*k*_+8_)(*k*_+13_)(*k*_-6_)(*k*_-4_)*K*_9_(*k*_-10_)(*k*_+12_)(*k*_+15_) 
         _+_ *K*_1_(*k*_+2_)(*k*_+3_)(*k*_+8_)(*k*_+13_)(*k*_-6_)(*k*_-4_)*K*_9_(*k*_-10_)(*k*_+12_)(*k*_-14_)}

and


β_1_ = {*K*_1_(*k*_+10_)(*k*_-2_)(*k*_-8_)(*k*_+7_)(*k*_+5_)*K*_9_(*k*_+11_)(*k*_-13_)(*k*_+15_) 
         _+_ *K*_1_(*k*_+10_)(*k*_-2_)(*k*_-8_)(*k*_+7_)(*k*_+5_)*K*_9_(*k*_+11_)(*k*_-13_)(*k*_-14_) 
         _+_ *K*_1_(*k*_+10_)(*k*_-2_)(*k*_-8_)(*k*_+7_)(*k*_+5_)*K*_9_(*k*_+11_)(*k*_+12_)(*k*_+15_) 
         _+_ *K*_1_(*k*_+10_)(*k*_-2_)(*k*_-8_)(*k*_+7_)(*k*_+5_)*K*_9_(*k*_+11_)(*k*_+12_)(*k*_-14_) 
         _+_ *K*_1_(*k*_+10_)(*k*_-2_)(*k*_-8_)(*k*_+7_)(*k*_-4_)*K*_9_(*k*_+11_)(*k*_-13_)(*k*_+15_) 
         _+_ *K*_1_(*k*_+10_)(*k*_-2_)(*k*_-8_)(*k*_+7_)(*k*_-4_)*K*_9_(*k*_+11_)(*k*_-13_)(*k*_-14_) 
         _+_ *K*_1_(*k*_+10_)(*k*_-2_)(*k*_-8_)(*k*_+7_)(*k*_-4_)*K*_9_(*k*_+11_)(*k*_+12_)(*k*_+15_) 
         _+_ *K*_1_(*k*_+10_)(*k*_-2_)(*k*_-8_)(*k*_+7_)(*k*_-4_)*K*_9_(*k*_+11_)(*k*_+12_)(*k*_-14_) 
         _+_ *K*_1_(*k*_+10_)(*k*_-2_)(*k*_-8_)(*k*_-6_)(*k*_+5_)*K*_9_(*k*_+11_)(*k*_-13_)(*k*_+15_) 
         _+_ *K*_1_(*k*_+10_)(*k*_-2_)(*k*_-8_)(*k*_-6_)(*k*_+5_)*K*_9_(*k*_+11_)(*k*_-13_)(*k*_-14_) 
         _+_ *K*_1_(*k*_+10_)(*k*_-2_)(*k*_-8_)(*k*_-6_)(*k*_+5_)*K*_9_(*k*_+11_)(*k*_+12_)(*k*_+15_) 
         _+_ *K*_1_(*k*_+10_)(*k*_-2_)(*k*_-8_)(*k*_-6_)(*k*_+5_)*K*_9_(*k*_+11_)(*k*_+12_)(*k*_-14_) 
         _+_ *K*_1_(*k*_+10_)(*k*_-2_)(*k*_-8_)(*k*_-6_)(*k*_-4_)*K*_9_(*k*_+11_)(*k*_-13_)(*k*_+15_) 
         _+_ *K*_1_(*k*_+10_)(*k*_-2_)(*k*_-8_)(*k*_-6_)(*k*_-4_)*K*_9_(*k*_+11_)(*k*_-13_)(*k*_-14_) 
         _+_ *K*_1_(*k*_+10_)(*k*_-2_)(*k*_-8_)(*k*_-6_)(*k*_-4_)*K*_9_(*k*_+11_)(*k*_+12_)(*k*_+15_) 
         _+_ *K*_1_(*k*_+10_)(*k*_-2_)(*k*_-8_)(*k*_-6_)(*k*_-4_)*K*_9_(*k*_+11_)(*k*_+12_)(*k*_-14_) 
         _+_ *K*_1_(*k*_+10_)(*k*_+3_)(*k*_-8_)(*k*_+7_)(*k*_+5_)*K*_9_(*k*_+11_)(*k*_-13_)(*k*_+15_) 
         _+_ *K*_1_(*k*_+10_)(*k*_+3_)(*k*_-8_)(*k*_+7_)(*k*_+5_)*K*_9_(*k*_+11_)(*k*_-13_)(*k*_-14_) 
         _+_ *K*_1_(*k*_+10_)(*k*_+3_)(*k*_-8_)(*k*_+7_)(*k*_+5_)*K*_9_(*k*_+11_)(*k*_+12_)(*k*_+15_) 
         _+_ *K*_1_(*k*_+10_)(*k*_+3_)(*k*_-8_)(*k*_+7_)(*k*_+5_)*K*_9_(*k*_+11_)(*k*_+12_)(*k*_-14_) 
         _+_ *K*_1_(*k*_+10_)(*k*_+3_)(*k*_-8_)(*k*_+7_)(*k*_-4_)*K*_9_(*k*_+11_)(*k*_-13_)(*k*_+15_) 
         _+_ *K*_1_(*k*_+10_)(*k*_+3_)(*k*_-8_)(*k*_+7_)(*k*_-4_)*K*_9_(*k*_+11_)(*k*_-13_)(*k*_-14_) 
         _+_ *K*_1_(*k*_+10_)(*k*_+3_)(*k*_-8_)(*k*_+7_)(*k*_-4_)*K*_9_(*k*_+11_)(*k*_+12_)(*k*_+15_) 
         _+_ *K*_1_(*k*_+10_)(*k*_+3_)(*k*_-8_)(*k*_+7_)(*k*_-4_)*K*_9_(*k*_+11_)(*k*_+12_)(*k*_-14_) 
         _+_ *K*_1_(*k*_+10_)(*k*_+3_)(*k*_-8_)(*k*_-6_)(*k*_+5_)*K*_9_(*k*_+11_)(*k*_-13_)(*k*_+15_) 
         _+_ *K*_1_(*k*_+10_)(*k*_+3_)(*k*_-8_)(*k*_-6_)(*k*_+5_)*K*_9_(*k*_+11_)(*k*_-13_)(*k*_-14_) 
         _+_ *K*_1_(*k*_+10_)(*k*_+3_)(*k*_-8_)(*k*_-6_)(*k*_+5_)*K*_9_(*k*_+11_)(*k*_+12_)(*k*_+15_) 
         _+_ *K*_1_(*k*_+10_)(*k*_+3_)(*k*_-8_)(*k*_-6_)(*k*_+5_)*K*_9_(*k*_+11_)(*k*_+12_)(*k*_-14_) 
         _+_ *K*_1_(*k*_+10_)(*k*_+3_)(*k*_-8_)(*k*_-6_)(*k*_-4_)*K*_9_(*k*_+11_)(*k*_-13_)(*k*_+15_) 
         _+_ *K*_1_(*k*_+10_)(*k*_+3_)(*k*_-8_)(*k*_-6_)(*k*_-4_)*K*_9_(*k*_+11_)(*k*_-13_)(*k*_-14_) 
         _+_ *K*_1_(*k*_+10_)(*k*_+3_)(*k*_-8_)(*k*_-6_)(*k*_-4_)*K*_9_(*k*_+11_)(*k*_+12_)(*k*_+15_) 
         _+_ *K*_1_(*k*_+10_)(*k*_+3_)(*k*_-8_)(*k*_-6_)(*k*_-4_)*K*_9_(*k*_+11_)(*k*_+12_)(*k*_-14_)}
β_2_ = {*K*_1_(*k*_+2_)(*k*_+3_)(*k*_-8_)(*k*_+7_)(*k*_+5_)*K*_9_(*k*_-10_)(*k*_-13_)(*k*_+15_) 
         _+_ *K*_1_(*k*_+2_)(*k*_+3_)(*k*_-8_)(*k*_+7_)(*k*_+5_)*K*_9_(*k*_-10_)(*k*_-13_)(*k*_-14_) 
         _+_ *K*_1_(*k*_+2_)(*k*_+3_)(*k*_-8_)(*k*_+7_)(*k*_+5_)*K*_9_(*k*_-10_)(*k*_+12_)(*k*_+15_) 
         _+_ *K*_1_(*k*_+2_)(*k*_+3_)(*k*_-8_)(*k*_+7_)(*k*_+5_)*K*_9_(*k*_-10_)(*k*_+12_)(*k*_-14_) 
         _+_ *K*_1_(*k*_+2_)(*k*_+3_)(*k*_-8_)(*k*_+7_)(*k*_+5_)*K*_9_(*k*_+11_)(*k*_-13_)(*k*_+15_) 
         _+_ *K*_1_(*k*_+2_)(*k*_+3_)(*k*_-8_)(*k*_+7_)(*k*_+5_)*K*_9_(*k*_+11_)(*k*_-13_)(*k*_-14_) 
         _+_ *K*_1_(*k*_+2_)(*k*_+3_)(*k*_-8_)(*k*_+7_)(*k*_+5_)*K*_9_(*k*_+11_)(*k*_+12_)(*k*_+15_) 
         _+_ *K*_1_(*k*_+2_)(*k*_+3_)(*k*_-8_)(*k*_+7_)(*k*_+5_)*K*_9_(*k*_+11_)(*k*_+12_)(*k*_-14_) 
         _+_ *K*_1_(*k*_+2_)(*k*_+3_)(*k*_-8_)(*k*_+7_)(*k*_-4_)*K*_9_(*k*_-10_)(*k*_-13_)(*k*_+15_) 
         _+_ *K*_1_(*k*_+2_)(*k*_+3_)(*k*_-8_)(*k*_+7_)(*k*_-4_)*K*_9_(*k*_-10_)(*k*_-13_)(*k*_-14_) 
         _+_ *K*_1_(*k*_+2_)(*k*_+3_)(*k*_-8_)(*k*_+7_)(*k*_-4_)*K*_9_(*k*_-10_)(*k*_+12_)(*k*_+15_) 
         _+_ *K*_1_(*k*_+2_)(*k*_+3_)(*k*_-8_)(*k*_+7_)(*k*_-4_)*K*_9_(*k*_-10_)(*k*_+12_)(*k*_-14_) 
         _+_ *K*_1_(*k*_+2_)(*k*_+3_)(*k*_-8_)(*k*_+7_)(*k*_-4_)*K*_9_(*k*_+11_)(*k*_-13_)(*k*_+15_) 
         _+_ *K*_1_(*k*_+2_)(*k*_+3_)(*k*_-8_)(*k*_+7_)(*k*_-4_)*K*_9_(*k*_+11_)(*k*_-13_)(*k*_-14_) 
         _+_ *K*_1_(*k*_+2_)(*k*_+3_)(*k*_-8_)(*k*_+7_)(*k*_-4_)*K*_9_(*k*_+11_)(*k*_+12_)(*k*_+15_) 
         _+_ *K*_1_(*k*_+2_)(*k*_+3_)(*k*_-8_)(*k*_+7_)(*k*_-4_)*K*_9_(*k*_+11_)(*k*_+12_)(*k*_-14_) 
         _+_ *K*_1_(*k*_+2_)(*k*_+3_)(*k*_-8_)(*k*_-6_)(*k*_+5_)*K*_9_(*k*_-10_)(*k*_-13_)(*k*_+15_) 
         _+_ *K*_1_(*k*_+2_)(*k*_+3_)(*k*_-8_)(*k*_-6_)(*k*_+5_)*K*_9_(*k*_-10_)(*k*_-13_)(*k*_-14_) 
         _+_ *K*_1_(*k*_+2_)(*k*_+3_)(*k*_-8_)(*k*_-6_)(*k*_+5_)*K*_9_(*k*_-10_)(*k*_+12_)(*k*_+15_) 
         _+_ *K*_1_(*k*_+2_)(*k*_+3_)(*k*_-8_)(*k*_-6_)(*k*_+5_)*K*_9_(*k*_-10_)(*k*_+12_)(*k*_-14_) 
         _+_ *K*_1_(*k*_+2_)(*k*_+3_)(*k*_-8_)(*k*_-6_)(*k*_+5_)*K*_9_(*k*_+11_)(*k*_-13_)(*k*_+15_) 
         _+_ *K*_1_(*k*_+2_)(*k*_+3_)(*k*_-8_)(*k*_-6_)(*k*_+5_)*K*_9_(*k*_+11_)(*k*_-13_)(*k*_-14_) 
         _+_ *K*_1_(*k*_+2_)(*k*_+3_)(*k*_-8_)(*k*_-6_)(*k*_+5_)*K*_9_(*k*_+11_)(*k*_+12_)(*k*_+15_) 
         _+_ *K*_1_(*k*_+2_)(*k*_+3_)(*k*_-8_)(*k*_-6_)(*k*_+5_)*K*_9_(*k*_+11_)(*k*_+12_)(*k*_-14_) 
         _+_ *K*_1_(*k*_+2_)(*k*_+3_)(*k*_-8_)(*k*_-6_)(*k*_-4_)*K*_9_(*k*_-10_)(*k*_-13_)(*k*_+15_) 
         _+_ *K*_1_(*k*_+2_)(*k*_+3_)(*k*_-8_)(*k*_-6_)(*k*_-4_)*K*_9_(*k*_-10_)(*k*_-13_)(*k*_-14_) 
         _+_ *K*_1_(*k*_+2_)(*k*_+3_)(*k*_-8_)(*k*_-6_)(*k*_-4_)*K*_9_(*k*_-10_)(*k*_+12_)(*k*_+15_) 
         _+_ *K*_1_(*k*_+2_)(*k*_+3_)(*k*_-8_)(*k*_-6_)(*k*_-4_)*K*_9_(*k*_-10_)(*k*_+12_)(*k*_-14_) 
         _+_ *K*_1_(*k*_+2_)(*k*_+3_)(*k*_-8_)(*k*_-6_)(*k*_-4_)*K*_9_(*k*_+11_)(*k*_-13_)(*k*_+15_) 
         _+_ *K*_1_(*k*_+2_)(*k*_+3_)(*k*_-8_)(*k*_-6_)(*k*_-4_)*K*_9_(*k*_+11_)(*k*_-13_)(*k*_-14_) 
         _+_ *K*_1_(*k*_+2_)(*k*_+3_)(*k*_-8_)(*k*_-6_)(*k*_-4_)*K*_9_(*k*_+11_)(*k*_+12_)(*k*_+15_) 
         _+_ *K*_1_(*k*_+2_)(*k*_+3_)(*k*_-8_)(*k*_-6_)(*k*_-4_)*K*_9_(*k*_+11_)(*k*_+12_)(*k*_-14_)}
β_3_ = {*K*_1_(*k*_+10_)(*k*_-2_)(*k*_+14_)(*k*_+7_)(*k*_+5_)*K*_9_(*k*_+11_)(*k*_-13_)(*k*_+15_) 
         _+_ *K*_1_(*k*_+10_)(*k*_-2_)(*k*_+14_)(*k*_+7_)(*k*_+5_)*K*_9_(*k*_+11_)(*k*_+12_)(*k*_+15_) 
         _+_ *K*_1_(*k*_+10_)(*k*_-2_)(*k*_+14_)(*k*_+7_)(*k*_-4_)*K*_9_(*k*_+11_)(*k*_-13_)(*k*_+15_) 
         _+_ *K*_1_(*k*_+10_)(*k*_-2_)(*k*_+14_)(*k*_+7_)(*k*_-4_)*K*_9_(*k*_+11_)(*k*_+12_)(*k*_+15_) 
         _+_ *K*_1_(*k*_+10_)(*k*_-2_)(*k*_+14_)(*k*_-6_)(*k*_+5_)*K*_9_(*k*_+11_)(*k*_-13_)(*k*_+15_) 
         _+_ *K*_1_(*k*_+10_)(*k*_-2_)(*k*_+14_)(*k*_-6_)(*k*_+5_)*K*_9_(*k*_+11_)(*k*_+12_)(*k*_+15_) 
         _+_ *K*_1_(*k*_+10_)(*k*_-2_)(*k*_+14_)(*k*_-6_)(*k*_-4_)*K*_9_(*k*_+11_)(*k*_-13_)(*k*_+15_) 
         _+_ *K*_1_(*k*_+10_)(*k*_-2_)(*k*_+14_)(*k*_-6_)(*k*_-4_)*K*_9_(*k*_+11_)(*k*_+12_)(*k*_+15_) 
         _+_ *K*_1_(*k*_+10_)(*k*_+3_)(*k*_+14_)(*k*_+7_)(*k*_+5_)*K*_9_(*k*_+11_)(*k*_-13_)(*k*_+15_) 
         _+_ *K*_1_(*k*_+10_)(*k*_+3_)(*k*_+14_)(*k*_+7_)(*k*_+5_)*K*_9_(*k*_+11_)(*k*_+12_)(*k*_+15_) 
         _+_ *K*_1_(*k*_+10_)(*k*_+3_)(*k*_+14_)(*k*_+7_)(*k*_-4_)*K*_9_(*k*_+11_)(*k*_-13_)(*k*_+15_) 
         _+_ *K*_1_(*k*_+10_)(*k*_+3_)(*k*_+14_)(*k*_+7_)(*k*_-4_)*K*_9_(*k*_+11_)(*k*_+12_)(*k*_+15_) 
         _+_ *K*_1_(*k*_+10_)(*k*_+3_)(*k*_+14_)(*k*_-6_)(*k*_+5_)*K*_9_(*k*_+11_)(*k*_-13_)(*k*_+15_) 
         _+_ *K*_1_(*k*_+10_)(*k*_+3_)(*k*_+14_)(*k*_-6_)(*k*_+5_)*K*_9_(*k*_+11_)(*k*_+12_)(*k*_+15_) 
         _+_ *K*_1_(*k*_+10_)(*k*_+3_)(*k*_+14_)(*k*_-6_)(*k*_-4_)*K*_9_(*k*_+11_)(*k*_-13_)(*k*_+15_) 
         _+_ *K*_1_(*k*_+10_)(*k*_+3_)(*k*_+14_)(*k*_-6_)(*k*_-4_)*K*_9_(*k*_+11_)(*k*_+12_)(*k*_+15_)}
β_4_ = {*K*_1_(*k*_+10_)(*k*_-2_)(*k*_+4_)(*k*_+7_)(*k*_+5_)*K*_9_(*k*_+11_)(*k*_-13_)(*k*_+15_) 
         _+_ *K*_1_(*k*_+10_)(*k*_-2_)(*k*_+4_)(*k*_+7_)(*k*_+5_)*K*_9_(*k*_+11_)(*k*_-13_)(*k*_-14_) 
         _+_ *K*_1_(*k*_+10_)(*k*_-2_)(*k*_+4_)(*k*_+7_)(*k*_+5_)*K*_9_(*k*_+11_)(*k*_+12_)(*k*_+15_) 
         _+_ *K*_1_(*k*_+10_)(*k*_-2_)(*k*_+4_)(*k*_+7_)(*k*_+5_)*K*_9_(*k*_+11_)(*k*_+12_)(*k*_-14_) 
         _+_ *K*_1_(*k*_+10_)(*k*_-2_)(*k*_+4_)(*k*_-6_)(*k*_+5_)*K*_9_(*k*_+11_)(*k*_-13_)(*k*_+15_) 
         _+_ *K*_1_(*k*_+10_)(*k*_-2_)(*k*_+4_)(*k*_-6_)(*k*_+5_)*K*_9_(*k*_+11_)(*k*_-13_)(*k*_-14_) 
         _+_ *K*_1_(*k*_+10_)(*k*_-2_)(*k*_+4_)(*k*_-6_)(*k*_+5_)*K*_9_(*k*_+11_)(*k*_+12_)(*k*_+15_) 
         _+_ *K*_1_(*k*_+10_)(*k*_-2_)(*k*_+4_)(*k*_-6_)(*k*_+5_)*K*_9_(*k*_+11_)(*k*_+12_)(*k*_-14_) 
         _+_ *K*_1_(*k*_+10_)(*k*_+3_)(*k*_+4_)(*k*_+7_)(*k*_+5_)*K*_9_(*k*_+11_)(*k*_-13_)(*k*_+15_) 
         _+_ *K*_1_(*k*_+10_)(*k*_+3_)(*k*_+4_)(*k*_+7_)(*k*_+5_)*K*_9_(*k*_+11_)(*k*_-13_)(*k*_-14_) 
         _+_ *K*_1_(*k*_+10_)(*k*_+3_)(*k*_+4_)(*k*_+7_)(*k*_+5_)*K*_9_(*k*_+11_)(*k*_+12_)(*k*_+15_) 
         _+_ *K*_1_(*k*_+10_)(*k*_+3_)(*k*_+4_)(*k*_+7_)(*k*_+5_)*K*_9_(*k*_+11_)(*k*_+12_)(*k*_-14_) 
         _+_ *K*_1_(*k*_+10_)(*k*_+3_)(*k*_+4_)(*k*_-6_)(*k*_+5_)*K*_9_(*k*_+11_)(*k*_-13_)(*k*_+15_) 
         _+_ *K*_1_(*k*_+10_)(*k*_+3_)(*k*_+4_)(*k*_-6_)(*k*_+5_)*K*_9_(*k*_+11_)(*k*_-13_)(*k*_-14_) 
         _+_ *K*_1_(*k*_+10_)(*k*_+3_)(*k*_+4_)(*k*_-6_)(*k*_+5_)*K*_9_(*k*_+11_)(*k*_+12_)(*k*_+15_) 
         _+_ *K*_1_(*k*_+10_)(*k*_+3_)(*k*_+4_)(*k*_-6_)(*k*_+5_)*K*_9_(*k*_+11_)(*k*_+12_)(*k*_-14_)}
β_5_ = {*K*_1_(*k*_+2_)(*k*_+3_)(*k*_+14_)(*k*_+7_)(*k*_+5_)*K*_9_(*k*_-10_)(*k*_-13_)(*k*_+15_) 
         _+_ *K*_1_(*k*_+2_)(*k*_+3_)(*k*_+14_)(*k*_+7_)(*k*_+5_)*K*_9_(*k*_-10_)(*k*_+12_)(*k*_+15_) 
         _+_ *K*_1_(*k*_+2_)(*k*_+3_)(*k*_+14_)(*k*_+7_)(*k*_+5_)*K*_9_(*k*_+11_)(*k*_-13_)(*k*_+15_) 
         _+_ *K*_1_(*k*_+2_)(*k*_+3_)(*k*_+14_)(*k*_+7_)(*k*_+5_)*K*_9_(*k*_+11_)(*k*_+12_)(*k*_+15_) 
         _+_ *K*_1_(*k*_+2_)(*k*_+3_)(*k*_+14_)(*k*_+7_)(*k*_-4_)*K*_9_(*k*_-10_)(*k*_-13_)(*k*_+15_) 
         _+_ *K*_1_(*k*_+2_)(*k*_+3_)(*k*_+14_)(*k*_+7_)(*k*_-4_)*K*_9_(*k*_-10_)(*k*_+12_)(*k*_+15_) 
         _+_ *K*_1_(*k*_+2_)(*k*_+3_)(*k*_+14_)(*k*_+7_)(*k*_-4_)*K*_9_(*k*_+11_)(*k*_-13_)(*k*_+15_) 
         _+_ *K*_1_(*k*_+2_)(*k*_+3_)(*k*_+14_)(*k*_+7_)(*k*_-4_)*K*_9_(*k*_+11_)(*k*_+12_)(*k*_+15_) 
         _+_ *K*_1_(*k*_+2_)(*k*_+3_)(*k*_+14_)(*k*_-6_)(*k*_+5_)*K*_9_(*k*_-10_)(*k*_-13_)(*k*_+15_) 
         _+_ *K*_1_(*k*_+2_)(*k*_+3_)(*k*_+14_)(*k*_-6_)(*k*_+5_)*K*_9_(*k*_-10_)(*k*_+12_)(*k*_+15_) 
         _+_ *K*_1_(*k*_+2_)(*k*_+3_)(*k*_+14_)(*k*_-6_)(*k*_+5_)*K*_9_(*k*_+11_)(*k*_-13_)(*k*_+15_) 
         _+_ *K*_1_(*k*_+2_)(*k*_+3_)(*k*_+14_)(*k*_-6_)(*k*_+5_)*K*_9_(*k*_+11_)(*k*_+12_)(*k*_+15_) 
         _+_ *K*_1_(*k*_+2_)(*k*_+3_)(*k*_+14_)(*k*_-6_)(*k*_-4_)*K*_9_(*k*_-10_)(*k*_-13_)(*k*_+15_) 
         _+_ *K*_1_(*k*_+2_)(*k*_+3_)(*k*_+14_)(*k*_-6_)(*k*_-4_)*K*_9_(*k*_-10_)(*k*_+12_)(*k*_+15_) 
         _+_ *K*_1_(*k*_+2_)(*k*_+3_)(*k*_+14_)(*k*_-6_)(*k*_-4_)*K*_9_(*k*_+11_)(*k*_-13_)(*k*_+15_) 
         _+_ *K*_1_(*k*_+2_)(*k*_+3_)(*k*_+14_)(*k*_-6_)(*k*_-4_)*K*_9_(*k*_+11_)(*k*_+12_)(*k*_+15_) 
         _+_ *K*_1_(*k*_+10_)(*k*_-2_)(*k*_+6_)(*k*_+7_)(*k*_+5_)*K*_9_(*k*_+11_)(*k*_-13_)(*k*_+15_) 
         _+_ *K*_1_(*k*_+10_)(*k*_-2_)(*k*_+6_)(*k*_+7_)(*k*_+5_)*K*_9_(*k*_+11_)(*k*_-13_)(*k*_-14_) 
         _+_ *K*_1_(*k*_+10_)(*k*_-2_)(*k*_+6_)(*k*_+7_)(*k*_+5_)*K*_9_(*k*_+11_)(*k*_+12_)(*k*_+15_) 
         _+_ *K*_1_(*k*_+10_)(*k*_-2_)(*k*_+6_)(*k*_+7_)(*k*_+5_)*K*_9_(*k*_+11_)(*k*_+12_)(*k*_-14_) 
         _+_ *K*_1_(*k*_+10_)(*k*_-2_)(*k*_+6_)(*k*_+7_)(*k*_-4_)*K*_9_(*k*_+11_)(*k*_-13_)(*k*_+15_) 
         _+_ *K*_1_(*k*_+10_)(*k*_-2_)(*k*_+6_)(*k*_+7_)(*k*_-4_)*K*_9_(*k*_+11_)(*k*_-13_)(*k*_-14_) 
         _+_ *K*_1_(*k*_+10_)(*k*_-2_)(*k*_+6_)(*k*_+7_)(*k*_-4_)*K*_9_(*k*_+11_)(*k*_+12_)(*k*_+15_) 
         _+_ *K*_1_(*k*_+10_)(*k*_-2_)(*k*_+6_)(*k*_+7_)(*k*_-4_)*K*_9_(*k*_+11_)(*k*_+12_)(*k*_-14_) 
         _+_ *K*_1_(*k*_+10_)(*k*_+3_)(*k*_+6_)(*k*_+7_)(*k*_+5_)*K*_9_(*k*_+11_)(*k*_-13_)(*k*_+15_) 
         _+_ *K*_1_(*k*_+10_)(*k*_+3_)(*k*_+6_)(*k*_+7_)(*k*_+5_)*K*_9_(*k*_+11_)(*k*_-13_)(*k*_-14_) 
         _+_ *K*_1_(*k*_+10_)(*k*_+3_)(*k*_+6_)(*k*_+7_)(*k*_+5_)*K*_9_(*k*_+11_)(*k*_+12_)(*k*_+15_) 
         _+_ *K*_1_(*k*_+10_)(*k*_+3_)(*k*_+6_)(*k*_+7_)(*k*_+5_)*K*_9_(*k*_+11_)(*k*_+12_)(*k*_-14_) 
         _+_ *K*_1_(*k*_+10_)(*k*_+3_)(*k*_+6_)(*k*_+7_)(*k*_-4_)*K*_9_(*k*_+11_)(*k*_-13_)(*k*_+15_) 
         _+_ *K*_1_(*k*_+10_)(*k*_+3_)(*k*_+6_)(*k*_+7_)(*k*_-4_)*K*_9_(*k*_+11_)(*k*_-13_)(*k*_-14_) 
         _+_ *K*_1_(*k*_+10_)(*k*_+3_)(*k*_+6_)(*k*_+7_)(*k*_-4_)*K*_9_(*k*_+11_)(*k*_+12_)(*k*_+15_) 
         _+_ *K*_1_(*k*_+10_)(*k*_+3_)(*k*_+6_)(*k*_+7_)(*k*_-4_)*K*_9_(*k*_+11_)(*k*_+12_)(*k*_-14_)}
β_6_ = {*K*_1_(*k*_+2_)(*k*_+3_)(*k*_+4_)(*k*_+7_)(*k*_+5_)*K*_9_(*k*_-10_)(*k*_-13_)(*k*_+15_) 
         _+_ *K*_1_(*k*_+2_)(*k*_+3_)(*k*_+4_)(*k*_+7_)(*k*_+5_)*K*_9_(*k*_-10_)(*k*_-13_)(*k*_-14_) 
         _+_ *K*_1_(*k*_+2_)(*k*_+3_)(*k*_+4_)(*k*_+7_)(*k*_+5_)*K*_9_(*k*_-10_)(*k*_+12_)(*k*_+15_) 
         _+_ *K*_1_(*k*_+2_)(*k*_+3_)(*k*_+4_)(*k*_+7_)(*k*_+5_)*K*_9_(*k*_-10_)(*k*_+12_)(*k*_-14_) 
         _+_ *K*_1_(*k*_+2_)(*k*_+3_)(*k*_+4_)(*k*_+7_)(*k*_+5_)*K*_9_(*k*_+11_)(*k*_-13_)(*k*_+15_) 
         _+_ *K*_1_(*k*_+2_)(*k*_+3_)(*k*_+4_)(*k*_+7_)(*k*_+5_)*K*_9_(*k*_+11_)(*k*_-13_)(*k*_-14_) 
         _+_ *K*_1_(*k*_+2_)(*k*_+3_)(*k*_+4_)(*k*_+7_)(*k*_+5_)*K*_9_(*k*_+11_)(*k*_+12_)(*k*_+15_) 
         _+_ *K*_1_(*k*_+2_)(*k*_+3_)(*k*_+4_)(*k*_+7_)(*k*_+5_)*K*_9_(*k*_+11_)(*k*_+12_)(*k*_-14_) 
         _+_ *K*_1_(*k*_+2_)(*k*_+3_)(*k*_+4_)(*k*_-6_)(*k*_+5_)*K*_9_(*k*_-10_)(*k*_-13_)(*k*_+15_) 
         _+_ *K*_1_(*k*_+2_)(*k*_+3_)(*k*_+4_)(*k*_-6_)(*k*_+5_)*K*_9_(*k*_-10_)(*k*_-13_)(*k*_-14_) 
         _+_ *K*_1_(*k*_+2_)(*k*_+3_)(*k*_+4_)(*k*_-6_)(*k*_+5_)*K*_9_(*k*_-10_)(*k*_+12_)(*k*_+15_) 
         _+_ *K*_1_(*k*_+2_)(*k*_+3_)(*k*_+4_)(*k*_-6_)(*k*_+5_)*K*_9_(*k*_-10_)(*k*_+12_)(*k*_-14_) 
         _+_ *K*_1_(*k*_+2_)(*k*_+3_)(*k*_+4_)(*k*_-6_)(*k*_+5_)*K*_9_(*k*_+11_)(*k*_-13_)(*k*_+15_) 
         _+_ *K*_1_(*k*_+2_)(*k*_+3_)(*k*_+4_)(*k*_-6_)(*k*_+5_)*K*_9_(*k*_+11_)(*k*_-13_)(*k*_-14_) 
         _+_ *K*_1_(*k*_+2_)(*k*_+3_)(*k*_+4_)(*k*_-6_)(*k*_+5_)*K*_9_(*k*_+11_)(*k*_+12_)(*k*_+15_) 
         _+_ *K*_1_(*k*_+2_)(*k*_+3_)(*k*_+4_)(*k*_-6_)(*k*_+5_)*K*_9_(*k*_+11_)(*k*_+12_)(*k*_-14_)}
β_7_ = {*K*_1_(*k*_+2_)(*k*_+3_)(*k*_+6_)(*k*_+7_)(*k*_+5_)*K*_9_(*k*_-10_)(*k*_-13_)(*k*_+15_) 
         _+_ *K*_1_(*k*_+2_)(*k*_+3_)(*k*_+6_)(*k*_+7_)(*k*_+5_)*K*_9_(*k*_-10_)(*k*_-13_)(*k*_-14_) 
         _+_ *K*_1_(*k*_+2_)(*k*_+3_)(*k*_+6_)(*k*_+7_)(*k*_+5_)*K*_9_(*k*_-10_)(*k*_+12_)(*k*_+15_) 
         _+_ *K*_1_(*k*_+2_)(*k*_+3_)(*k*_+6_)(*k*_+7_)(*k*_+5_)*K*_9_(*k*_-10_)(*k*_+12_)(*k*_-14_) 
         _+_ *K*_1_(*k*_+2_)(*k*_+3_)(*k*_+6_)(*k*_+7_)(*k*_+5_)*K*_9_(*k*_+11_)(*k*_-13_)(*k*_+15_) 
         _+_ *K*_1_(*k*_+2_)(*k*_+3_)(*k*_+6_)(*k*_+7_)(*k*_+5_)*K*_9_(*k*_+11_)(*k*_-13_)(*k*_-14_) 
         _+_ *K*_1_(*k*_+2_)(*k*_+3_)(*k*_+6_)(*k*_+7_)(*k*_+5_)*K*_9_(*k*_+11_)(*k*_+12_)(*k*_+15_) 
         _+_ *K*_1_(*k*_+2_)(*k*_+3_)(*k*_+6_)(*k*_+7_)(*k*_+5_)*K*_9_(*k*_+11_)(*k*_+12_)(*k*_-14_) 
         _+_ *K*_1_(*k*_+2_)(*k*_+3_)(*k*_+6_)(*k*_+7_)(*k*_-4_)*K*_9_(*k*_-10_)(*k*_-13_)(*k*_+15_) 
         _+_ *K*_1_(*k*_+2_)(*k*_+3_)(*k*_+6_)(*k*_+7_)(*k*_-4_)*K*_9_(*k*_-10_)(*k*_-13_)(*k*_-14_) 
         _+_ *K*_1_(*k*_+2_)(*k*_+3_)(*k*_+6_)(*k*_+7_)(*k*_-4_)*K*_9_(*k*_-10_)(*k*_+12_)(*k*_+15_) 
         _+_ *K*_1_(*k*_+2_)(*k*_+3_)(*k*_+6_)(*k*_+7_)(*k*_-4_)*K*_9_(*k*_-10_)(*k*_+12_)(*k*_-14_) 
         _+_ *K*_1_(*k*_+2_)(*k*_+3_)(*k*_+6_)(*k*_+7_)(*k*_-4_)*K*_9_(*k*_+11_)(*k*_-13_)(*k*_+15_) 
         _+_ *K*_1_(*k*_+2_)(*k*_+3_)(*k*_+6_)(*k*_+7_)(*k*_-4_)*K*_9_(*k*_+11_)(*k*_-13_)(*k*_-14_) 
         _+_ *K*_1_(*k*_+2_)(*k*_+3_)(*k*_+6_)(*k*_+7_)(*k*_-4_)*K*_9_(*k*_+11_)(*k*_+12_)(*k*_+15_) 
         _+_ *K*_1_(*k*_+2_)(*k*_+3_)(*k*_+6_)(*k*_+7_)(*k*_-4_)*K*_9_(*k*_+11_)(*k*_+12_)(*k*_-14_)}
β_8_ = {*K*_1_(*k*_-2_)(*k*_+8_)(*k*_+14_)(*k*_+7_)(*k*_+5_)*K*_9_(*k*_-10_)(*k*_-13_)(*k*_+15_) 
         _+_ *K*_1_(*k*_-2_)(*k*_+8_)(*k*_+14_)(*k*_+7_)(*k*_+5_)*K*_9_(*k*_-10_)(*k*_+12_)(*k*_+15_) 
         _+_ *K*_1_(*k*_-2_)(*k*_+8_)(*k*_+14_)(*k*_+7_)(*k*_+5_)*K*_9_(*k*_+11_)(*k*_-13_)(*k*_+15_) 
         _+_ *K*_1_(*k*_-2_)(*k*_+8_)(*k*_+14_)(*k*_+7_)(*k*_+5_)*K*_9_(*k*_+11_)(*k*_+12_)(*k*_+15_) 
         _+_ *K*_1_(*k*_-2_)(*k*_+8_)(*k*_+14_)(*k*_+7_)(*k*_-4_)*K*_9_(*k*_-10_)(*k*_-13_)(*k*_+15_) 
         _+_ *K*_1_(*k*_-2_)(*k*_+8_)(*k*_+14_)(*k*_+7_)(*k*_-4_)*K*_9_(*k*_-10_)(*k*_+12_)(*k*_+15_) 
         _+_ *K*_1_(*k*_-2_)(*k*_+8_)(*k*_+14_)(*k*_+7_)(*k*_-4_)*K*_9_(*k*_+11_)(*k*_-13_)(*k*_+15_) 
         _+_ *K*_1_(*k*_-2_)(*k*_+8_)(*k*_+14_)(*k*_+7_)(*k*_-4_)*K*_9_(*k*_+11_)(*k*_+12_)(*k*_+15_) 
         _+_ *K*_1_(*k*_-2_)(*k*_+8_)(*k*_+14_)(*k*_-6_)(*k*_+5_)*K*_9_(*k*_-10_)(*k*_-13_)(*k*_+15_) 
         _+_ *K*_1_(*k*_-2_)(*k*_+8_)(*k*_+14_)(*k*_-6_)(*k*_+5_)*K*_9_(*k*_-10_)(*k*_+12_)(*k*_+15_) 
         _+_ *K*_1_(*k*_-2_)(*k*_+8_)(*k*_+14_)(*k*_-6_)(*k*_+5_)*K*_9_(*k*_+11_)(*k*_-13_)(*k*_+15_) 
         _+_ *K*_1_(*k*_-2_)(*k*_+8_)(*k*_+14_)(*k*_-6_)(*k*_+5_)*K*_9_(*k*_+11_)(*k*_+12_)(*k*_+15_) 
         _+_ *K*_1_(*k*_-2_)(*k*_+8_)(*k*_+14_)(*k*_-6_)(*k*_-4_)*K*_9_(*k*_-10_)(*k*_-13_)(*k*_+15_) 
         _+_ *K*_1_(*k*_-2_)(*k*_+8_)(*k*_+14_)(*k*_-6_)(*k*_-4_)*K*_9_(*k*_-10_)(*k*_+12_)(*k*_+15_) 
         _+_ *K*_1_(*k*_-2_)(*k*_+8_)(*k*_+14_)(*k*_-6_)(*k*_-4_)*K*_9_(*k*_+11_)(*k*_-13_)(*k*_+15_) 
         _+_ *K*_1_(*k*_-2_)(*k*_+8_)(*k*_+14_)(*k*_-6_)(*k*_-4_)*K*_9_(*k*_+11_)(*k*_+12_)(*k*_+15_) 
         _+_ *K*_1_(*k*_+3_)(*k*_+8_)(*k*_+14_)(*k*_+7_)(*k*_+5_)*K*_9_(*k*_-10_)(*k*_-13_)(*k*_+15_) 
         _+_ *K*_1_(*k*_+3_)(*k*_+8_)(*k*_+14_)(*k*_+7_)(*k*_+5_)*K*_9_(*k*_-10_)(*k*_+12_)(*k*_+15_) 
         _+_ *K*_1_(*k*_+3_)(*k*_+8_)(*k*_+14_)(*k*_+7_)(*k*_+5_)*K*_9_(*k*_+11_)(*k*_-13_)(*k*_+15_) 
         _+_ *K*_1_(*k*_+3_)(*k*_+8_)(*k*_+14_)(*k*_+7_)(*k*_+5_)*K*_9_(*k*_+11_)(*k*_+12_)(*k*_+15_) 
         _+_ *K*_1_(*k*_+3_)(*k*_+8_)(*k*_+14_)(*k*_+7_)(*k*_-4_)*K*_9_(*k*_-10_)(*k*_-13_)(*k*_+15_) 
         _+_ *K*_1_(*k*_+3_)(*k*_+8_)(*k*_+14_)(*k*_+7_)(*k*_-4_)*K*_9_(*k*_-10_)(*k*_+12_)(*k*_+15_) 
         _+_ *K*_1_(*k*_+3_)(*k*_+8_)(*k*_+14_)(*k*_+7_)(*k*_-4_)*K*_9_(*k*_+11_)(*k*_-13_)(*k*_+15_) 
         _+_ *K*_1_(*k*_+3_)(*k*_+8_)(*k*_+14_)(*k*_+7_)(*k*_-4_)*K*_9_(*k*_+11_)(*k*_+12_)(*k*_+15_) 
         _+_ *K*_1_(*k*_+3_)(*k*_+8_)(*k*_+14_)(*k*_-6_)(*k*_+5_)*K*_9_(*k*_-10_)(*k*_-13_)(*k*_+15_) 
         _+_ *K*_1_(*k*_+3_)(*k*_+8_)(*k*_+14_)(*k*_-6_)(*k*_+5_)*K*_9_(*k*_-10_)(*k*_+12_)(*k*_+15_) 
         _+_ *K*_1_(*k*_+3_)(*k*_+8_)(*k*_+14_)(*k*_-6_)(*k*_+5_)*K*_9_(*k*_+11_)(*k*_-13_)(*k*_+15_) 
         _+_ *K*_1_(*k*_+3_)(*k*_+8_)(*k*_+14_)(*k*_-6_)(*k*_+5_)*K*_9_(*k*_+11_)(*k*_+12_)(*k*_+15_) 
         _+_ *K*_1_(*k*_+3_)(*k*_+8_)(*k*_+14_)(*k*_-6_)(*k*_-4_)*K*_9_(*k*_-10_)(*k*_-13_)(*k*_+15_) 
         _+_ *K*_1_(*k*_+3_)(*k*_+8_)(*k*_+14_)(*k*_-6_)(*k*_-4_)*K*_9_(*k*_-10_)(*k*_+12_)(*k*_+15_) 
         _+_ *K*_1_(*k*_+3_)(*k*_+8_)(*k*_+14_)(*k*_-6_)(*k*_-4_)*K*_9_(*k*_+11_)(*k*_-13_)(*k*_+15_) 
         _+_ *K*_1_(*k*_+3_)(*k*_+8_)(*k*_+14_)(*k*_-6_)(*k*_-4_)*K*_9_(*k*_+11_)(*k*_+12_)(*k*_+15_) 
         _+_ *K*_1_(*k*_+10_)(*k*_-2_)(*k*_+8_)(*k*_+7_)(*k*_+5_)*K*_9_(*k*_+11_)(*k*_-13_)(*k*_+15_) 
         _+_ *K*_1_(*k*_+10_)(*k*_-2_)(*k*_+8_)(*k*_+7_)(*k*_+5_)*K*_9_(*k*_+11_)(*k*_-13_)(*k*_-14_) 
         _+_ *K*_1_(*k*_+10_)(*k*_-2_)(*k*_+8_)(*k*_+7_)(*k*_+5_)*K*_9_(*k*_+11_)(*k*_+12_)(*k*_+15_) 
         _+_ *K*_1_(*k*_+10_)(*k*_-2_)(*k*_+8_)(*k*_+7_)(*k*_+5_)*K*_9_(*k*_+11_)(*k*_+12_)(*k*_-14_) 
         _+_ *K*_1_(*k*_+10_)(*k*_-2_)(*k*_+8_)(*k*_+7_)(*k*_-4_)*K*_9_(*k*_+11_)(*k*_-13_)(*k*_+15_) 
         _+_ *K*_1_(*k*_+10_)(*k*_-2_)(*k*_+8_)(*k*_+7_)(*k*_-4_)*K*_9_(*k*_+11_)(*k*_-13_)(*k*_-14_) 
         _+_ *K*_1_(*k*_+10_)(*k*_-2_)(*k*_+8_)(*k*_+7_)(*k*_-4_)*K*_9_(*k*_+11_)(*k*_+12_)(*k*_+15_) 
         _+_ *K*_1_(*k*_+10_)(*k*_-2_)(*k*_+8_)(*k*_+7_)(*k*_-4_)*K*_9_(*k*_+11_)(*k*_+12_)(*k*_-14_) 
         _+_ *K*_1_(*k*_+10_)(*k*_-2_)(*k*_+8_)(*k*_-6_)(*k*_+5_)*K*_9_(*k*_+11_)(*k*_-13_)(*k*_+15_) 
         _+_ *K*_1_(*k*_+10_)(*k*_-2_)(*k*_+8_)(*k*_-6_)(*k*_+5_)*K*_9_(*k*_+11_)(*k*_-13_)(*k*_-14_) 
         _+_ *K*_1_(*k*_+10_)(*k*_-2_)(*k*_+8_)(*k*_-6_)(*k*_+5_)*K*_9_(*k*_+11_)(*k*_+12_)(*k*_+15_) 
         _+_ *K*_1_(*k*_+10_)(*k*_-2_)(*k*_+8_)(*k*_-6_)(*k*_+5_)*K*_9_(*k*_+11_)(*k*_+12_)(*k*_-14_) 
         _+_ *K*_1_(*k*_+10_)(*k*_-2_)(*k*_+8_)(*k*_-6_)(*k*_-4_)*K*_9_(*k*_+11_)(*k*_-13_)(*k*_+15_) 
         _+_ *K*_1_(*k*_+10_)(*k*_-2_)(*k*_+8_)(*k*_-6_)(*k*_-4_)*K*_9_(*k*_+11_)(*k*_-13_)(*k*_-14_) 
         _+_ *K*_1_(*k*_+10_)(*k*_-2_)(*k*_+8_)(*k*_-6_)(*k*_-4_)*K*_9_(*k*_+11_)(*k*_+12_)(*k*_+15_) 
         _+_ *K*_1_(*k*_+10_)(*k*_-2_)(*k*_+8_)(*k*_-6_)(*k*_-4_)*K*_9_(*k*_+11_)(*k*_+12_)(*k*_-14_) 
         _+_ *K*_1_(*k*_+10_)(*k*_+3_)(*k*_+8_)(*k*_+7_)(*k*_+5_)*K*_9_(*k*_+11_)(*k*_-13_)(*k*_+15_) 
         _+_ *K*_1_(*k*_+10_)(*k*_+3_)(*k*_+8_)(*k*_+7_)(*k*_+5_)*K*_9_(*k*_+11_)(*k*_-13_)(*k*_-14_) 
         _+_ *K*_1_(*k*_+10_)(*k*_+3_)(*k*_+8_)(*k*_+7_)(*k*_+5_)*K*_9_(*k*_+11_)(*k*_+12_)(*k*_+15_) 
         _+_ *K*_1_(*k*_+10_)(*k*_+3_)(*k*_+8_)(*k*_+7_)(*k*_+5_)*K*_9_(*k*_+11_)(*k*_+12_)(*k*_-14_) 
         _+_ *K*_1_(*k*_+10_)(*k*_+3_)(*k*_+8_)(*k*_+7_)(*k*_-4_)*K*_9_(*k*_+11_)(*k*_-13_)(*k*_+15_) 
         _+_ *K*_1_(*k*_+10_)(*k*_+3_)(*k*_+8_)(*k*_+7_)(*k*_-4_)*K*_9_(*k*_+11_)(*k*_-13_)(*k*_-14_) 
         _+_ *K*_1_(*k*_+10_)(*k*_+3_)(*k*_+8_)(*k*_+7_)(*k*_-4_)*K*_9_(*k*_+11_)(*k*_+12_)(*k*_+15_) 
         _+_ *K*_1_(*k*_+10_)(*k*_+3_)(*k*_+8_)(*k*_+7_)(*k*_-4_)*K*_9_(*k*_+11_)(*k*_+12_)(*k*_-14_) 
         _+_ *K*_1_(*k*_+10_)(*k*_+3_)(*k*_+8_)(*k*_-6_)(*k*_+5_)*K*_9_(*k*_+11_)(*k*_-13_)(*k*_+15_) 
         _+_ *K*_1_(*k*_+10_)(*k*_+3_)(*k*_+8_)(*k*_-6_)(*k*_+5_)*K*_9_(*k*_+11_)(*k*_-13_)(*k*_-14_) 
         _+_ *K*_1_(*k*_+10_)(*k*_+3_)(*k*_+8_)(*k*_-6_)(*k*_+5_)*K*_9_(*k*_+11_)(*k*_+12_)(*k*_+15_) 
         _+_ *K*_1_(*k*_+10_)(*k*_+3_)(*k*_+8_)(*k*_-6_)(*k*_+5_)*K*_9_(*k*_+11_)(*k*_+12_)(*k*_-14_) 
         _+_ *K*_1_(*k*_+10_)(*k*_+3_)(*k*_+8_)(*k*_-6_)(*k*_-4_)*K*_9_(*k*_+11_)(*k*_-13_)(*k*_+15_) 
         _+_ *K*_1_(*k*_+10_)(*k*_+3_)(*k*_+8_)(*k*_-6_)(*k*_-4_)*K*_9_(*k*_+11_)(*k*_-13_)(*k*_-14_) 
         _+_ *K*_1_(*k*_+10_)(*k*_+3_)(*k*_+8_)(*k*_-6_)(*k*_-4_)*K*_9_(*k*_+11_)(*k*_+12_)(*k*_+15_) 
         _+_ *K*_1_(*k*_+10_)(*k*_+3_)(*k*_+8_)(*k*_-6_)(*k*_-4_)*K*_9_(*k*_+11_)(*k*_+12_)(*k*_-14_)}
β_9_ = {*K*_1_(*k*_-2_)(*k*_+8_)(*k*_+4_)(*k*_+7_)(*k*_+5_)*K*_9_(*k*_-10_)(*k*_-13_)(*k*_+15_) 
         _+_ *K*_1_(*k*_-2_)(*k*_+8_)(*k*_+4_)(*k*_+7_)(*k*_+5_)*K*_9_(*k*_-10_)(*k*_-13_)(*k*_-14_) 
         _+_ *K*_1_(*k*_-2_)(*k*_+8_)(*k*_+4_)(*k*_+7_)(*k*_+5_)*K*_9_(*k*_-10_)(*k*_+12_)(*k*_+15_) 
         _+_ *K*_1_(*k*_-2_)(*k*_+8_)(*k*_+4_)(*k*_+7_)(*k*_+5_)*K*_9_(*k*_-10_)(*k*_+12_)(*k*_-14_) 
         _+_ *K*_1_(*k*_-2_)(*k*_+8_)(*k*_+4_)(*k*_+7_)(*k*_+5_)*K*_9_(*k*_+11_)(*k*_-13_)(*k*_+15_) 
         _+_ *K*_1_(*k*_-2_)(*k*_+8_)(*k*_+4_)(*k*_+7_)(*k*_+5_)*K*_9_(*k*_+11_)(*k*_-13_)(*k*_-14_) 
         _+_ *K*_1_(*k*_-2_)(*k*_+8_)(*k*_+4_)(*k*_+7_)(*k*_+5_)*K*_9_(*k*_+11_)(*k*_+12_)(*k*_+15_) 
         _+_ *K*_1_(*k*_-2_)(*k*_+8_)(*k*_+4_)(*k*_+7_)(*k*_+5_)*K*_9_(*k*_+11_)(*k*_+12_)(*k*_-14_) 
         _+_ *K*_1_(*k*_-2_)(*k*_+8_)(*k*_+4_)(*k*_-6_)(*k*_+5_)*K*_9_(*k*_-10_)(*k*_-13_)(*k*_+15_) 
         _+_ *K*_1_(*k*_-2_)(*k*_+8_)(*k*_+4_)(*k*_-6_)(*k*_+5_)*K*_9_(*k*_-10_)(*k*_-13_)(*k*_-14_) 
         _+_ *K*_1_(*k*_-2_)(*k*_+8_)(*k*_+4_)(*k*_-6_)(*k*_+5_)*K*_9_(*k*_-10_)(*k*_+12_)(*k*_+15_) 
         _+_ *K*_1_(*k*_-2_)(*k*_+8_)(*k*_+4_)(*k*_-6_)(*k*_+5_)*K*_9_(*k*_-10_)(*k*_+12_)(*k*_-14_) 
         _+_ *K*_1_(*k*_-2_)(*k*_+8_)(*k*_+4_)(*k*_-6_)(*k*_+5_)*K*_9_(*k*_+11_)(*k*_-13_)(*k*_+15_) 
         _+_ *K*_1_(*k*_-2_)(*k*_+8_)(*k*_+4_)(*k*_-6_)(*k*_+5_)*K*_9_(*k*_+11_)(*k*_-13_)(*k*_-14_) 
         _+_ *K*_1_(*k*_-2_)(*k*_+8_)(*k*_+4_)(*k*_-6_)(*k*_+5_)*K*_9_(*k*_+11_)(*k*_+12_)(*k*_+15_) 
         _+_ *K*_1_(*k*_-2_)(*k*_+8_)(*k*_+4_)(*k*_-6_)(*k*_+5_)*K*_9_(*k*_+11_)(*k*_+12_)(*k*_-14_)}
β_10_ = {*K*_1_(*k*_-2_)(*k*_+8_)(*k*_+6_)(*k*_+7_)(*k*_+5_)*K*_9_(*k*_-10_)(*k*_-13_)(*k*_+15_) 
         _+_ *K*_1_(*k*_-2_)(*k*_+8_)(*k*_+6_)(*k*_+7_)(*k*_+5_)*K*_9_(*k*_-10_)(*k*_-13_)(*k*_-14_) 
         _+_ *K*_1_(*k*_-2_)(*k*_+8_)(*k*_+6_)(*k*_+7_)(*k*_+5_)*K*_9_(*k*_-10_)(*k*_+12_)(*k*_+15_) 
         _+_ *K*_1_(*k*_-2_)(*k*_+8_)(*k*_+6_)(*k*_+7_)(*k*_+5_)*K*_9_(*k*_-10_)(*k*_+12_)(*k*_-14_) 
         _+_ *K*_1_(*k*_-2_)(*k*_+8_)(*k*_+6_)(*k*_+7_)(*k*_+5_)*K*_9_(*k*_+11_)(*k*_-13_)(*k*_+15_) 
         _+_ *K*_1_(*k*_-2_)(*k*_+8_)(*k*_+6_)(*k*_+7_)(*k*_+5_)*K*_9_(*k*_+11_)(*k*_-13_)(*k*_-14_) 
         _+_ *K*_1_(*k*_-2_)(*k*_+8_)(*k*_+6_)(*k*_+7_)(*k*_+5_)*K*_9_(*k*_+11_)(*k*_+12_)(*k*_+15_) 
         _+_ *K*_1_(*k*_-2_)(*k*_+8_)(*k*_+6_)(*k*_+7_)(*k*_+5_)*K*_9_(*k*_+11_)(*k*_+12_)(*k*_-14_) 
         _+_ *K*_1_(*k*_-2_)(*k*_+8_)(*k*_+6_)(*k*_+7_)(*k*_-4_)*K*_9_(*k*_-10_)(*k*_-13_)(*k*_+15_) 
         _+_ *K*_1_(*k*_-2_)(*k*_+8_)(*k*_+6_)(*k*_+7_)(*k*_-4_)*K*_9_(*k*_-10_)(*k*_-13_)(*k*_-14_) 
         _+_ *K*_1_(*k*_-2_)(*k*_+8_)(*k*_+6_)(*k*_+7_)(*k*_-4_)*K*_9_(*k*_-10_)(*k*_+12_)(*k*_+15_) 
         _+_ *K*_1_(*k*_-2_)(*k*_+8_)(*k*_+6_)(*k*_+7_)(*k*_-4_)*K*_9_(*k*_-10_)(*k*_+12_)(*k*_-14_) 
         _+_ *K*_1_(*k*_-2_)(*k*_+8_)(*k*_+6_)(*k*_+7_)(*k*_-4_)*K*_9_(*k*_+11_)(*k*_-13_)(*k*_+15_) 
         _+_ *K*_1_(*k*_-2_)(*k*_+8_)(*k*_+6_)(*k*_+7_)(*k*_-4_)*K*_9_(*k*_+11_)(*k*_-13_)(*k*_-14_) 
         _+_ *K*_1_(*k*_-2_)(*k*_+8_)(*k*_+6_)(*k*_+7_)(*k*_-4_)*K*_9_(*k*_+11_)(*k*_+12_)(*k*_+15_) 
         _+_ *K*_1_(*k*_-2_)(*k*_+8_)(*k*_+6_)(*k*_+7_)(*k*_-4_)*K*_9_(*k*_+11_)(*k*_+12_)(*k*_-14_) 
         _+_ *K*_1_(*k*_+3_)(*k*_+8_)(*k*_+6_)(*k*_+7_)(*k*_+5_)*K*_9_(*k*_-10_)(*k*_-13_)(*k*_+15_) 
         _+_ *K*_1_(*k*_+3_)(*k*_+8_)(*k*_+6_)(*k*_+7_)(*k*_+5_)*K*_9_(*k*_-10_)(*k*_-13_)(*k*_-14_) 
         _+_ *K*_1_(*k*_+3_)(*k*_+8_)(*k*_+6_)(*k*_+7_)(*k*_+5_)*K*_9_(*k*_-10_)(*k*_+12_)(*k*_+15_) 
         _+_ *K*_1_(*k*_+3_)(*k*_+8_)(*k*_+6_)(*k*_+7_)(*k*_+5_)*K*_9_(*k*_-10_)(*k*_+12_)(*k*_-14_) 
         _+_ *K*_1_(*k*_+3_)(*k*_+8_)(*k*_+6_)(*k*_+7_)(*k*_+5_)*K*_9_(*k*_+11_)(*k*_-13_)(*k*_+15_) 
         _+_ *K*_1_(*k*_+3_)(*k*_+8_)(*k*_+6_)(*k*_+7_)(*k*_+5_)*K*_9_(*k*_+11_)(*k*_-13_)(*k*_-14_) 
         _+_ *K*_1_(*k*_+3_)(*k*_+8_)(*k*_+6_)(*k*_+7_)(*k*_+5_)*K*_9_(*k*_+11_)(*k*_+12_)(*k*_+15_) 
         _+_ *K*_1_(*k*_+3_)(*k*_+8_)(*k*_+6_)(*k*_+7_)(*k*_+5_)*K*_9_(*k*_+11_)(*k*_+12_)(*k*_-14_) 
         _+_ *K*_1_(*k*_+3_)(*k*_+8_)(*k*_+6_)(*k*_+7_)(*k*_-4_)*K*_9_(*k*_-10_)(*k*_-13_)(*k*_+15_) 
         _+_ *K*_1_(*k*_+3_)(*k*_+8_)(*k*_+6_)(*k*_+7_)(*k*_-4_)*K*_9_(*k*_-10_)(*k*_-13_)(*k*_-14_) 
         _+_ *K*_1_(*k*_+3_)(*k*_+8_)(*k*_+6_)(*k*_+7_)(*k*_-4_)*K*_9_(*k*_-10_)(*k*_+12_)(*k*_+15_) 
         _+_ *K*_1_(*k*_+3_)(*k*_+8_)(*k*_+6_)(*k*_+7_)(*k*_-4_)*K*_9_(*k*_-10_)(*k*_+12_)(*k*_-14_) 
         _+_ *K*_1_(*k*_+3_)(*k*_+8_)(*k*_+6_)(*k*_+7_)(*k*_-4_)*K*_9_(*k*_+11_)(*k*_-13_)(*k*_+15_) 
         _+_ *K*_1_(*k*_+3_)(*k*_+8_)(*k*_+6_)(*k*_+7_)(*k*_-4_)*K*_9_(*k*_+11_)(*k*_-13_)(*k*_-14_) 
         _+_ *K*_1_(*k*_+3_)(*k*_+8_)(*k*_+6_)(*k*_+7_)(*k*_-4_)*K*_9_(*k*_+11_)(*k*_+12_)(*k*_+15_) 
         _+_ *K*_1_(*k*_+3_)(*k*_+8_)(*k*_+6_)(*k*_+7_)(*k*_-4_)*K*_9_(*k*_+11_)(*k*_+12_)(*k*_-14_) 
         _+_ *K*_1_(*k*_+2_)(*k*_+3_)(*k*_+8_)(*k*_+7_)(*k*_+5_)*K*_9_(*k*_-10_)(*k*_-13_)(*k*_+15_) 
         _+_ *K*_1_(*k*_+2_)(*k*_+3_)(*k*_+8_)(*k*_+7_)(*k*_+5_)*K*_9_(*k*_-10_)(*k*_-13_)(*k*_-14_) 
         _+_ *K*_1_(*k*_+2_)(*k*_+3_)(*k*_+8_)(*k*_+7_)(*k*_+5_)*K*_9_(*k*_-10_)(*k*_+12_)(*k*_+15_) 
         _+_ *K*_1_(*k*_+2_)(*k*_+3_)(*k*_+8_)(*k*_+7_)(*k*_+5_)*K*_9_(*k*_-10_)(*k*_+12_)(*k*_-14_) 
         _+_ *K*_1_(*k*_+2_)(*k*_+3_)(*k*_+8_)(*k*_+7_)(*k*_+5_)*K*_9_(*k*_+11_)(*k*_-13_)(*k*_+15_) 
         _+_ *K*_1_(*k*_+2_)(*k*_+3_)(*k*_+8_)(*k*_+7_)(*k*_+5_)*K*_9_(*k*_+11_)(*k*_-13_)(*k*_-14_) 
         _+_ *K*_1_(*k*_+2_)(*k*_+3_)(*k*_+8_)(*k*_+7_)(*k*_+5_)*K*_9_(*k*_+11_)(*k*_+12_)(*k*_+15_) 
         _+_ *K*_1_(*k*_+2_)(*k*_+3_)(*k*_+8_)(*k*_+7_)(*k*_+5_)*K*_9_(*k*_+11_)(*k*_+12_)(*k*_-14_) 
         _+_ *K*_1_(*k*_+2_)(*k*_+3_)(*k*_+8_)(*k*_+7_)(*k*_-4_)*K*_9_(*k*_-10_)(*k*_-13_)(*k*_+15_) 
         _+_ *K*_1_(*k*_+2_)(*k*_+3_)(*k*_+8_)(*k*_+7_)(*k*_-4_)*K*_9_(*k*_-10_)(*k*_-13_)(*k*_-14_) 
         _+_ *K*_1_(*k*_+2_)(*k*_+3_)(*k*_+8_)(*k*_+7_)(*k*_-4_)*K*_9_(*k*_-10_)(*k*_+12_)(*k*_+15_) 
         _+_ *K*_1_(*k*_+2_)(*k*_+3_)(*k*_+8_)(*k*_+7_)(*k*_-4_)*K*_9_(*k*_-10_)(*k*_+12_)(*k*_-14_) 
         _+_ *K*_1_(*k*_+2_)(*k*_+3_)(*k*_+8_)(*k*_+7_)(*k*_-4_)*K*_9_(*k*_+11_)(*k*_-13_)(*k*_+15_) 
         _+_ *K*_1_(*k*_+2_)(*k*_+3_)(*k*_+8_)(*k*_+7_)(*k*_-4_)*K*_9_(*k*_+11_)(*k*_-13_)(*k*_-14_) 
         _+_ *K*_1_(*k*_+2_)(*k*_+3_)(*k*_+8_)(*k*_+7_)(*k*_-4_)*K*_9_(*k*_+11_)(*k*_+12_)(*k*_+15_) 
         _+_ *K*_1_(*k*_+2_)(*k*_+3_)(*k*_+8_)(*k*_+7_)(*k*_-4_)*K*_9_(*k*_+11_)(*k*_+12_)(*k*_-14_) 
         _+_ *K*_1_(*k*_+2_)(*k*_+3_)(*k*_+8_)(*k*_-6_)(*k*_+5_)*K*_9_(*k*_-10_)(*k*_-13_)(*k*_+15_) 
         _+_ *K*_1_(*k*_+2_)(*k*_+3_)(*k*_+8_)(*k*_-6_)(*k*_+5_)*K*_9_(*k*_-10_)(*k*_-13_)(*k*_-14_) 
         _+_ *K*_1_(*k*_+2_)(*k*_+3_)(*k*_+8_)(*k*_-6_)(*k*_+5_)*K*_9_(*k*_-10_)(*k*_+12_)(*k*_+15_) 
         _+_ *K*_1_(*k*_+2_)(*k*_+3_)(*k*_+8_)(*k*_-6_)(*k*_+5_)*K*_9_(*k*_-10_)(*k*_+12_)(*k*_-14_) 
         _+_ *K*_1_(*k*_+2_)(*k*_+3_)(*k*_+8_)(*k*_-6_)(*k*_+5_)*K*_9_(*k*_+11_)(*k*_-13_)(*k*_+15_) 
         _+_ *K*_1_(*k*_+2_)(*k*_+3_)(*k*_+8_)(*k*_-6_)(*k*_+5_)*K*_9_(*k*_+11_)(*k*_-13_)(*k*_-14_) 
         _+_ *K*_1_(*k*_+2_)(*k*_+3_)(*k*_+8_)(*k*_-6_)(*k*_+5_)*K*_9_(*k*_+11_)(*k*_+12_)(*k*_+15_) 
         _+_ *K*_1_(*k*_+2_)(*k*_+3_)(*k*_+8_)(*k*_-6_)(*k*_+5_)*K*_9_(*k*_+11_)(*k*_+12_)(*k*_-14_) 
         _+_ *K*_1_(*k*_+2_)(*k*_+3_)(*k*_+8_)(*k*_-6_)(*k*_-4_)*K*_9_(*k*_-10_)(*k*_-13_)(*k*_+15_) 
         _+_ *K*_1_(*k*_+2_)(*k*_+3_)(*k*_+8_)(*k*_-6_)(*k*_-4_)*K*_9_(*k*_-10_)(*k*_-13_)(*k*_-14_) 
         _+_ *K*_1_(*k*_+2_)(*k*_+3_)(*k*_+8_)(*k*_-6_)(*k*_-4_)*K*_9_(*k*_-10_)(*k*_+12_)(*k*_+15_) 
         _+_ *K*_1_(*k*_+2_)(*k*_+3_)(*k*_+8_)(*k*_-6_)(*k*_-4_)*K*_9_(*k*_-10_)(*k*_+12_)(*k*_-14_) 
         _+_ *K*_1_(*k*_+2_)(*k*_+3_)(*k*_+8_)(*k*_-6_)(*k*_-4_)*K*_9_(*k*_+11_)(*k*_-13_)(*k*_+15_) 
         _+_ *K*_1_(*k*_+2_)(*k*_+3_)(*k*_+8_)(*k*_-6_)(*k*_-4_)*K*_9_(*k*_+11_)(*k*_-13_)(*k*_-14_) 
         _+_ *K*_1_(*k*_+2_)(*k*_+3_)(*k*_+8_)(*k*_-6_)(*k*_-4_)*K*_9_(*k*_+11_)(*k*_+12_)(*k*_+15_) 
         _+_ *K*_1_(*k*_+2_)(*k*_+3_)(*k*_+8_)(*k*_-6_)(*k*_-4_)*K*_9_(*k*_+11_)(*k*_+12_)(*k*_-14_)}
β_11_ = {*K*_1_(*k*_+10_)(*k*_-2_)(*k*_+13_)(*k*_+7_)(*k*_+5_)*K*_9_(*k*_+11_)(*k*_+12_)(*k*_+15_) 
         _+_ *K*_1_(*k*_+10_)(*k*_-2_)(*k*_+13_)(*k*_+7_)(*k*_+5_)*K*_9_(*k*_+11_)(*k*_+12_)(*k*_-14_) 
         _+_ *K*_1_(*k*_+10_)(*k*_-2_)(*k*_+13_)(*k*_+7_)(*k*_-4_)*K*_9_(*k*_+11_)(*k*_+12_)(*k*_+15_) 
         _+_ *K*_1_(*k*_+10_)(*k*_-2_)(*k*_+13_)(*k*_+7_)(*k*_-4_)*K*_9_(*k*_+11_)(*k*_+12_)(*k*_-14_) 
         _+_ *K*_1_(*k*_+10_)(*k*_-2_)(*k*_+13_)(*k*_-6_)(*k*_+5_)*K*_9_(*k*_+11_)(*k*_+12_)(*k*_+15_) 
         _+_ *K*_1_(*k*_+10_)(*k*_-2_)(*k*_+13_)(*k*_-6_)(*k*_+5_)*K*_9_(*k*_+11_)(*k*_+12_)(*k*_-14_) 
         _+_ *K*_1_(*k*_+10_)(*k*_-2_)(*k*_+13_)(*k*_-6_)(*k*_-4_)*K*_9_(*k*_+11_)(*k*_+12_)(*k*_+15_) 
         _+_ *K*_1_(*k*_+10_)(*k*_-2_)(*k*_+13_)(*k*_-6_)(*k*_-4_)*K*_9_(*k*_+11_)(*k*_+12_)(*k*_-14_) 
         _+_ *K*_1_(*k*_+10_)(*k*_+3_)(*k*_+13_)(*k*_+7_)(*k*_+5_)*K*_9_(*k*_+11_)(*k*_+12_)(*k*_+15_) 
         _+_ *K*_1_(*k*_+10_)(*k*_+3_)(*k*_+13_)(*k*_+7_)(*k*_+5_)*K*_9_(*k*_+11_)(*k*_+12_)(*k*_-14_) 
         _+_ *K*_1_(*k*_+10_)(*k*_+3_)(*k*_+13_)(*k*_+7_)(*k*_-4_)*K*_9_(*k*_+11_)(*k*_+12_)(*k*_+15_) 
         _+_ *K*_1_(*k*_+10_)(*k*_+3_)(*k*_+13_)(*k*_+7_)(*k*_-4_)*K*_9_(*k*_+11_)(*k*_+12_)(*k*_-14_) 
         _+_ *K*_1_(*k*_+10_)(*k*_+3_)(*k*_+13_)(*k*_-6_)(*k*_+5_)*K*_9_(*k*_+11_)(*k*_+12_)(*k*_+15_) 
         _+_ *K*_1_(*k*_+10_)(*k*_+3_)(*k*_+13_)(*k*_-6_)(*k*_+5_)*K*_9_(*k*_+11_)(*k*_+12_)(*k*_-14_) 
         _+_ *K*_1_(*k*_+10_)(*k*_+3_)(*k*_+13_)(*k*_-6_)(*k*_-4_)*K*_9_(*k*_+11_)(*k*_+12_)(*k*_+15_) 
         _+_ *K*_1_(*k*_+10_)(*k*_+3_)(*k*_+13_)(*k*_-6_)(*k*_-4_)*K*_9_(*k*_+11_)(*k*_+12_)(*k*_-14_)}
β_12_ = {*K*_1_(*k*_+2_)(*k*_+3_)(*k*_+13_)(*k*_+7_)(*k*_+5_)*K*_9_(*k*_-10_)(*k*_+12_)(*k*_+15_) 
         _+_ *K*_1_(*k*_+2_)(*k*_+3_)(*k*_+13_)(*k*_+7_)(*k*_+5_)*K*_9_(*k*_-10_)(*k*_+12_)(*k*_-14_) 
         _+_ *K*_1_(*k*_+2_)(*k*_+3_)(*k*_+13_)(*k*_+7_)(*k*_+5_)*K*_9_(*k*_+11_)(*k*_+12_)(*k*_+15_) 
         _+_ *K*_1_(*k*_+2_)(*k*_+3_)(*k*_+13_)(*k*_+7_)(*k*_+5_)*K*_9_(*k*_+11_)(*k*_+12_)(*k*_-14_) 
         _+_ *K*_1_(*k*_+2_)(*k*_+3_)(*k*_+13_)(*k*_+7_)(*k*_-4_)*K*_9_(*k*_-10_)(*k*_+12_)(*k*_+15_) 
         _+_ *K*_1_(*k*_+2_)(*k*_+3_)(*k*_+13_)(*k*_+7_)(*k*_-4_)*K*_9_(*k*_-10_)(*k*_+12_)(*k*_-14_) 
         _+_ *K*_1_(*k*_+2_)(*k*_+3_)(*k*_+13_)(*k*_+7_)(*k*_-4_)*K*_9_(*k*_+11_)(*k*_+12_)(*k*_+15_) 
         _+_ *K*_1_(*k*_+2_)(*k*_+3_)(*k*_+13_)(*k*_+7_)(*k*_-4_)*K*_9_(*k*_+11_)(*k*_+12_)(*k*_-14_) 
         _+_ *K*_1_(*k*_+2_)(*k*_+3_)(*k*_+13_)(*k*_-6_)(*k*_+5_)*K*_9_(*k*_-10_)(*k*_+12_)(*k*_+15_) 
         _+_ *K*_1_(*k*_+2_)(*k*_+3_)(*k*_+13_)(*k*_-6_)(*k*_+5_)*K*_9_(*k*_-10_)(*k*_+12_)(*k*_-14_) 
         _+_ *K*_1_(*k*_+2_)(*k*_+3_)(*k*_+13_)(*k*_-6_)(*k*_+5_)*K*_9_(*k*_+11_)(*k*_+12_)(*k*_+15_) 
         _+_ *K*_1_(*k*_+2_)(*k*_+3_)(*k*_+13_)(*k*_-6_)(*k*_+5_)*K*_9_(*k*_+11_)(*k*_+12_)(*k*_-14_) 
         _+_ *K*_1_(*k*_+2_)(*k*_+3_)(*k*_+13_)(*k*_-6_)(*k*_-4_)*K*_9_(*k*_-10_)(*k*_+12_)(*k*_+15_) 
         _+_ *K*_1_(*k*_+2_)(*k*_+3_)(*k*_+13_)(*k*_-6_)(*k*_-4_)*K*_9_(*k*_-10_)(*k*_+12_)(*k*_-14_) 
         _+_ *K*_1_(*k*_+2_)(*k*_+3_)(*k*_+13_)(*k*_-6_)(*k*_-4_)*K*_9_(*k*_+11_)(*k*_+12_)(*k*_+15_) 
         _+_ *K*_1_(*k*_+2_)(*k*_+3_)(*k*_+13_)(*k*_-6_)(*k*_-4_)*K*_9_(*k*_+11_)(*k*_+12_)(*k*_-14_)}
β_13_ = {*K*_1_(*k*_-2_)(*k*_+8_)(*k*_+13_)(*k*_+7_)(*k*_+5_)*K*_9_(*k*_-10_)(*k*_+12_)(*k*_+15_) 
         _+_ *K*_1_(*k*_-2_)(*k*_+8_)(*k*_+13_)(*k*_+7_)(*k*_+5_)*K*_9_(*k*_-10_)(*k*_+12_)(*k*_-14_) 
         _+_ *K*_1_(*k*_-2_)(*k*_+8_)(*k*_+13_)(*k*_+7_)(*k*_-4_)*K*_9_(*k*_-10_)(*k*_+12_)(*k*_+15_) 
         _+_ *K*_1_(*k*_-2_)(*k*_+8_)(*k*_+13_)(*k*_+7_)(*k*_-4_)*K*_9_(*k*_-10_)(*k*_+12_)(*k*_-14_) 
         _+_ *K*_1_(*k*_-2_)(*k*_+8_)(*k*_+13_)(*k*_-6_)(*k*_+5_)*K*_9_(*k*_-10_)(*k*_+12_)(*k*_+15_) 
         _+_ *K*_1_(*k*_-2_)(*k*_+8_)(*k*_+13_)(*k*_-6_)(*k*_+5_)*K*_9_(*k*_-10_)(*k*_+12_)(*k*_-14_) 
         _+_ *K*_1_(*k*_-2_)(*k*_+8_)(*k*_+13_)(*k*_-6_)(*k*_-4_)*K*_9_(*k*_-10_)(*k*_+12_)(*k*_+15_) 
         _+_ *K*_1_(*k*_-2_)(*k*_+8_)(*k*_+13_)(*k*_-6_)(*k*_-4_)*K*_9_(*k*_-10_)(*k*_+12_)(*k*_-14_) 
         _+_ *K*_1_(*k*_+3_)(*k*_+8_)(*k*_+13_)(*k*_+7_)(*k*_+5_)*K*_9_(*k*_-10_)(*k*_+12_)(*k*_+15_) 
         _+_ *K*_1_(*k*_+3_)(*k*_+8_)(*k*_+13_)(*k*_+7_)(*k*_+5_)*K*_9_(*k*_-10_)(*k*_+12_)(*k*_-14_) 
         _+_ *K*_1_(*k*_+3_)(*k*_+8_)(*k*_+13_)(*k*_+7_)(*k*_-4_)*K*_9_(*k*_-10_)(*k*_+12_)(*k*_+15_) 
         _+_ *K*_1_(*k*_+3_)(*k*_+8_)(*k*_+13_)(*k*_+7_)(*k*_-4_)*K*_9_(*k*_-10_)(*k*_+12_)(*k*_-14_) 
         _+_ *K*_1_(*k*_+3_)(*k*_+8_)(*k*_+13_)(*k*_-6_)(*k*_+5_)*K*_9_(*k*_-10_)(*k*_+12_)(*k*_+15_) 
         _+_ *K*_1_(*k*_+3_)(*k*_+8_)(*k*_+13_)(*k*_-6_)(*k*_+5_)*K*_9_(*k*_-10_)(*k*_+12_)(*k*_-14_) 
         _+_ *K*_1_(*k*_+3_)(*k*_+8_)(*k*_+13_)(*k*_-6_)(*k*_-4_)*K*_9_(*k*_-10_)(*k*_+12_)(*k*_+15_) 
         _+_ *K*_1_(*k*_+3_)(*k*_+8_)(*k*_+13_)(*k*_-6_)(*k*_-4_)*K*_9_(*k*_-10_)(*k*_+12_)(*k*_-14_)}
β_14_ = {*K*_1_(*k*_+10_)(*k*_-2_)(*k*_+8_)(*k*_+14_)(*k*_+7_)(*k*_+5_)*K*_9_(*k*_-13_)(*k*_+15_) 
         _+_ *K*_1_(*k*_+10_)(*k*_-2_)(*k*_+8_)(*k*_+14_)(*k*_+7_)(*k*_+5_)*K*_9_(*k*_+12_)(*k*_+15_) 
         _+_ *K*_1_(*k*_+10_)(*k*_-2_)(*k*_+8_)(*k*_+14_)(*k*_+7_)(*k*_-4_)*K*_9_(*k*_-13_)(*k*_+15_) 
         _+_ *K*_1_(*k*_+10_)(*k*_-2_)(*k*_+8_)(*k*_+14_)(*k*_+7_)(*k*_-4_)*K*_9_(*k*_+12_)(*k*_+15_) 
         _+_ *K*_1_(*k*_+10_)(*k*_-2_)(*k*_+8_)(*k*_+14_)(*k*_-6_)(*k*_+5_)*K*_9_(*k*_-13_)(*k*_+15_) 
         _+_ *K*_1_(*k*_+10_)(*k*_-2_)(*k*_+8_)(*k*_+14_)(*k*_-6_)(*k*_+5_)*K*_9_(*k*_+12_)(*k*_+15_) 
         _+_ *K*_1_(*k*_+10_)(*k*_-2_)(*k*_+8_)(*k*_+14_)(*k*_-6_)(*k*_-4_)*K*_9_(*k*_-13_)(*k*_+15_) 
         _+_ *K*_1_(*k*_+10_)(*k*_-2_)(*k*_+8_)(*k*_+14_)(*k*_-6_)(*k*_-4_)*K*_9_(*k*_+12_)(*k*_+15_) 
         _+_ *K*_1_(*k*_+10_)(*k*_+3_)(*k*_+8_)(*k*_+14_)(*k*_+7_)(*k*_+5_)*K*_9_(*k*_-13_)(*k*_+15_) 
         _+_ *K*_1_(*k*_+10_)(*k*_+3_)(*k*_+8_)(*k*_+14_)(*k*_+7_)(*k*_+5_)*K*_9_(*k*_+12_)(*k*_+15_) 
         _+_ *K*_1_(*k*_+10_)(*k*_+3_)(*k*_+8_)(*k*_+14_)(*k*_+7_)(*k*_-4_)*K*_9_(*k*_-13_)(*k*_+15_) 
         _+_ *K*_1_(*k*_+10_)(*k*_+3_)(*k*_+8_)(*k*_+14_)(*k*_+7_)(*k*_-4_)*K*_9_(*k*_+12_)(*k*_+15_) 
         _+_ *K*_1_(*k*_+10_)(*k*_+3_)(*k*_+8_)(*k*_+14_)(*k*_-6_)(*k*_+5_)*K*_9_(*k*_-13_)(*k*_+15_) 
         _+_ *K*_1_(*k*_+10_)(*k*_+3_)(*k*_+8_)(*k*_+14_)(*k*_-6_)(*k*_+5_)*K*_9_(*k*_+12_)(*k*_+15_) 
         _+_ *K*_1_(*k*_+10_)(*k*_+3_)(*k*_+8_)(*k*_+14_)(*k*_-6_)(*k*_-4_)*K*_9_(*k*_-13_)(*k*_+15_) 
         _+_ *K*_1_(*k*_+10_)(*k*_+3_)(*k*_+8_)(*k*_+14_)(*k*_-6_)(*k*_-4_)*K*_9_(*k*_+12_)(*k*_+15_) 
         _+_ *K*_1_(*k*_+10_)(*k*_-2_)(*k*_+8_)(*k*_+14_)(*k*_+7_)(*k*_+5_)*K*_9_(*k*_+11_)(*k*_-13_) 
         _+_ *K*_1_(*k*_+10_)(*k*_-2_)(*k*_+8_)(*k*_+14_)(*k*_+7_)(*k*_+5_)*K*_9_(*k*_+11_)(*k*_+12_) 
         _+_ *K*_1_(*k*_+10_)(*k*_-2_)(*k*_+8_)(*k*_+14_)(*k*_+7_)(*k*_-4_)*K*_9_(*k*_+11_)(*k*_-13_) 
         _+_ *K*_1_(*k*_+10_)(*k*_-2_)(*k*_+8_)(*k*_+14_)(*k*_+7_)(*k*_-4_)*K*_9_(*k*_+11_)(*k*_+12_) 
         _+_ *K*_1_(*k*_+10_)(*k*_-2_)(*k*_+8_)(*k*_+14_)(*k*_-6_)(*k*_+5_)*K*_9_(*k*_+11_)(*k*_-13_) 
         _+_ *K*_1_(*k*_+10_)(*k*_-2_)(*k*_+8_)(*k*_+14_)(*k*_-6_)(*k*_+5_)*K*_9_(*k*_+11_)(*k*_+12_) 
         _+_ *K*_1_(*k*_+10_)(*k*_-2_)(*k*_+8_)(*k*_+14_)(*k*_-6_)(*k*_-4_)*K*_9_(*k*_+11_)(*k*_-13_) 
         _+_ *K*_1_(*k*_+10_)(*k*_-2_)(*k*_+8_)(*k*_+14_)(*k*_-6_)(*k*_-4_)*K*_9_(*k*_+11_)(*k*_+12_) 
         _+_ *K*_1_(*k*_+10_)(*k*_+3_)(*k*_+8_)(*k*_+14_)(*k*_+7_)(*k*_+5_)*K*_9_(*k*_+11_)(*k*_-13_) 
         _+_ *K*_1_(*k*_+10_)(*k*_+3_)(*k*_+8_)(*k*_+14_)(*k*_+7_)(*k*_+5_)*K*_9_(*k*_+11_)(*k*_+12_) 
         _+_ *K*_1_(*k*_+10_)(*k*_+3_)(*k*_+8_)(*k*_+14_)(*k*_+7_)(*k*_-4_)*K*_9_(*k*_+11_)(*k*_-13_) 
         _+_ *K*_1_(*k*_+10_)(*k*_+3_)(*k*_+8_)(*k*_+14_)(*k*_+7_)(*k*_-4_)*K*_9_(*k*_+11_)(*k*_+12_) 
         _+_ *K*_1_(*k*_+10_)(*k*_+3_)(*k*_+8_)(*k*_+14_)(*k*_-6_)(*k*_+5_)*K*_9_(*k*_+11_)(*k*_-13_) 
         _+_ *K*_1_(*k*_+10_)(*k*_+3_)(*k*_+8_)(*k*_+14_)(*k*_-6_)(*k*_+5_)*K*_9_(*k*_+11_)(*k*_+12_) 
         _+_ *K*_1_(*k*_+10_)(*k*_+3_)(*k*_+8_)(*k*_+14_)(*k*_-6_)(*k*_-4_)*K*_9_(*k*_+11_)(*k*_-13_) 
         _+_ *K*_1_(*k*_+10_)(*k*_+3_)(*k*_+8_)(*k*_+14_)(*k*_-6_)(*k*_-4_)*K*_9_(*k*_+11_)(*k*_+12_)}
β_15_ = {(*k*_-2_)(*k*_+8_)(*k*_+14_)(*k*_+7_)(*k*_+5_)*K*_9_(*k*_-10_)(*k*_-13_)(*k*_+15_) 
         _+_ (*k*_-2_)(*k*_+8_)(*k*_+14_)(*k*_+7_)(*k*_+5_)*K*_9_(*k*_-10_)(*k*_+12_)(*k*_+15_) 
         _+_ (*k*_-2_)(*k*_+8_)(*k*_+14_)(*k*_+7_)(*k*_+5_)*K*_9_(*k*_+11_)(*k*_-13_)(*k*_+15_) 
         _+_ (*k*_-2_)(*k*_+8_)(*k*_+14_)(*k*_+7_)(*k*_+5_)*K*_9_(*k*_+11_)(*k*_+12_)(*k*_+15_) 
         _+_ (*k*_-2_)(*k*_+8_)(*k*_+14_)(*k*_+7_)(*k*_-4_)*K*_9_(*k*_-10_)(*k*_-13_)(*k*_+15_) 
         _+_ (*k*_-2_)(*k*_+8_)(*k*_+14_)(*k*_+7_)(*k*_-4_)*K*_9_(*k*_-10_)(*k*_+12_)(*k*_+15_) 
         _+_ (*k*_-2_)(*k*_+8_)(*k*_+14_)(*k*_+7_)(*k*_-4_)*K*_9_(*k*_+11_)(*k*_-13_)(*k*_+15_) 
         _+_ (*k*_-2_)(*k*_+8_)(*k*_+14_)(*k*_+7_)(*k*_-4_)*K*_9_(*k*_+11_)(*k*_+12_)(*k*_+15_) 
         _+_ (*k*_-2_)(*k*_+8_)(*k*_+14_)(*k*_-6_)(*k*_+5_)*K*_9_(*k*_-10_)(*k*_-13_)(*k*_+15_) 
         _+_ (*k*_-2_)(*k*_+8_)(*k*_+14_)(*k*_-6_)(*k*_+5_)*K*_9_(*k*_-10_)(*k*_+12_)(*k*_+15_) 
         _+_ (*k*_-2_)(*k*_+8_)(*k*_+14_)(*k*_-6_)(*k*_+5_)*K*_9_(*k*_+11_)(*k*_-13_)(*k*_+15_) 
         _+_ (*k*_-2_)(*k*_+8_)(*k*_+14_)(*k*_-6_)(*k*_+5_)*K*_9_(*k*_+11_)(*k*_+12_)(*k*_+15_) 
         _+_ (*k*_-2_)(*k*_+8_)(*k*_+14_)(*k*_-6_)(*k*_-4_)*K*_9_(*k*_-10_)(*k*_-13_)(*k*_+15_) 
         _+_ (*k*_-2_)(*k*_+8_)(*k*_+14_)(*k*_-6_)(*k*_-4_)*K*_9_(*k*_-10_)(*k*_+12_)(*k*_+15_) 
         _+_ (*k*_-2_)(*k*_+8_)(*k*_+14_)(*k*_-6_)(*k*_-4_)*K*_9_(*k*_+11_)(*k*_-13_)(*k*_+15_) 
         _+_ (*k*_-2_)(*k*_+8_)(*k*_+14_)(*k*_-6_)(*k*_-4_)*K*_9_(*k*_+11_)(*k*_+12_)(*k*_+15_) 
         _+_ (*k*_+3_)(*k*_+8_)(*k*_+14_)(*k*_+7_)(*k*_+5_)*K*_9_(*k*_-10_)(*k*_-13_)(*k*_+15_) 
         _+_ (*k*_+3_)(*k*_+8_)(*k*_+14_)(*k*_+7_)(*k*_+5_)*K*_9_(*k*_-10_)(*k*_+12_)(*k*_+15_) 
         _+_ (*k*_+3_)(*k*_+8_)(*k*_+14_)(*k*_+7_)(*k*_+5_)*K*_9_(*k*_+11_)(*k*_-13_)(*k*_+15_) 
         _+_ (*k*_+3_)(*k*_+8_)(*k*_+14_)(*k*_+7_)(*k*_+5_)*K*_9_(*k*_+11_)(*k*_+12_)(*k*_+15_) 
         _+_ (*k*_+3_)(*k*_+8_)(*k*_+14_)(*k*_+7_)(*k*_-4_)*K*_9_(*k*_-10_)(*k*_-13_)(*k*_+15_) 
         _+_ (*k*_+3_)(*k*_+8_)(*k*_+14_)(*k*_+7_)(*k*_-4_)*K*_9_(*k*_-10_)(*k*_+12_)(*k*_+15_) 
         _+_ (*k*_+3_)(*k*_+8_)(*k*_+14_)(*k*_+7_)(*k*_-4_)*K*_9_(*k*_+11_)(*k*_-13_)(*k*_+15_) 
         _+_ (*k*_+3_)(*k*_+8_)(*k*_+14_)(*k*_+7_)(*k*_-4_)*K*_9_(*k*_+11_)(*k*_+12_)(*k*_+15_) 
         _+_ (*k*_+3_)(*k*_+8_)(*k*_+14_)(*k*_-6_)(*k*_+5_)*K*_9_(*k*_-10_)(*k*_-13_)(*k*_+15_) 
         _+_ (*k*_+3_)(*k*_+8_)(*k*_+14_)(*k*_-6_)(*k*_+5_)*K*_9_(*k*_-10_)(*k*_+12_)(*k*_+15_) 
         _+_ (*k*_+3_)(*k*_+8_)(*k*_+14_)(*k*_-6_)(*k*_+5_)*K*_9_(*k*_+11_)(*k*_-13_)(*k*_+15_) 
         _+_ (*k*_+3_)(*k*_+8_)(*k*_+14_)(*k*_-6_)(*k*_+5_)*K*_9_(*k*_+11_)(*k*_+12_)(*k*_+15_) 
         _+_ (*k*_+3_)(*k*_+8_)(*k*_+14_)(*k*_-6_)(*k*_-4_)*K*_9_(*k*_-10_)(*k*_-13_)(*k*_+15_) 
         _+_ (*k*_+3_)(*k*_+8_)(*k*_+14_)(*k*_-6_)(*k*_-4_)*K*_9_(*k*_-10_)(*k*_+12_)(*k*_+15_) 
         _+_ (*k*_+3_)(*k*_+8_)(*k*_+14_)(*k*_-6_)(*k*_-4_)*K*_9_(*k*_+11_)(*k*_-13_)(*k*_+15_) 
         _+_ (*k*_+3_)(*k*_+8_)(*k*_+14_)(*k*_-6_)(*k*_-4_)*K*_9_(*k*_+11_)(*k*_+12_)(*k*_+15_) 
         _+_ *K*_1_(*k*_+10_)(*k*_+8_)(*k*_+4_)(*k*_+7_)(*k*_+5_)*K*_9_(*k*_+11_)(*k*_-13_)(*k*_+15_) 
         _+_ *K*_1_(*k*_+10_)(*k*_+8_)(*k*_+4_)(*k*_+7_)(*k*_+5_)*K*_9_(*k*_+11_)(*k*_-13_)(*k*_-14_) 
         _+_ *K*_1_(*k*_+10_)(*k*_+8_)(*k*_+4_)(*k*_+7_)(*k*_+5_)*K*_9_(*k*_+11_)(*k*_+12_)(*k*_+15_) 
         _+_ *K*_1_(*k*_+10_)(*k*_+8_)(*k*_+4_)(*k*_+7_)(*k*_+5_)*K*_9_(*k*_+11_)(*k*_+12_)(*k*_-14_) 
         _+_ *K*_1_(*k*_+10_)(*k*_+8_)(*k*_+4_)(*k*_-6_)(*k*_+5_)*K*_9_(*k*_+11_)(*k*_-13_)(*k*_+15_) 
         _+_ *K*_1_(*k*_+10_)(*k*_+8_)(*k*_+4_)(*k*_-6_)(*k*_+5_)*K*_9_(*k*_+11_)(*k*_-13_)(*k*_-14_) 
         _+_ *K*_1_(*k*_+10_)(*k*_+8_)(*k*_+4_)(*k*_-6_)(*k*_+5_)*K*_9_(*k*_+11_)(*k*_+12_)(*k*_+15_) 
         _+_ *K*_1_(*k*_+10_)(*k*_+8_)(*k*_+4_)(*k*_-6_)(*k*_+5_)*K*_9_(*k*_+11_)(*k*_+12_)(*k*_-14_) 
         _+_ *K*_1_(*k*_+10_)(*k*_-2_)(*k*_+8_)(*k*_+4_)(*k*_+7_)*K*_9_(*k*_+11_)(*k*_-13_)(*k*_+15_) 
         _+_ *K*_1_(*k*_+10_)(*k*_-2_)(*k*_+8_)(*k*_+4_)(*k*_+7_)*K*_9_(*k*_+11_)(*k*_-13_)(*k*_-14_) 
         _+_ *K*_1_(*k*_+10_)(*k*_-2_)(*k*_+8_)(*k*_+4_)(*k*_+7_)*K*_9_(*k*_+11_)(*k*_+12_)(*k*_+15_) 
         _+_ *K*_1_(*k*_+10_)(*k*_-2_)(*k*_+8_)(*k*_+4_)(*k*_+7_)*K*_9_(*k*_+11_)(*k*_+12_)(*k*_-14_) 
         _+_ *K*_1_(*k*_+10_)(*k*_-2_)(*k*_+8_)(*k*_+4_)(*k*_-6_)*K*_9_(*k*_+11_)(*k*_-13_)(*k*_+15_) 
         _+_ *K*_1_(*k*_+10_)(*k*_-2_)(*k*_+8_)(*k*_+4_)(*k*_-6_)*K*_9_(*k*_+11_)(*k*_-13_)(*k*_-14_) 
         _+_ *K*_1_(*k*_+10_)(*k*_-2_)(*k*_+8_)(*k*_+4_)(*k*_-6_)*K*_9_(*k*_+11_)(*k*_+12_)(*k*_+15_) 
         _+_ *K*_1_(*k*_+10_)(*k*_-2_)(*k*_+8_)(*k*_+4_)(*k*_-6_)*K*_9_(*k*_+11_)(*k*_+12_)(*k*_-14_) 
         _+_ *K*_1_(*k*_+10_)(*k*_+3_)(*k*_+8_)(*k*_+4_)(*k*_+7_)*K*_9_(*k*_+11_)(*k*_-13_)(*k*_+15_) 
         _+_ *K*_1_(*k*_+10_)(*k*_+3_)(*k*_+8_)(*k*_+4_)(*k*_+7_)*K*_9_(*k*_+11_)(*k*_-13_)(*k*_-14_) 
         _+_ *K*_1_(*k*_+10_)(*k*_+3_)(*k*_+8_)(*k*_+4_)(*k*_+7_)*K*_9_(*k*_+11_)(*k*_+12_)(*k*_+15_) 
         _+_ *K*_1_(*k*_+10_)(*k*_+3_)(*k*_+8_)(*k*_+4_)(*k*_+7_)*K*_9_(*k*_+11_)(*k*_+12_)(*k*_-14_) 
         _+_ *K*_1_(*k*_+10_)(*k*_+3_)(*k*_+8_)(*k*_+4_)(*k*_-6_)*K*_9_(*k*_+11_)(*k*_-13_)(*k*_+15_) 
         _+_ *K*_1_(*k*_+10_)(*k*_+3_)(*k*_+8_)(*k*_+4_)(*k*_-6_)*K*_9_(*k*_+11_)(*k*_-13_)(*k*_-14_) 
         _+_ *K*_1_(*k*_+10_)(*k*_+3_)(*k*_+8_)(*k*_+4_)(*k*_-6_)*K*_9_(*k*_+11_)(*k*_+12_)(*k*_+15_) 
         _+_ *K*_1_(*k*_+10_)(*k*_+3_)(*k*_+8_)(*k*_+4_)(*k*_-6_)*K*_9_(*k*_+11_)(*k*_+12_)(*k*_-14_) 
         _+_ *K*_1_(*k*_+10_)(*k*_-2_)(*k*_+8_)(*k*_+4_)(*k*_+7_)(*k*_+5_)*K*_9_(*k*_-13_)(*k*_+15_) 
         _+_ *K*_1_(*k*_+10_)(*k*_-2_)(*k*_+8_)(*k*_+4_)(*k*_+7_)(*k*_+5_)*K*_9_(*k*_-13_)(*k*_-14_) 
         _+_ *K*_1_(*k*_+10_)(*k*_-2_)(*k*_+8_)(*k*_+4_)(*k*_+7_)(*k*_+5_)*K*_9_(*k*_+12_)(*k*_+15_) 
         _+_ *K*_1_(*k*_+10_)(*k*_-2_)(*k*_+8_)(*k*_+4_)(*k*_+7_)(*k*_+5_)*K*_9_(*k*_+12_)(*k*_-14_) 
         _+_ *K*_1_(*k*_+10_)(*k*_-2_)(*k*_+8_)(*k*_+4_)(*k*_-6_)(*k*_+5_)*K*_9_(*k*_-13_)(*k*_+15_) 
         _+_ *K*_1_(*k*_+10_)(*k*_-2_)(*k*_+8_)(*k*_+4_)(*k*_-6_)(*k*_+5_)*K*_9_(*k*_-13_)(*k*_-14_) 
         _+_ *K*_1_(*k*_+10_)(*k*_-2_)(*k*_+8_)(*k*_+4_)(*k*_-6_)(*k*_+5_)*K*_9_(*k*_+12_)(*k*_+15_) 
         _+_ *K*_1_(*k*_+10_)(*k*_-2_)(*k*_+8_)(*k*_+4_)(*k*_-6_)(*k*_+5_)*K*_9_(*k*_+12_)(*k*_-14_)}
β_16_ = {(*k*_-2_)(*k*_+8_)(*k*_+4_)(*k*_+7_)(*k*_+5_)*K*_9_(*k*_-10_)(*k*_-13_)(*k*_+15_) 
         _+_ (*k*_-2_)(*k*_+8_)(*k*_+4_)(*k*_+7_)(*k*_+5_)*K*_9_(*k*_-10_)(*k*_-13_)(*k*_-14_) 
         _+_ (*k*_-2_)(*k*_+8_)(*k*_+4_)(*k*_+7_)(*k*_+5_)*K*_9_(*k*_-10_)(*k*_+12_)(*k*_+15_) 
         _+_ (*k*_-2_)(*k*_+8_)(*k*_+4_)(*k*_+7_)(*k*_+5_)*K*_9_(*k*_-10_)(*k*_+12_)(*k*_-14_) 
         _+_ (*k*_-2_)(*k*_+8_)(*k*_+4_)(*k*_+7_)(*k*_+5_)*K*_9_(*k*_+11_)(*k*_-13_)(*k*_+15_) 
         _+_ (*k*_-2_)(*k*_+8_)(*k*_+4_)(*k*_+7_)(*k*_+5_)*K*_9_(*k*_+11_)(*k*_-13_)(*k*_-14_) 
         _+_ (*k*_-2_)(*k*_+8_)(*k*_+4_)(*k*_+7_)(*k*_+5_)*K*_9_(*k*_+11_)(*k*_+12_)(*k*_+15_) 
         _+_ (*k*_-2_)(*k*_+8_)(*k*_+4_)(*k*_+7_)(*k*_+5_)*K*_9_(*k*_+11_)(*k*_+12_)(*k*_-14_) 
         _+_ (*k*_-2_)(*k*_+8_)(*k*_+4_)(*k*_-6_)(*k*_+5_)*K*_9_(*k*_-10_)(*k*_-13_)(*k*_+15_) 
         _+_ (*k*_-2_)(*k*_+8_)(*k*_+4_)(*k*_-6_)(*k*_+5_)*K*_9_(*k*_-10_)(*k*_-13_)(*k*_-14_) 
         _+_ (*k*_-2_)(*k*_+8_)(*k*_+4_)(*k*_-6_)(*k*_+5_)*K*_9_(*k*_-10_)(*k*_+12_)(*k*_+15_) 
         _+_ (*k*_-2_)(*k*_+8_)(*k*_+4_)(*k*_-6_)(*k*_+5_)*K*_9_(*k*_-10_)(*k*_+12_)(*k*_-14_) 
         _+_ (*k*_-2_)(*k*_+8_)(*k*_+4_)(*k*_-6_)(*k*_+5_)*K*_9_(*k*_+11_)(*k*_-13_)(*k*_+15_) 
         _+_ (*k*_-2_)(*k*_+8_)(*k*_+4_)(*k*_-6_)(*k*_+5_)*K*_9_(*k*_+11_)(*k*_-13_)(*k*_-14_) 
         _+_ (*k*_-2_)(*k*_+8_)(*k*_+4_)(*k*_-6_)(*k*_+5_)*K*_9_(*k*_+11_)(*k*_+12_)(*k*_+15_) 
         _+_ (*k*_-2_)(*k*_+8_)(*k*_+4_)(*k*_-6_)(*k*_+5_)*K*_9_(*k*_+11_)(*k*_+12_)(*k*_-14_)}
β_17_ = {*K*_1_(*k*_+2_)(*k*_+8_)(*k*_+14_)(*k*_+7_)(*k*_+5_)*K*_9_(*k*_-10_)(*k*_-13_)(*k*_+15_) 
         _+_ *K*_1_(*k*_+2_)(*k*_+8_)(*k*_+14_)(*k*_+7_)(*k*_+5_)*K*_9_(*k*_-10_)(*k*_+12_)(*k*_+15_) 
         _+_ *K*_1_(*k*_+2_)(*k*_+8_)(*k*_+14_)(*k*_+7_)(*k*_+5_)*K*_9_(*k*_+11_)(*k*_-13_)(*k*_+15_) 
         _+_ *K*_1_(*k*_+2_)(*k*_+8_)(*k*_+14_)(*k*_+7_)(*k*_+5_)*K*_9_(*k*_+11_)(*k*_+12_)(*k*_+15_) 
         _+_ *K*_1_(*k*_+2_)(*k*_+8_)(*k*_+14_)(*k*_+7_)(*k*_-4_)*K*_9_(*k*_-10_)(*k*_-13_)(*k*_+15_) 
         _+_ *K*_1_(*k*_+2_)(*k*_+8_)(*k*_+14_)(*k*_+7_)(*k*_-4_)*K*_9_(*k*_-10_)(*k*_+12_)(*k*_+15_) 
         _+_ *K*_1_(*k*_+2_)(*k*_+8_)(*k*_+14_)(*k*_+7_)(*k*_-4_)*K*_9_(*k*_+11_)(*k*_-13_)(*k*_+15_) 
         _+_ *K*_1_(*k*_+2_)(*k*_+8_)(*k*_+14_)(*k*_+7_)(*k*_-4_)*K*_9_(*k*_+11_)(*k*_+12_)(*k*_+15_) 
         _+_ *K*_1_(*k*_+2_)(*k*_+8_)(*k*_+14_)(*k*_-6_)(*k*_+5_)*K*_9_(*k*_-10_)(*k*_-13_)(*k*_+15_) 
         _+_ *K*_1_(*k*_+2_)(*k*_+8_)(*k*_+14_)(*k*_-6_)(*k*_+5_)*K*_9_(*k*_-10_)(*k*_+12_)(*k*_+15_) 
         _+_ *K*_1_(*k*_+2_)(*k*_+8_)(*k*_+14_)(*k*_-6_)(*k*_+5_)*K*_9_(*k*_+11_)(*k*_-13_)(*k*_+15_) 
         _+_ *K*_1_(*k*_+2_)(*k*_+8_)(*k*_+14_)(*k*_-6_)(*k*_+5_)*K*_9_(*k*_+11_)(*k*_+12_)(*k*_+15_) 
         _+_ *K*_1_(*k*_+2_)(*k*_+8_)(*k*_+14_)(*k*_-6_)(*k*_-4_)*K*_9_(*k*_-10_)(*k*_-13_)(*k*_+15_) 
         _+_ *K*_1_(*k*_+2_)(*k*_+8_)(*k*_+14_)(*k*_-6_)(*k*_-4_)*K*_9_(*k*_-10_)(*k*_+12_)(*k*_+15_) 
         _+_ *K*_1_(*k*_+2_)(*k*_+8_)(*k*_+14_)(*k*_-6_)(*k*_-4_)*K*_9_(*k*_+11_)(*k*_-13_)(*k*_+15_) 
         _+_ *K*_1_(*k*_+2_)(*k*_+8_)(*k*_+14_)(*k*_-6_)(*k*_-4_)*K*_9_(*k*_+11_)(*k*_+12_)(*k*_+15_) 
         _+_ *K*_1_(*k*_+10_)(*k*_-2_)(*k*_+8_)(*k*_+6_)(*k*_+5_)*K*_9_(*k*_+11_)(*k*_-13_)(*k*_+15_) 
         _+_ *K*_1_(*k*_+10_)(*k*_-2_)(*k*_+8_)(*k*_+6_)(*k*_+5_)*K*_9_(*k*_+11_)(*k*_-13_)(*k*_-14_) 
         _+_ *K*_1_(*k*_+10_)(*k*_-2_)(*k*_+8_)(*k*_+6_)(*k*_+5_)*K*_9_(*k*_+11_)(*k*_+12_)(*k*_+15_) 
         _+_ *K*_1_(*k*_+10_)(*k*_-2_)(*k*_+8_)(*k*_+6_)(*k*_+5_)*K*_9_(*k*_+11_)(*k*_+12_)(*k*_-14_) 
         _+_ *K*_1_(*k*_+10_)(*k*_-2_)(*k*_+8_)(*k*_+6_)(*k*_-4_)*K*_9_(*k*_+11_)(*k*_-13_)(*k*_+15_) 
         _+_ *K*_1_(*k*_+10_)(*k*_-2_)(*k*_+8_)(*k*_+6_)(*k*_-4_)*K*_9_(*k*_+11_)(*k*_-13_)(*k*_-14_) 
         _+_ *K*_1_(*k*_+10_)(*k*_-2_)(*k*_+8_)(*k*_+6_)(*k*_-4_)*K*_9_(*k*_+11_)(*k*_+12_)(*k*_+15_) 
         _+_ *K*_1_(*k*_+10_)(*k*_-2_)(*k*_+8_)(*k*_+6_)(*k*_-4_)*K*_9_(*k*_+11_)(*k*_+12_)(*k*_-14_) 
         _+_ *K*_1_(*k*_+10_)(*k*_+3_)(*k*_+8_)(*k*_+6_)(*k*_+5_)*K*_9_(*k*_+11_)(*k*_-13_)(*k*_+15_) 
         _+_ *K*_1_(*k*_+10_)(*k*_+3_)(*k*_+8_)(*k*_+6_)(*k*_+5_)*K*_9_(*k*_+11_)(*k*_-13_)(*k*_-14_) 
         _+_ *K*_1_(*k*_+10_)(*k*_+3_)(*k*_+8_)(*k*_+6_)(*k*_+5_)*K*_9_(*k*_+11_)(*k*_+12_)(*k*_+15_) 
         _+_ *K*_1_(*k*_+10_)(*k*_+3_)(*k*_+8_)(*k*_+6_)(*k*_+5_)*K*_9_(*k*_+11_)(*k*_+12_)(*k*_-14_) 
         _+_ *K*_1_(*k*_+10_)(*k*_+3_)(*k*_+8_)(*k*_+6_)(*k*_-4_)*K*_9_(*k*_+11_)(*k*_-13_)(*k*_+15_) 
         _+_ *K*_1_(*k*_+10_)(*k*_+3_)(*k*_+8_)(*k*_+6_)(*k*_-4_)*K*_9_(*k*_+11_)(*k*_-13_)(*k*_-14_) 
         _+_ *K*_1_(*k*_+10_)(*k*_+3_)(*k*_+8_)(*k*_+6_)(*k*_-4_)*K*_9_(*k*_+11_)(*k*_+12_)(*k*_+15_) 
         _+_ *K*_1_(*k*_+10_)(*k*_+3_)(*k*_+8_)(*k*_+6_)(*k*_-4_)*K*_9_(*k*_+11_)(*k*_+12_)(*k*_-14_) 
         _+_ *K*_1_(*k*_+10_)(*k*_-2_)(*k*_+8_)(*k*_+6_)(*k*_+7_)(*k*_+5_)*K*_9_(*k*_-13_)(*k*_+15_) 
         _+_ *K*_1_(*k*_+10_)(*k*_-2_)(*k*_+8_)(*k*_+6_)(*k*_+7_)(*k*_+5_)*K*_9_(*k*_-13_)(*k*_-14_) 
         _+_ *K*_1_(*k*_+10_)(*k*_-2_)(*k*_+8_)(*k*_+6_)(*k*_+7_)(*k*_+5_)*K*_9_(*k*_+12_)(*k*_+15_) 
         _+_ *K*_1_(*k*_+10_)(*k*_-2_)(*k*_+8_)(*k*_+6_)(*k*_+7_)(*k*_+5_)*K*_9_(*k*_+12_)(*k*_-14_) 
         _+_ *K*_1_(*k*_+10_)(*k*_-2_)(*k*_+8_)(*k*_+6_)(*k*_+7_)(*k*_-4_)*K*_9_(*k*_-13_)(*k*_+15_) 
         _+_ *K*_1_(*k*_+10_)(*k*_-2_)(*k*_+8_)(*k*_+6_)(*k*_+7_)(*k*_-4_)*K*_9_(*k*_-13_)(*k*_-14_) 
         _+_ *K*_1_(*k*_+10_)(*k*_-2_)(*k*_+8_)(*k*_+6_)(*k*_+7_)(*k*_-4_)*K*_9_(*k*_+12_)(*k*_+15_) 
         _+_ *K*_1_(*k*_+10_)(*k*_-2_)(*k*_+8_)(*k*_+6_)(*k*_+7_)(*k*_-4_)*K*_9_(*k*_+12_)(*k*_-14_) 
         _+_ *K*_1_(*k*_+10_)(*k*_+3_)(*k*_+8_)(*k*_+6_)(*k*_+7_)(*k*_+5_)*K*_9_(*k*_-13_)(*k*_+15_) 
         _+_ *K*_1_(*k*_+10_)(*k*_+3_)(*k*_+8_)(*k*_+6_)(*k*_+7_)(*k*_+5_)*K*_9_(*k*_-13_)(*k*_-14_) 
         _+_ *K*_1_(*k*_+10_)(*k*_+3_)(*k*_+8_)(*k*_+6_)(*k*_+7_)(*k*_+5_)*K*_9_(*k*_+12_)(*k*_+15_) 
         _+_ *K*_1_(*k*_+10_)(*k*_+3_)(*k*_+8_)(*k*_+6_)(*k*_+7_)(*k*_+5_)*K*_9_(*k*_+12_)(*k*_-14_) 
         _+_ *K*_1_(*k*_+10_)(*k*_+3_)(*k*_+8_)(*k*_+6_)(*k*_+7_)(*k*_-4_)*K*_9_(*k*_-13_)(*k*_+15_) 
         _+_ *K*_1_(*k*_+10_)(*k*_+3_)(*k*_+8_)(*k*_+6_)(*k*_+7_)(*k*_-4_)*K*_9_(*k*_-13_)(*k*_-14_) 
         _+_ *K*_1_(*k*_+10_)(*k*_+3_)(*k*_+8_)(*k*_+6_)(*k*_+7_)(*k*_-4_)*K*_9_(*k*_+12_)(*k*_+15_) 
         _+_ *K*_1_(*k*_+10_)(*k*_+3_)(*k*_+8_)(*k*_+6_)(*k*_+7_)(*k*_-4_)*K*_9_(*k*_+12_)(*k*_-14_) 
         _+_ *K*_1_(*k*_+2_)(*k*_+3_)(*k*_+8_)(*k*_+14_)(*k*_+7_)(*k*_+5_)*K*_9_(*k*_-10_)(*k*_-13_) 
         _+_ *K*_1_(*k*_+2_)(*k*_+3_)(*k*_+8_)(*k*_+14_)(*k*_+7_)(*k*_+5_)*K*_9_(*k*_-10_)(*k*_+12_) 
         _+_ *K*_1_(*k*_+2_)(*k*_+3_)(*k*_+8_)(*k*_+14_)(*k*_+7_)(*k*_+5_)*K*_9_(*k*_+11_)(*k*_-13_) 
         _+_ *K*_1_(*k*_+2_)(*k*_+3_)(*k*_+8_)(*k*_+14_)(*k*_+7_)(*k*_+5_)*K*_9_(*k*_+11_)(*k*_+12_) 
         _+_ *K*_1_(*k*_+2_)(*k*_+3_)(*k*_+8_)(*k*_+14_)(*k*_+7_)(*k*_-4_)*K*_9_(*k*_-10_)(*k*_-13_) 
         _+_ *K*_1_(*k*_+2_)(*k*_+3_)(*k*_+8_)(*k*_+14_)(*k*_+7_)(*k*_-4_)*K*_9_(*k*_-10_)(*k*_+12_) 
         _+_ *K*_1_(*k*_+2_)(*k*_+3_)(*k*_+8_)(*k*_+14_)(*k*_+7_)(*k*_-4_)*K*_9_(*k*_+11_)(*k*_-13_) 
         _+_ *K*_1_(*k*_+2_)(*k*_+3_)(*k*_+8_)(*k*_+14_)(*k*_+7_)(*k*_-4_)*K*_9_(*k*_+11_)(*k*_+12_) 
         _+_ *K*_1_(*k*_+2_)(*k*_+3_)(*k*_+8_)(*k*_+14_)(*k*_-6_)(*k*_+5_)*K*_9_(*k*_-10_)(*k*_-13_) 
         _+_ *K*_1_(*k*_+2_)(*k*_+3_)(*k*_+8_)(*k*_+14_)(*k*_-6_)(*k*_+5_)*K*_9_(*k*_-10_)(*k*_+12_) 
         _+_ *K*_1_(*k*_+2_)(*k*_+3_)(*k*_+8_)(*k*_+14_)(*k*_-6_)(*k*_+5_)*K*_9_(*k*_+11_)(*k*_-13_) 
         _+_ *K*_1_(*k*_+2_)(*k*_+3_)(*k*_+8_)(*k*_+14_)(*k*_-6_)(*k*_+5_)*K*_9_(*k*_+11_)(*k*_+12_) 
         _+_ *K*_1_(*k*_+2_)(*k*_+3_)(*k*_+8_)(*k*_+14_)(*k*_-6_)(*k*_-4_)*K*_9_(*k*_-10_)(*k*_-13_) 
         _+_ *K*_1_(*k*_+2_)(*k*_+3_)(*k*_+8_)(*k*_+14_)(*k*_-6_)(*k*_-4_)*K*_9_(*k*_-10_)(*k*_+12_) 
         _+_ *K*_1_(*k*_+2_)(*k*_+3_)(*k*_+8_)(*k*_+14_)(*k*_-6_)(*k*_-4_)*K*_9_(*k*_+11_)(*k*_-13_) 
         _+_ *K*_1_(*k*_+2_)(*k*_+3_)(*k*_+8_)(*k*_+14_)(*k*_-6_)(*k*_-4_)*K*_9_(*k*_+11_)(*k*_+12_)}
β_18_ = {(*k*_-2_)(*k*_+8_)(*k*_+6_)(*k*_+7_)(*k*_+5_)*K*_9_(*k*_-10_)(*k*_-13_)(*k*_+15_) 
         _+_ (*k*_-2_)(*k*_+8_)(*k*_+6_)(*k*_+7_)(*k*_+5_)*K*_9_(*k*_-10_)(*k*_-13_)(*k*_-14_) 
         _+_ (*k*_-2_)(*k*_+8_)(*k*_+6_)(*k*_+7_)(*k*_+5_)*K*_9_(*k*_-10_)(*k*_+12_)(*k*_+15_) 
         _+_ (*k*_-2_)(*k*_+8_)(*k*_+6_)(*k*_+7_)(*k*_+5_)*K*_9_(*k*_-10_)(*k*_+12_)(*k*_-14_) 
         _+_ (*k*_-2_)(*k*_+8_)(*k*_+6_)(*k*_+7_)(*k*_+5_)*K*_9_(*k*_+11_)(*k*_-13_)(*k*_+15_) 
         _+_ (*k*_-2_)(*k*_+8_)(*k*_+6_)(*k*_+7_)(*k*_+5_)*K*_9_(*k*_+11_)(*k*_-13_)(*k*_-14_) 
         _+_ (*k*_-2_)(*k*_+8_)(*k*_+6_)(*k*_+7_)(*k*_+5_)*K*_9_(*k*_+11_)(*k*_+12_)(*k*_+15_) 
         _+_ (*k*_-2_)(*k*_+8_)(*k*_+6_)(*k*_+7_)(*k*_+5_)*K*_9_(*k*_+11_)(*k*_+12_)(*k*_-14_) 
         _+_ (*k*_-2_)(*k*_+8_)(*k*_+6_)(*k*_+7_)(*k*_-4_)*K*_9_(*k*_-10_)(*k*_-13_)(*k*_+15_) 
         _+_ (*k*_-2_)(*k*_+8_)(*k*_+6_)(*k*_+7_)(*k*_-4_)*K*_9_(*k*_-10_)(*k*_-13_)(*k*_-14_) 
         _+_ (*k*_-2_)(*k*_+8_)(*k*_+6_)(*k*_+7_)(*k*_-4_)*K*_9_(*k*_-10_)(*k*_+12_)(*k*_+15_) 
         _+_ (*k*_-2_)(*k*_+8_)(*k*_+6_)(*k*_+7_)(*k*_-4_)*K*_9_(*k*_-10_)(*k*_+12_)(*k*_-14_) 
         _+_ (*k*_-2_)(*k*_+8_)(*k*_+6_)(*k*_+7_)(*k*_-4_)*K*_9_(*k*_+11_)(*k*_-13_)(*k*_+15_) 
         _+_ (*k*_-2_)(*k*_+8_)(*k*_+6_)(*k*_+7_)(*k*_-4_)*K*_9_(*k*_+11_)(*k*_-13_)(*k*_-14_) 
         _+_ (*k*_-2_)(*k*_+8_)(*k*_+6_)(*k*_+7_)(*k*_-4_)*K*_9_(*k*_+11_)(*k*_+12_)(*k*_+15_) 
         _+_ (*k*_-2_)(*k*_+8_)(*k*_+6_)(*k*_+7_)(*k*_-4_)*K*_9_(*k*_+11_)(*k*_+12_)(*k*_-14_) 
         _+_ (*k*_+3_)(*k*_+8_)(*k*_+6_)(*k*_+7_)(*k*_+5_)*K*_9_(*k*_-10_)(*k*_-13_)(*k*_+15_) 
         _+_ (*k*_+3_)(*k*_+8_)(*k*_+6_)(*k*_+7_)(*k*_+5_)*K*_9_(*k*_-10_)(*k*_-13_)(*k*_-14_) 
         _+_ (*k*_+3_)(*k*_+8_)(*k*_+6_)(*k*_+7_)(*k*_+5_)*K*_9_(*k*_-10_)(*k*_+12_)(*k*_+15_) 
         _+_ (*k*_+3_)(*k*_+8_)(*k*_+6_)(*k*_+7_)(*k*_+5_)*K*_9_(*k*_-10_)(*k*_+12_)(*k*_-14_) 
         _+_ (*k*_+3_)(*k*_+8_)(*k*_+6_)(*k*_+7_)(*k*_+5_)*K*_9_(*k*_+11_)(*k*_-13_)(*k*_+15_) 
         _+_ (*k*_+3_)(*k*_+8_)(*k*_+6_)(*k*_+7_)(*k*_+5_)*K*_9_(*k*_+11_)(*k*_-13_)(*k*_-14_) 
         _+_ (*k*_+3_)(*k*_+8_)(*k*_+6_)(*k*_+7_)(*k*_+5_)*K*_9_(*k*_+11_)(*k*_+12_)(*k*_+15_) 
         _+_ (*k*_+3_)(*k*_+8_)(*k*_+6_)(*k*_+7_)(*k*_+5_)*K*_9_(*k*_+11_)(*k*_+12_)(*k*_-14_) 
         _+_ (*k*_+3_)(*k*_+8_)(*k*_+6_)(*k*_+7_)(*k*_-4_)*K*_9_(*k*_-10_)(*k*_-13_)(*k*_+15_) 
         _+_ (*k*_+3_)(*k*_+8_)(*k*_+6_)(*k*_+7_)(*k*_-4_)*K*_9_(*k*_-10_)(*k*_-13_)(*k*_-14_) 
         _+_ (*k*_+3_)(*k*_+8_)(*k*_+6_)(*k*_+7_)(*k*_-4_)*K*_9_(*k*_-10_)(*k*_+12_)(*k*_+15_) 
         _+_ (*k*_+3_)(*k*_+8_)(*k*_+6_)(*k*_+7_)(*k*_-4_)*K*_9_(*k*_-10_)(*k*_+12_)(*k*_-14_) 
         _+_ (*k*_+3_)(*k*_+8_)(*k*_+6_)(*k*_+7_)(*k*_-4_)*K*_9_(*k*_+11_)(*k*_-13_)(*k*_+15_) 
         _+_ (*k*_+3_)(*k*_+8_)(*k*_+6_)(*k*_+7_)(*k*_-4_)*K*_9_(*k*_+11_)(*k*_-13_)(*k*_-14_) 
         _+_ (*k*_+3_)(*k*_+8_)(*k*_+6_)(*k*_+7_)(*k*_-4_)*K*_9_(*k*_+11_)(*k*_+12_)(*k*_+15_) 
         _+_ (*k*_+3_)(*k*_+8_)(*k*_+6_)(*k*_+7_)(*k*_-4_)*K*_9_(*k*_+11_)(*k*_+12_)(*k*_-14_) 
         _+_ *K*_1_(*k*_+2_)(*k*_+8_)(*k*_+4_)(*k*_+7_)(*k*_+5_)*K*_9_(*k*_-10_)(*k*_-13_)(*k*_+15_) 
         _+_ *K*_1_(*k*_+2_)(*k*_+8_)(*k*_+4_)(*k*_+7_)(*k*_+5_)*K*_9_(*k*_-10_)(*k*_-13_)(*k*_-14_) 
         _+_ *K*_1_(*k*_+2_)(*k*_+8_)(*k*_+4_)(*k*_+7_)(*k*_+5_)*K*_9_(*k*_-10_)(*k*_+12_)(*k*_+15_) 
         _+_ *K*_1_(*k*_+2_)(*k*_+8_)(*k*_+4_)(*k*_+7_)(*k*_+5_)*K*_9_(*k*_-10_)(*k*_+12_)(*k*_-14_) 
         _+_ *K*_1_(*k*_+2_)(*k*_+8_)(*k*_+4_)(*k*_+7_)(*k*_+5_)*K*_9_(*k*_+11_)(*k*_-13_)(*k*_+15_) 
         _+_ *K*_1_(*k*_+2_)(*k*_+8_)(*k*_+4_)(*k*_+7_)(*k*_+5_)*K*_9_(*k*_+11_)(*k*_-13_)(*k*_-14_) 
         _+_ *K*_1_(*k*_+2_)(*k*_+8_)(*k*_+4_)(*k*_+7_)(*k*_+5_)*K*_9_(*k*_+11_)(*k*_+12_)(*k*_+15_) 
         _+_ *K*_1_(*k*_+2_)(*k*_+8_)(*k*_+4_)(*k*_+7_)(*k*_+5_)*K*_9_(*k*_+11_)(*k*_+12_)(*k*_-14_) 
         _+_ *K*_1_(*k*_+2_)(*k*_+8_)(*k*_+4_)(*k*_-6_)(*k*_+5_)*K*_9_(*k*_-10_)(*k*_-13_)(*k*_+15_) 
         _+_ *K*_1_(*k*_+2_)(*k*_+8_)(*k*_+4_)(*k*_-6_)(*k*_+5_)*K*_9_(*k*_-10_)(*k*_-13_)(*k*_-14_) 
         _+_ *K*_1_(*k*_+2_)(*k*_+8_)(*k*_+4_)(*k*_-6_)(*k*_+5_)*K*_9_(*k*_-10_)(*k*_+12_)(*k*_+15_) 
         _+_ *K*_1_(*k*_+2_)(*k*_+8_)(*k*_+4_)(*k*_-6_)(*k*_+5_)*K*_9_(*k*_-10_)(*k*_+12_)(*k*_-14_) 
         _+_ *K*_1_(*k*_+2_)(*k*_+8_)(*k*_+4_)(*k*_-6_)(*k*_+5_)*K*_9_(*k*_+11_)(*k*_-13_)(*k*_+15_) 
         _+_ *K*_1_(*k*_+2_)(*k*_+8_)(*k*_+4_)(*k*_-6_)(*k*_+5_)*K*_9_(*k*_+11_)(*k*_-13_)(*k*_-14_) 
         _+_ *K*_1_(*k*_+2_)(*k*_+8_)(*k*_+4_)(*k*_-6_)(*k*_+5_)*K*_9_(*k*_+11_)(*k*_+12_)(*k*_+15_) 
         _+_ *K*_1_(*k*_+2_)(*k*_+8_)(*k*_+4_)(*k*_-6_)(*k*_+5_)*K*_9_(*k*_+11_)(*k*_+12_)(*k*_-14_) 
         _+_ *K*_1_(*k*_+2_)(*k*_+3_)(*k*_+8_)(*k*_+4_)(*k*_+7_)*K*_9_(*k*_-10_)(*k*_-13_)(*k*_+15_) 
         _+_ *K*_1_(*k*_+2_)(*k*_+3_)(*k*_+8_)(*k*_+4_)(*k*_+7_)*K*_9_(*k*_-10_)(*k*_-13_)(*k*_-14_) 
         _+_ *K*_1_(*k*_+2_)(*k*_+3_)(*k*_+8_)(*k*_+4_)(*k*_+7_)*K*_9_(*k*_-10_)(*k*_+12_)(*k*_+15_) 
         _+_ *K*_1_(*k*_+2_)(*k*_+3_)(*k*_+8_)(*k*_+4_)(*k*_+7_)*K*_9_(*k*_-10_)(*k*_+12_)(*k*_-14_) 
         _+_ *K*_1_(*k*_+2_)(*k*_+3_)(*k*_+8_)(*k*_+4_)(*k*_+7_)*K*_9_(*k*_+11_)(*k*_-13_)(*k*_+15_) 
         _+_ *K*_1_(*k*_+2_)(*k*_+3_)(*k*_+8_)(*k*_+4_)(*k*_+7_)*K*_9_(*k*_+11_)(*k*_-13_)(*k*_-14_) 
         _+_ *K*_1_(*k*_+2_)(*k*_+3_)(*k*_+8_)(*k*_+4_)(*k*_+7_)*K*_9_(*k*_+11_)(*k*_+12_)(*k*_+15_) 
         _+_ *K*_1_(*k*_+2_)(*k*_+3_)(*k*_+8_)(*k*_+4_)(*k*_+7_)*K*_9_(*k*_+11_)(*k*_+12_)(*k*_-14_) 
         _+_ *K*_1_(*k*_+2_)(*k*_+3_)(*k*_+8_)(*k*_+4_)(*k*_-6_)*K*_9_(*k*_-10_)(*k*_-13_)(*k*_+15_) 
         _+_ *K*_1_(*k*_+2_)(*k*_+3_)(*k*_+8_)(*k*_+4_)(*k*_-6_)*K*_9_(*k*_-10_)(*k*_-13_)(*k*_-14_) 
         _+_ *K*_1_(*k*_+2_)(*k*_+3_)(*k*_+8_)(*k*_+4_)(*k*_-6_)*K*_9_(*k*_-10_)(*k*_+12_)(*k*_+15_) 
         _+_ *K*_1_(*k*_+2_)(*k*_+3_)(*k*_+8_)(*k*_+4_)(*k*_-6_)*K*_9_(*k*_-10_)(*k*_+12_)(*k*_-14_) 
         _+_ *K*_1_(*k*_+2_)(*k*_+3_)(*k*_+8_)(*k*_+4_)(*k*_-6_)*K*_9_(*k*_+11_)(*k*_-13_)(*k*_+15_) 
         _+_ *K*_1_(*k*_+2_)(*k*_+3_)(*k*_+8_)(*k*_+4_)(*k*_-6_)*K*_9_(*k*_+11_)(*k*_-13_)(*k*_-14_) 
         _+_ *K*_1_(*k*_+2_)(*k*_+3_)(*k*_+8_)(*k*_+4_)(*k*_-6_)*K*_9_(*k*_+11_)(*k*_+12_)(*k*_+15_) 
         _+_ *K*_1_(*k*_+2_)(*k*_+3_)(*k*_+8_)(*k*_+4_)(*k*_-6_)*K*_9_(*k*_+11_)(*k*_+12_)(*k*_-14_)}
β_19_ = {*K*_1_(*k*_+2_)(*k*_+8_)(*k*_+6_)(*k*_+7_)(*k*_+5_)*K*_9_(*k*_-10_)(*k*_-13_)(*k*_+15_) 
         _+_ *K*_1_(*k*_+2_)(*k*_+8_)(*k*_+6_)(*k*_+7_)(*k*_+5_)*K*_9_(*k*_-10_)(*k*_-13_)(*k*_-14_) 
         _+_ *K*_1_(*k*_+2_)(*k*_+8_)(*k*_+6_)(*k*_+7_)(*k*_+5_)*K*_9_(*k*_-10_)(*k*_+12_)(*k*_+15_) 
         _+_ *K*_1_(*k*_+2_)(*k*_+8_)(*k*_+6_)(*k*_+7_)(*k*_+5_)*K*_9_(*k*_-10_)(*k*_+12_)(*k*_-14_) 
         _+_ *K*_1_(*k*_+2_)(*k*_+8_)(*k*_+6_)(*k*_+7_)(*k*_+5_)*K*_9_(*k*_+11_)(*k*_-13_)(*k*_+15_) 
         _+_ *K*_1_(*k*_+2_)(*k*_+8_)(*k*_+6_)(*k*_+7_)(*k*_+5_)*K*_9_(*k*_+11_)(*k*_-13_)(*k*_-14_) 
         _+_ *K*_1_(*k*_+2_)(*k*_+8_)(*k*_+6_)(*k*_+7_)(*k*_+5_)*K*_9_(*k*_+11_)(*k*_+12_)(*k*_+15_) 
         _+_ *K*_1_(*k*_+2_)(*k*_+8_)(*k*_+6_)(*k*_+7_)(*k*_+5_)*K*_9_(*k*_+11_)(*k*_+12_)(*k*_-14_) 
         _+_ *K*_1_(*k*_+2_)(*k*_+8_)(*k*_+6_)(*k*_+7_)(*k*_-4_)*K*_9_(*k*_-10_)(*k*_-13_)(*k*_+15_) 
         _+_ *K*_1_(*k*_+2_)(*k*_+8_)(*k*_+6_)(*k*_+7_)(*k*_-4_)*K*_9_(*k*_-10_)(*k*_-13_)(*k*_-14_) 
         _+_ *K*_1_(*k*_+2_)(*k*_+8_)(*k*_+6_)(*k*_+7_)(*k*_-4_)*K*_9_(*k*_-10_)(*k*_+12_)(*k*_+15_) 
         _+_ *K*_1_(*k*_+2_)(*k*_+8_)(*k*_+6_)(*k*_+7_)(*k*_-4_)*K*_9_(*k*_-10_)(*k*_+12_)(*k*_-14_) 
         _+_ *K*_1_(*k*_+2_)(*k*_+8_)(*k*_+6_)(*k*_+7_)(*k*_-4_)*K*_9_(*k*_+11_)(*k*_-13_)(*k*_+15_) 
         _+_ *K*_1_(*k*_+2_)(*k*_+8_)(*k*_+6_)(*k*_+7_)(*k*_-4_)*K*_9_(*k*_+11_)(*k*_-13_)(*k*_-14_) 
         _+_ *K*_1_(*k*_+2_)(*k*_+8_)(*k*_+6_)(*k*_+7_)(*k*_-4_)*K*_9_(*k*_+11_)(*k*_+12_)(*k*_+15_) 
         _+_ *K*_1_(*k*_+2_)(*k*_+8_)(*k*_+6_)(*k*_+7_)(*k*_-4_)*K*_9_(*k*_+11_)(*k*_+12_)(*k*_-14_) 
         _+_ *K*_1_(*k*_+2_)(*k*_+3_)(*k*_+8_)(*k*_+6_)(*k*_+5_)*K*_9_(*k*_-10_)(*k*_-13_)(*k*_+15_) 
         _+_ *K*_1_(*k*_+2_)(*k*_+3_)(*k*_+8_)(*k*_+6_)(*k*_+5_)*K*_9_(*k*_-10_)(*k*_-13_)(*k*_-14_) 
         _+_ *K*_1_(*k*_+2_)(*k*_+3_)(*k*_+8_)(*k*_+6_)(*k*_+5_)*K*_9_(*k*_-10_)(*k*_+12_)(*k*_+15_) 
         _+_ *K*_1_(*k*_+2_)(*k*_+3_)(*k*_+8_)(*k*_+6_)(*k*_+5_)*K*_9_(*k*_-10_)(*k*_+12_)(*k*_-14_) 
         _+_ *K*_1_(*k*_+2_)(*k*_+3_)(*k*_+8_)(*k*_+6_)(*k*_+5_)*K*_9_(*k*_+11_)(*k*_-13_)(*k*_+15_) 
         _+_ *K*_1_(*k*_+2_)(*k*_+3_)(*k*_+8_)(*k*_+6_)(*k*_+5_)*K*_9_(*k*_+11_)(*k*_-13_)(*k*_-14_) 
         _+_ *K*_1_(*k*_+2_)(*k*_+3_)(*k*_+8_)(*k*_+6_)(*k*_+5_)*K*_9_(*k*_+11_)(*k*_+12_)(*k*_+15_) 
         _+_ *K*_1_(*k*_+2_)(*k*_+3_)(*k*_+8_)(*k*_+6_)(*k*_+5_)*K*_9_(*k*_+11_)(*k*_+12_)(*k*_-14_) 
         _+_ *K*_1_(*k*_+2_)(*k*_+3_)(*k*_+8_)(*k*_+6_)(*k*_-4_)*K*_9_(*k*_-10_)(*k*_-13_)(*k*_+15_) 
         _+_ *K*_1_(*k*_+2_)(*k*_+3_)(*k*_+8_)(*k*_+6_)(*k*_-4_)*K*_9_(*k*_-10_)(*k*_-13_)(*k*_-14_) 
         _+_ *K*_1_(*k*_+2_)(*k*_+3_)(*k*_+8_)(*k*_+6_)(*k*_-4_)*K*_9_(*k*_-10_)(*k*_+12_)(*k*_+15_) 
         _+_ *K*_1_(*k*_+2_)(*k*_+3_)(*k*_+8_)(*k*_+6_)(*k*_-4_)*K*_9_(*k*_-10_)(*k*_+12_)(*k*_-14_) 
         _+_ *K*_1_(*k*_+2_)(*k*_+3_)(*k*_+8_)(*k*_+6_)(*k*_-4_)*K*_9_(*k*_+11_)(*k*_-13_)(*k*_+15_) 
         _+_ *K*_1_(*k*_+2_)(*k*_+3_)(*k*_+8_)(*k*_+6_)(*k*_-4_)*K*_9_(*k*_+11_)(*k*_-13_)(*k*_-14_) 
         _+_ *K*_1_(*k*_+2_)(*k*_+3_)(*k*_+8_)(*k*_+6_)(*k*_-4_)*K*_9_(*k*_+11_)(*k*_+12_)(*k*_+15_) 
         _+_ *K*_1_(*k*_+2_)(*k*_+3_)(*k*_+8_)(*k*_+6_)(*k*_-4_)*K*_9_(*k*_+11_)(*k*_+12_)(*k*_-14_)}
β_20_ = {*K*_1_(*k*_-2_)(*k*_+8_)(*k*_+14_)(*k*_+7_)(*k*_+5_)(*k*_-10_)(*k*_-13_)(*k*_+15_) 
         _+_ *K*_1_(*k*_-2_)(*k*_+8_)(*k*_+14_)(*k*_+7_)(*k*_+5_)(*k*_-10_)(*k*_+12_)(*k*_+15_) 
         _+_ *K*_1_(*k*_-2_)(*k*_+8_)(*k*_+14_)(*k*_+7_)(*k*_+5_)(*k*_+11_)(*k*_-13_)(*k*_+15_) 
         _+_ *K*_1_(*k*_-2_)(*k*_+8_)(*k*_+14_)(*k*_+7_)(*k*_+5_)(*k*_+11_)(*k*_+12_)(*k*_+15_) 
         _+_ *K*_1_(*k*_-2_)(*k*_+8_)(*k*_+14_)(*k*_+7_)(*k*_-4_)(*k*_-10_)(*k*_-13_)(*k*_+15_) 
         _+_ *K*_1_(*k*_-2_)(*k*_+8_)(*k*_+14_)(*k*_+7_)(*k*_-4_)(*k*_-10_)(*k*_+12_)(*k*_+15_) 
         _+_ *K*_1_(*k*_-2_)(*k*_+8_)(*k*_+14_)(*k*_+7_)(*k*_-4_)(*k*_+11_)(*k*_-13_)(*k*_+15_) 
         _+_ *K*_1_(*k*_-2_)(*k*_+8_)(*k*_+14_)(*k*_+7_)(*k*_-4_)(*k*_+11_)(*k*_+12_)(*k*_+15_) 
         _+_ *K*_1_(*k*_-2_)(*k*_+8_)(*k*_+14_)(*k*_-6_)(*k*_+5_)(*k*_-10_)(*k*_-13_)(*k*_+15_) 
         _+_ *K*_1_(*k*_-2_)(*k*_+8_)(*k*_+14_)(*k*_-6_)(*k*_+5_)(*k*_-10_)(*k*_+12_)(*k*_+15_) 
         _+_ *K*_1_(*k*_-2_)(*k*_+8_)(*k*_+14_)(*k*_-6_)(*k*_+5_)(*k*_+11_)(*k*_-13_)(*k*_+15_) 
         _+_ *K*_1_(*k*_-2_)(*k*_+8_)(*k*_+14_)(*k*_-6_)(*k*_+5_)(*k*_+11_)(*k*_+12_)(*k*_+15_) 
         _+_ *K*_1_(*k*_-2_)(*k*_+8_)(*k*_+14_)(*k*_-6_)(*k*_-4_)(*k*_-10_)(*k*_-13_)(*k*_+15_) 
         _+_ *K*_1_(*k*_-2_)(*k*_+8_)(*k*_+14_)(*k*_-6_)(*k*_-4_)(*k*_-10_)(*k*_+12_)(*k*_+15_) 
         _+_ *K*_1_(*k*_-2_)(*k*_+8_)(*k*_+14_)(*k*_-6_)(*k*_-4_)(*k*_+11_)(*k*_-13_)(*k*_+15_) 
         _+_ *K*_1_(*k*_-2_)(*k*_+8_)(*k*_+14_)(*k*_-6_)(*k*_-4_)(*k*_+11_)(*k*_+12_)(*k*_+15_) 
         _+_ *K*_1_(*k*_+3_)(*k*_+8_)(*k*_+14_)(*k*_+7_)(*k*_+5_)(*k*_-10_)(*k*_-13_)(*k*_+15_) 
         _+_ *K*_1_(*k*_+3_)(*k*_+8_)(*k*_+14_)(*k*_+7_)(*k*_+5_)(*k*_-10_)(*k*_+12_)(*k*_+15_) 
         _+_ *K*_1_(*k*_+3_)(*k*_+8_)(*k*_+14_)(*k*_+7_)(*k*_+5_)(*k*_+11_)(*k*_-13_)(*k*_+15_) 
         _+_ *K*_1_(*k*_+3_)(*k*_+8_)(*k*_+14_)(*k*_+7_)(*k*_+5_)(*k*_+11_)(*k*_+12_)(*k*_+15_) 
         _+_ *K*_1_(*k*_+3_)(*k*_+8_)(*k*_+14_)(*k*_+7_)(*k*_-4_)(*k*_-10_)(*k*_-13_)(*k*_+15_) 
         _+_ *K*_1_(*k*_+3_)(*k*_+8_)(*k*_+14_)(*k*_+7_)(*k*_-4_)(*k*_-10_)(*k*_+12_)(*k*_+15_) 
         _+_ *K*_1_(*k*_+3_)(*k*_+8_)(*k*_+14_)(*k*_+7_)(*k*_-4_)(*k*_+11_)(*k*_-13_)(*k*_+15_) 
         _+_ *K*_1_(*k*_+3_)(*k*_+8_)(*k*_+14_)(*k*_+7_)(*k*_-4_)(*k*_+11_)(*k*_+12_)(*k*_+15_) 
         _+_ *K*_1_(*k*_+3_)(*k*_+8_)(*k*_+14_)(*k*_-6_)(*k*_+5_)(*k*_-10_)(*k*_-13_)(*k*_+15_) 
         _+_ *K*_1_(*k*_+3_)(*k*_+8_)(*k*_+14_)(*k*_-6_)(*k*_+5_)(*k*_-10_)(*k*_+12_)(*k*_+15_) 
         _+_ *K*_1_(*k*_+3_)(*k*_+8_)(*k*_+14_)(*k*_-6_)(*k*_+5_)(*k*_+11_)(*k*_-13_)(*k*_+15_) 
         _+_ *K*_1_(*k*_+3_)(*k*_+8_)(*k*_+14_)(*k*_-6_)(*k*_+5_)(*k*_+11_)(*k*_+12_)(*k*_+15_) 
         _+_ *K*_1_(*k*_+3_)(*k*_+8_)(*k*_+14_)(*k*_-6_)(*k*_-4_)(*k*_-10_)(*k*_-13_)(*k*_+15_) 
         _+_ *K*_1_(*k*_+3_)(*k*_+8_)(*k*_+14_)(*k*_-6_)(*k*_-4_)(*k*_-10_)(*k*_+12_)(*k*_+15_) 
         _+_ *K*_1_(*k*_+3_)(*k*_+8_)(*k*_+14_)(*k*_-6_)(*k*_-4_)(*k*_+11_)(*k*_-13_)(*k*_+15_) 
         _+_ *K*_1_(*k*_+3_)(*k*_+8_)(*k*_+14_)(*k*_-6_)(*k*_-4_)(*k*_+11_)(*k*_+12_)(*k*_+15_) 
         _+_ *K*_1_(*k*_+10_)(*k*_-2_)(*k*_+8_)(*k*_+13_)(*k*_+7_)(*k*_+5_)*K*_9_(*k*_+12_)(*k*_+15_) 
         _+_ *K*_1_(*k*_+10_)(*k*_-2_)(*k*_+8_)(*k*_+13_)(*k*_+7_)(*k*_+5_)*K*_9_(*k*_+12_)(*k*_-14_) 
         _+_ *K*_1_(*k*_+10_)(*k*_-2_)(*k*_+8_)(*k*_+13_)(*k*_+7_)(*k*_-4_)*K*_9_(*k*_+12_)(*k*_+15_) 
         _+_ *K*_1_(*k*_+10_)(*k*_-2_)(*k*_+8_)(*k*_+13_)(*k*_+7_)(*k*_-4_)*K*_9_(*k*_+12_)(*k*_-14_) 
         _+_ *K*_1_(*k*_+10_)(*k*_-2_)(*k*_+8_)(*k*_+13_)(*k*_-6_)(*k*_+5_)*K*_9_(*k*_+12_)(*k*_+15_) 
         _+_ *K*_1_(*k*_+10_)(*k*_-2_)(*k*_+8_)(*k*_+13_)(*k*_-6_)(*k*_+5_)*K*_9_(*k*_+12_)(*k*_-14_) 
         _+_ *K*_1_(*k*_+10_)(*k*_-2_)(*k*_+8_)(*k*_+13_)(*k*_-6_)(*k*_-4_)*K*_9_(*k*_+12_)(*k*_+15_) 
         _+_ *K*_1_(*k*_+10_)(*k*_-2_)(*k*_+8_)(*k*_+13_)(*k*_-6_)(*k*_-4_)*K*_9_(*k*_+12_)(*k*_-14_) 
         _+_ *K*_1_(*k*_+10_)(*k*_+3_)(*k*_+8_)(*k*_+13_)(*k*_+7_)(*k*_+5_)*K*_9_(*k*_+12_)(*k*_+15_) 
         _+_ *K*_1_(*k*_+10_)(*k*_+3_)(*k*_+8_)(*k*_+13_)(*k*_+7_)(*k*_+5_)*K*_9_(*k*_+12_)(*k*_-14_) 
         _+_ *K*_1_(*k*_+10_)(*k*_+3_)(*k*_+8_)(*k*_+13_)(*k*_+7_)(*k*_-4_)*K*_9_(*k*_+12_)(*k*_+15_) 
         _+_ *K*_1_(*k*_+10_)(*k*_+3_)(*k*_+8_)(*k*_+13_)(*k*_+7_)(*k*_-4_)*K*_9_(*k*_+12_)(*k*_-14_) 
         _+_ *K*_1_(*k*_+10_)(*k*_+3_)(*k*_+8_)(*k*_+13_)(*k*_-6_)(*k*_+5_)*K*_9_(*k*_+12_)(*k*_+15_) 
         _+_ *K*_1_(*k*_+10_)(*k*_+3_)(*k*_+8_)(*k*_+13_)(*k*_-6_)(*k*_+5_)*K*_9_(*k*_+12_)(*k*_-14_) 
         _+_ *K*_1_(*k*_+10_)(*k*_+3_)(*k*_+8_)(*k*_+13_)(*k*_-6_)(*k*_-4_)*K*_9_(*k*_+12_)(*k*_+15_) 
         _+_ *K*_1_(*k*_+10_)(*k*_+3_)(*k*_+8_)(*k*_+13_)(*k*_-6_)(*k*_-4_)*K*_9_(*k*_+12_)(*k*_-14_) 
         _+_ *K*_1_(*k*_+10_)(*k*_-2_)(*k*_+8_)(*k*_+13_)(*k*_+7_)(*k*_+5_)*K*_9_(*k*_+11_)(*k*_+15_) 
         _+_ *K*_1_(*k*_+10_)(*k*_-2_)(*k*_+8_)(*k*_+13_)(*k*_+7_)(*k*_+5_)*K*_9_(*k*_+11_)(*k*_-14_) 
         _+_ *K*_1_(*k*_+10_)(*k*_-2_)(*k*_+8_)(*k*_+13_)(*k*_+7_)(*k*_-4_)*K*_9_(*k*_+11_)(*k*_+15_) 
         _+_ *K*_1_(*k*_+10_)(*k*_-2_)(*k*_+8_)(*k*_+13_)(*k*_+7_)(*k*_-4_)*K*_9_(*k*_+11_)(*k*_-14_) 
         _+_ *K*_1_(*k*_+10_)(*k*_-2_)(*k*_+8_)(*k*_+13_)(*k*_-6_)(*k*_+5_)*K*_9_(*k*_+11_)(*k*_+15_) 
         _+_ *K*_1_(*k*_+10_)(*k*_-2_)(*k*_+8_)(*k*_+13_)(*k*_-6_)(*k*_+5_)*K*_9_(*k*_+11_)(*k*_-14_) 
         _+_ *K*_1_(*k*_+10_)(*k*_-2_)(*k*_+8_)(*k*_+13_)(*k*_-6_)(*k*_-4_)*K*_9_(*k*_+11_)(*k*_+15_) 
         _+_ *K*_1_(*k*_+10_)(*k*_-2_)(*k*_+8_)(*k*_+13_)(*k*_-6_)(*k*_-4_)*K*_9_(*k*_+11_)(*k*_-14_) 
         _+_ *K*_1_(*k*_+10_)(*k*_+3_)(*k*_+8_)(*k*_+13_)(*k*_+7_)(*k*_+5_)*K*_9_(*k*_+11_)(*k*_+15_) 
         _+_ *K*_1_(*k*_+10_)(*k*_+3_)(*k*_+8_)(*k*_+13_)(*k*_+7_)(*k*_+5_)*K*_9_(*k*_+11_)(*k*_-14_) 
         _+_ *K*_1_(*k*_+10_)(*k*_+3_)(*k*_+8_)(*k*_+13_)(*k*_+7_)(*k*_-4_)*K*_9_(*k*_+11_)(*k*_+15_) 
         _+_ *K*_1_(*k*_+10_)(*k*_+3_)(*k*_+8_)(*k*_+13_)(*k*_+7_)(*k*_-4_)*K*_9_(*k*_+11_)(*k*_-14_) 
         _+_ *K*_1_(*k*_+10_)(*k*_+3_)(*k*_+8_)(*k*_+13_)(*k*_-6_)(*k*_+5_)*K*_9_(*k*_+11_)(*k*_+15_) 
         _+_ *K*_1_(*k*_+10_)(*k*_+3_)(*k*_+8_)(*k*_+13_)(*k*_-6_)(*k*_+5_)*K*_9_(*k*_+11_)(*k*_-14_) 
         _+_ *K*_1_(*k*_+10_)(*k*_+3_)(*k*_+8_)(*k*_+13_)(*k*_-6_)(*k*_-4_)*K*_9_(*k*_+11_)(*k*_+15_) 
         _+_ *K*_1_(*k*_+10_)(*k*_+3_)(*k*_+8_)(*k*_+13_)(*k*_-6_)(*k*_-4_)*K*_9_(*k*_+11_)(*k*_-14_)}
β_21_ = {(*k*_-2_)(*k*_+8_)(*k*_+13_)(*k*_+7_)(*k*_+5_)*K*_9_(*k*_-10_)(*k*_+12_)(*k*_+15_) 
         _+_ (*k*_-2_)(*k*_+8_)(*k*_+13_)(*k*_+7_)(*k*_+5_)*K*_9_(*k*_-10_)(*k*_+12_)(*k*_-14_) 
         _+_ (*k*_-2_)(*k*_+8_)(*k*_+13_)(*k*_+7_)(*k*_-4_)*K*_9_(*k*_-10_)(*k*_+12_)(*k*_+15_) 
         _+_ (*k*_-2_)(*k*_+8_)(*k*_+13_)(*k*_+7_)(*k*_-4_)*K*_9_(*k*_-10_)(*k*_+12_)(*k*_-14_) 
         _+_ (*k*_-2_)(*k*_+8_)(*k*_+13_)(*k*_-6_)(*k*_+5_)*K*_9_(*k*_-10_)(*k*_+12_)(*k*_+15_) 
         _+_ (*k*_-2_)(*k*_+8_)(*k*_+13_)(*k*_-6_)(*k*_+5_)*K*_9_(*k*_-10_)(*k*_+12_)(*k*_-14_) 
         _+_ (*k*_-2_)(*k*_+8_)(*k*_+13_)(*k*_-6_)(*k*_-4_)*K*_9_(*k*_-10_)(*k*_+12_)(*k*_+15_) 
         _+_ (*k*_-2_)(*k*_+8_)(*k*_+13_)(*k*_-6_)(*k*_-4_)*K*_9_(*k*_-10_)(*k*_+12_)(*k*_-14_) 
         _+_ (*k*_+3_)(*k*_+8_)(*k*_+13_)(*k*_+7_)(*k*_+5_)*K*_9_(*k*_-10_)(*k*_+12_)(*k*_+15_) 
         _+_ (*k*_+3_)(*k*_+8_)(*k*_+13_)(*k*_+7_)(*k*_+5_)*K*_9_(*k*_-10_)(*k*_+12_)(*k*_-14_) 
         _+_ (*k*_+3_)(*k*_+8_)(*k*_+13_)(*k*_+7_)(*k*_-4_)*K*_9_(*k*_-10_)(*k*_+12_)(*k*_+15_) 
         _+_ (*k*_+3_)(*k*_+8_)(*k*_+13_)(*k*_+7_)(*k*_-4_)*K*_9_(*k*_-10_)(*k*_+12_)(*k*_-14_) 
         _+_ (*k*_+3_)(*k*_+8_)(*k*_+13_)(*k*_-6_)(*k*_+5_)*K*_9_(*k*_-10_)(*k*_+12_)(*k*_+15_) 
         _+_ (*k*_+3_)(*k*_+8_)(*k*_+13_)(*k*_-6_)(*k*_+5_)*K*_9_(*k*_-10_)(*k*_+12_)(*k*_-14_) 
         _+_ (*k*_+3_)(*k*_+8_)(*k*_+13_)(*k*_-6_)(*k*_-4_)*K*_9_(*k*_-10_)(*k*_+12_)(*k*_+15_) 
         _+_ (*k*_+3_)(*k*_+8_)(*k*_+13_)(*k*_-6_)(*k*_-4_)*K*_9_(*k*_-10_)(*k*_+12_)(*k*_-14_) 
         _+_ *K*_1_(*k*_-2_)(*k*_+8_)(*k*_+4_)(*k*_+7_)(*k*_+5_)(*k*_-10_)(*k*_-13_)(*k*_+15_) 
         _+_ *K*_1_(*k*_-2_)(*k*_+8_)(*k*_+4_)(*k*_+7_)(*k*_+5_)(*k*_-10_)(*k*_-13_)(*k*_-14_) 
         _+_ *K*_1_(*k*_-2_)(*k*_+8_)(*k*_+4_)(*k*_+7_)(*k*_+5_)(*k*_-10_)(*k*_+12_)(*k*_+15_) 
         _+_ *K*_1_(*k*_-2_)(*k*_+8_)(*k*_+4_)(*k*_+7_)(*k*_+5_)(*k*_-10_)(*k*_+12_)(*k*_-14_) 
         _+_ *K*_1_(*k*_-2_)(*k*_+8_)(*k*_+4_)(*k*_+7_)(*k*_+5_)(*k*_+11_)(*k*_-13_)(*k*_+15_) 
         _+_ *K*_1_(*k*_-2_)(*k*_+8_)(*k*_+4_)(*k*_+7_)(*k*_+5_)(*k*_+11_)(*k*_-13_)(*k*_-14_) 
         _+_ *K*_1_(*k*_-2_)(*k*_+8_)(*k*_+4_)(*k*_+7_)(*k*_+5_)(*k*_+11_)(*k*_+12_)(*k*_+15_) 
         _+_ *K*_1_(*k*_-2_)(*k*_+8_)(*k*_+4_)(*k*_+7_)(*k*_+5_)(*k*_+11_)(*k*_+12_)(*k*_-14_) 
         _+_ *K*_1_(*k*_-2_)(*k*_+8_)(*k*_+4_)(*k*_-6_)(*k*_+5_)(*k*_-10_)(*k*_-13_)(*k*_+15_) 
         _+_ *K*_1_(*k*_-2_)(*k*_+8_)(*k*_+4_)(*k*_-6_)(*k*_+5_)(*k*_-10_)(*k*_-13_)(*k*_-14_) 
         _+_ *K*_1_(*k*_-2_)(*k*_+8_)(*k*_+4_)(*k*_-6_)(*k*_+5_)(*k*_-10_)(*k*_+12_)(*k*_+15_) 
         _+_ *K*_1_(*k*_-2_)(*k*_+8_)(*k*_+4_)(*k*_-6_)(*k*_+5_)(*k*_-10_)(*k*_+12_)(*k*_-14_) 
         _+_ *K*_1_(*k*_-2_)(*k*_+8_)(*k*_+4_)(*k*_-6_)(*k*_+5_)(*k*_+11_)(*k*_-13_)(*k*_+15_) 
         _+_ *K*_1_(*k*_-2_)(*k*_+8_)(*k*_+4_)(*k*_-6_)(*k*_+5_)(*k*_+11_)(*k*_-13_)(*k*_-14_) 
         _+_ *K*_1_(*k*_-2_)(*k*_+8_)(*k*_+4_)(*k*_-6_)(*k*_+5_)(*k*_+11_)(*k*_+12_)(*k*_+15_) 
         _+_ *K*_1_(*k*_-2_)(*k*_+8_)(*k*_+4_)(*k*_-6_)(*k*_+5_)(*k*_+11_)(*k*_+12_)(*k*_-14_)}
β_22_ = {*K*_1_(*k*_+2_)(*k*_+8_)(*k*_+13_)(*k*_+7_)(*k*_+5_)*K*_9_(*k*_-10_)(*k*_+12_)(*k*_+15_) 
         _+_ *K*_1_(*k*_+2_)(*k*_+8_)(*k*_+13_)(*k*_+7_)(*k*_+5_)*K*_9_(*k*_-10_)(*k*_+12_)(*k*_-14_) 
         _+_ *K*_1_(*k*_+2_)(*k*_+8_)(*k*_+13_)(*k*_+7_)(*k*_-4_)*K*_9_(*k*_-10_)(*k*_+12_)(*k*_+15_) 
         _+_ *K*_1_(*k*_+2_)(*k*_+8_)(*k*_+13_)(*k*_+7_)(*k*_-4_)*K*_9_(*k*_-10_)(*k*_+12_)(*k*_-14_) 
         _+_ *K*_1_(*k*_+2_)(*k*_+8_)(*k*_+13_)(*k*_-6_)(*k*_+5_)*K*_9_(*k*_-10_)(*k*_+12_)(*k*_+15_) 
         _+_ *K*_1_(*k*_+2_)(*k*_+8_)(*k*_+13_)(*k*_-6_)(*k*_+5_)*K*_9_(*k*_-10_)(*k*_+12_)(*k*_-14_) 
         _+_ *K*_1_(*k*_+2_)(*k*_+8_)(*k*_+13_)(*k*_-6_)(*k*_-4_)*K*_9_(*k*_-10_)(*k*_+12_)(*k*_+15_) 
         _+_ *K*_1_(*k*_+2_)(*k*_+8_)(*k*_+13_)(*k*_-6_)(*k*_-4_)*K*_9_(*k*_-10_)(*k*_+12_)(*k*_-14_) 
         _+_ *K*_1_(*k*_-2_)(*k*_+8_)(*k*_+6_)(*k*_+7_)(*k*_+5_)(*k*_-10_)(*k*_-13_)(*k*_+15_) 
         _+_ *K*_1_(*k*_-2_)(*k*_+8_)(*k*_+6_)(*k*_+7_)(*k*_+5_)(*k*_-10_)(*k*_-13_)(*k*_-14_) 
         _+_ *K*_1_(*k*_-2_)(*k*_+8_)(*k*_+6_)(*k*_+7_)(*k*_+5_)(*k*_-10_)(*k*_+12_)(*k*_+15_) 
         _+_ *K*_1_(*k*_-2_)(*k*_+8_)(*k*_+6_)(*k*_+7_)(*k*_+5_)(*k*_-10_)(*k*_+12_)(*k*_-14_) 
         _+_ *K*_1_(*k*_-2_)(*k*_+8_)(*k*_+6_)(*k*_+7_)(*k*_+5_)(*k*_+11_)(*k*_-13_)(*k*_+15_) 
         _+_ *K*_1_(*k*_-2_)(*k*_+8_)(*k*_+6_)(*k*_+7_)(*k*_+5_)(*k*_+11_)(*k*_-13_)(*k*_-14_) 
         _+_ *K*_1_(*k*_-2_)(*k*_+8_)(*k*_+6_)(*k*_+7_)(*k*_+5_)(*k*_+11_)(*k*_+12_)(*k*_+15_) 
         _+_ *K*_1_(*k*_-2_)(*k*_+8_)(*k*_+6_)(*k*_+7_)(*k*_+5_)(*k*_+11_)(*k*_+12_)(*k*_-14_) 
         _+_ *K*_1_(*k*_-2_)(*k*_+8_)(*k*_+6_)(*k*_+7_)(*k*_-4_)(*k*_-10_)(*k*_-13_)(*k*_+15_) 
         _+_ *K*_1_(*k*_-2_)(*k*_+8_)(*k*_+6_)(*k*_+7_)(*k*_-4_)(*k*_-10_)(*k*_-13_)(*k*_-14_) 
         _+_ *K*_1_(*k*_-2_)(*k*_+8_)(*k*_+6_)(*k*_+7_)(*k*_-4_)(*k*_-10_)(*k*_+12_)(*k*_+15_) 
         _+_ *K*_1_(*k*_-2_)(*k*_+8_)(*k*_+6_)(*k*_+7_)(*k*_-4_)(*k*_-10_)(*k*_+12_)(*k*_-14_) 
         _+_ *K*_1_(*k*_-2_)(*k*_+8_)(*k*_+6_)(*k*_+7_)(*k*_-4_)(*k*_+11_)(*k*_-13_)(*k*_+15_) 
         _+_ *K*_1_(*k*_-2_)(*k*_+8_)(*k*_+6_)(*k*_+7_)(*k*_-4_)(*k*_+11_)(*k*_-13_)(*k*_-14_) 
         _+_ *K*_1_(*k*_-2_)(*k*_+8_)(*k*_+6_)(*k*_+7_)(*k*_-4_)(*k*_+11_)(*k*_+12_)(*k*_+15_) 
         _+_ *K*_1_(*k*_-2_)(*k*_+8_)(*k*_+6_)(*k*_+7_)(*k*_-4_)(*k*_+11_)(*k*_+12_)(*k*_-14_) 
         _+_ *K*_1_(*k*_+3_)(*k*_+8_)(*k*_+6_)(*k*_+7_)(*k*_+5_)(*k*_-10_)(*k*_-13_)(*k*_+15_) 
         _+_ *K*_1_(*k*_+3_)(*k*_+8_)(*k*_+6_)(*k*_+7_)(*k*_+5_)(*k*_-10_)(*k*_-13_)(*k*_-14_) 
         _+_ *K*_1_(*k*_+3_)(*k*_+8_)(*k*_+6_)(*k*_+7_)(*k*_+5_)(*k*_-10_)(*k*_+12_)(*k*_+15_) 
         _+_ *K*_1_(*k*_+3_)(*k*_+8_)(*k*_+6_)(*k*_+7_)(*k*_+5_)(*k*_-10_)(*k*_+12_)(*k*_-14_) 
         _+_ *K*_1_(*k*_+3_)(*k*_+8_)(*k*_+6_)(*k*_+7_)(*k*_+5_)(*k*_+11_)(*k*_-13_)(*k*_+15_) 
         _+_ *K*_1_(*k*_+3_)(*k*_+8_)(*k*_+6_)(*k*_+7_)(*k*_+5_)(*k*_+11_)(*k*_-13_)(*k*_-14_) 
         _+_ *K*_1_(*k*_+3_)(*k*_+8_)(*k*_+6_)(*k*_+7_)(*k*_+5_)(*k*_+11_)(*k*_+12_)(*k*_+15_) 
         _+_ *K*_1_(*k*_+3_)(*k*_+8_)(*k*_+6_)(*k*_+7_)(*k*_+5_)(*k*_+11_)(*k*_+12_)(*k*_-14_) 
         _+_ *K*_1_(*k*_+3_)(*k*_+8_)(*k*_+6_)(*k*_+7_)(*k*_-4_)(*k*_-10_)(*k*_-13_)(*k*_+15_) 
         _+_ *K*_1_(*k*_+3_)(*k*_+8_)(*k*_+6_)(*k*_+7_)(*k*_-4_)(*k*_-10_)(*k*_-13_)(*k*_-14_) 
         _+_ *K*_1_(*k*_+3_)(*k*_+8_)(*k*_+6_)(*k*_+7_)(*k*_-4_)(*k*_-10_)(*k*_+12_)(*k*_+15_) 
         _+_ *K*_1_(*k*_+3_)(*k*_+8_)(*k*_+6_)(*k*_+7_)(*k*_-4_)(*k*_-10_)(*k*_+12_)(*k*_-14_) 
         _+_ *K*_1_(*k*_+3_)(*k*_+8_)(*k*_+6_)(*k*_+7_)(*k*_-4_)(*k*_+11_)(*k*_-13_)(*k*_+15_) 
         _+_ *K*_1_(*k*_+3_)(*k*_+8_)(*k*_+6_)(*k*_+7_)(*k*_-4_)(*k*_+11_)(*k*_-13_)(*k*_-14_) 
         _+_ *K*_1_(*k*_+3_)(*k*_+8_)(*k*_+6_)(*k*_+7_)(*k*_-4_)(*k*_+11_)(*k*_+12_)(*k*_+15_) 
         _+_ *K*_1_(*k*_+3_)(*k*_+8_)(*k*_+6_)(*k*_+7_)(*k*_-4_)(*k*_+11_)(*k*_+12_)(*k*_-14_) 
         _+_ *K*_1_(*k*_+2_)(*k*_+3_)(*k*_+8_)(*k*_+13_)(*k*_+7_)(*k*_+5_)*K*_9_(*k*_+12_)(*k*_+15_) 
         _+_ *K*_1_(*k*_+2_)(*k*_+3_)(*k*_+8_)(*k*_+13_)(*k*_+7_)(*k*_+5_)*K*_9_(*k*_+12_)(*k*_-14_) 
         _+_ *K*_1_(*k*_+2_)(*k*_+3_)(*k*_+8_)(*k*_+13_)(*k*_+7_)(*k*_-4_)*K*_9_(*k*_+12_)(*k*_+15_) 
         _+_ *K*_1_(*k*_+2_)(*k*_+3_)(*k*_+8_)(*k*_+13_)(*k*_+7_)(*k*_-4_)*K*_9_(*k*_+12_)(*k*_-14_) 
         _+_ *K*_1_(*k*_+2_)(*k*_+3_)(*k*_+8_)(*k*_+13_)(*k*_-6_)(*k*_+5_)*K*_9_(*k*_+12_)(*k*_+15_) 
         _+_ *K*_1_(*k*_+2_)(*k*_+3_)(*k*_+8_)(*k*_+13_)(*k*_-6_)(*k*_+5_)*K*_9_(*k*_+12_)(*k*_-14_) 
         _+_ *K*_1_(*k*_+2_)(*k*_+3_)(*k*_+8_)(*k*_+13_)(*k*_-6_)(*k*_-4_)*K*_9_(*k*_+12_)(*k*_+15_) 
         _+_ *K*_1_(*k*_+2_)(*k*_+3_)(*k*_+8_)(*k*_+13_)(*k*_-6_)(*k*_-4_)*K*_9_(*k*_+12_)(*k*_-14_) 
         _+_ *K*_1_(*k*_+2_)(*k*_+3_)(*k*_+8_)(*k*_+13_)(*k*_+7_)(*k*_+5_)*K*_9_(*k*_-10_)(*k*_+15_) 
         _+_ *K*_1_(*k*_+2_)(*k*_+3_)(*k*_+8_)(*k*_+13_)(*k*_+7_)(*k*_+5_)*K*_9_(*k*_-10_)(*k*_-14_) 
         _+_ *K*_1_(*k*_+2_)(*k*_+3_)(*k*_+8_)(*k*_+13_)(*k*_+7_)(*k*_+5_)*K*_9_(*k*_+11_)(*k*_+15_) 
         _+_ *K*_1_(*k*_+2_)(*k*_+3_)(*k*_+8_)(*k*_+13_)(*k*_+7_)(*k*_+5_)*K*_9_(*k*_+11_)(*k*_-14_) 
         _+_ *K*_1_(*k*_+2_)(*k*_+3_)(*k*_+8_)(*k*_+13_)(*k*_+7_)(*k*_-4_)*K*_9_(*k*_-10_)(*k*_+15_) 
         _+_ *K*_1_(*k*_+2_)(*k*_+3_)(*k*_+8_)(*k*_+13_)(*k*_+7_)(*k*_-4_)*K*_9_(*k*_-10_)(*k*_-14_) 
         _+_ *K*_1_(*k*_+2_)(*k*_+3_)(*k*_+8_)(*k*_+13_)(*k*_+7_)(*k*_-4_)*K*_9_(*k*_+11_)(*k*_+15_) 
         _+_ *K*_1_(*k*_+2_)(*k*_+3_)(*k*_+8_)(*k*_+13_)(*k*_+7_)(*k*_-4_)*K*_9_(*k*_+11_)(*k*_-14_) 
         _+_ *K*_1_(*k*_+2_)(*k*_+3_)(*k*_+8_)(*k*_+13_)(*k*_-6_)(*k*_+5_)*K*_9_(*k*_-10_)(*k*_+15_) 
         _+_ *K*_1_(*k*_+2_)(*k*_+3_)(*k*_+8_)(*k*_+13_)(*k*_-6_)(*k*_+5_)*K*_9_(*k*_-10_)(*k*_-14_) 
         _+_ *K*_1_(*k*_+2_)(*k*_+3_)(*k*_+8_)(*k*_+13_)(*k*_-6_)(*k*_+5_)*K*_9_(*k*_+11_)(*k*_+15_) 
         _+_ *K*_1_(*k*_+2_)(*k*_+3_)(*k*_+8_)(*k*_+13_)(*k*_-6_)(*k*_+5_)*K*_9_(*k*_+11_)(*k*_-14_) 
         _+_ *K*_1_(*k*_+2_)(*k*_+3_)(*k*_+8_)(*k*_+13_)(*k*_-6_)(*k*_-4_)*K*_9_(*k*_-10_)(*k*_+15_) 
         _+_ *K*_1_(*k*_+2_)(*k*_+3_)(*k*_+8_)(*k*_+13_)(*k*_-6_)(*k*_-4_)*K*_9_(*k*_-10_)(*k*_-14_) 
         _+_ *K*_1_(*k*_+2_)(*k*_+3_)(*k*_+8_)(*k*_+13_)(*k*_-6_)(*k*_-4_)*K*_9_(*k*_+11_)(*k*_+15_) 
         _+_ *K*_1_(*k*_+2_)(*k*_+3_)(*k*_+8_)(*k*_+13_)(*k*_-6_)(*k*_-4_)*K*_9_(*k*_+11_)(*k*_-14_)}
β_23_ = {*K*_1_(*k*_-2_)(*k*_+8_)(*k*_+13_)(*k*_+7_)(*k*_+5_)(*k*_-10_)(*k*_+12_)(*k*_+15_) 
         _+_ *K*_1_(*k*_-2_)(*k*_+8_)(*k*_+13_)(*k*_+7_)(*k*_+5_)(*k*_-10_)(*k*_+12_)(*k*_-14_) 
         _+_ *K*_1_(*k*_-2_)(*k*_+8_)(*k*_+13_)(*k*_+7_)(*k*_-4_)(*k*_-10_)(*k*_+12_)(*k*_+15_) 
         _+_ *K*_1_(*k*_-2_)(*k*_+8_)(*k*_+13_)(*k*_+7_)(*k*_-4_)(*k*_-10_)(*k*_+12_)(*k*_-14_) 
         _+_ *K*_1_(*k*_-2_)(*k*_+8_)(*k*_+13_)(*k*_-6_)(*k*_+5_)(*k*_-10_)(*k*_+12_)(*k*_+15_) 
         _+_ *K*_1_(*k*_-2_)(*k*_+8_)(*k*_+13_)(*k*_-6_)(*k*_+5_)(*k*_-10_)(*k*_+12_)(*k*_-14_) 
         _+_ *K*_1_(*k*_-2_)(*k*_+8_)(*k*_+13_)(*k*_-6_)(*k*_-4_)(*k*_-10_)(*k*_+12_)(*k*_+15_) 
         _+_ *K*_1_(*k*_-2_)(*k*_+8_)(*k*_+13_)(*k*_-6_)(*k*_-4_)(*k*_-10_)(*k*_+12_)(*k*_-14_) 
         _+_ *K*_1_(*k*_+3_)(*k*_+8_)(*k*_+13_)(*k*_+7_)(*k*_+5_)(*k*_-10_)(*k*_+12_)(*k*_+15_) 
         _+_ *K*_1_(*k*_+3_)(*k*_+8_)(*k*_+13_)(*k*_+7_)(*k*_+5_)(*k*_-10_)(*k*_+12_)(*k*_-14_) 
         _+_ *K*_1_(*k*_+3_)(*k*_+8_)(*k*_+13_)(*k*_+7_)(*k*_-4_)(*k*_-10_)(*k*_+12_)(*k*_+15_) 
         _+_ *K*_1_(*k*_+3_)(*k*_+8_)(*k*_+13_)(*k*_+7_)(*k*_-4_)(*k*_-10_)(*k*_+12_)(*k*_-14_) 
         _+_ *K*_1_(*k*_+3_)(*k*_+8_)(*k*_+13_)(*k*_-6_)(*k*_+5_)(*k*_-10_)(*k*_+12_)(*k*_+15_) 
         _+_ *K*_1_(*k*_+3_)(*k*_+8_)(*k*_+13_)(*k*_-6_)(*k*_+5_)(*k*_-10_)(*k*_+12_)(*k*_-14_) 
         _+_ *K*_1_(*k*_+3_)(*k*_+8_)(*k*_+13_)(*k*_-6_)(*k*_-4_)(*k*_-10_)(*k*_+12_)(*k*_+15_) 
         _+_ *K*_1_(*k*_+3_)(*k*_+8_)(*k*_+13_)(*k*_-6_)(*k*_-4_)(*k*_-10_)(*k*_+12_)(*k*_-14_)}

**REFERENCES**

1. Varón-Castellanos R, Garcia-Moreno M, Garcia-Sevilla F, Ruiz-Galea MM, Garcia-Canovas F. Computerized derivation of the steady-state equations of enzyme reactions. Albacete: A5; 1995.

2. Rodriguez-Lopez JN, Ros JR, Varon R, Garcia-Canovas F. Oxygen Michaelis constants for tyrosinase. Biochem J. 1993;293(859):859-66. doi: 10.1042/bj2930859. pmid:8352753

3. Fenoll LG, Rodriguez-Lopez JN, Garcia-Molina F, Garcia-Canovas F, Tudela J. Michaelis constants of mushroom tyrosinase with respect to oxygen in the presence of monophenols and diphenols. Int J Biochem Cell Biol. 2002;34(332):332-6. doi: 10.1016/s1357-2725(01)00133-9. pmid:11854032
